# Flexible Ag_2_Se‐Based Thermoelectrics: Fundamentals, Processing, and Device Applications

**DOI:** 10.1002/adma.202518096

**Published:** 2026-06-29

**Authors:** Jie Qin, Yuchen Yang, Huangshui Ma, Yong Du, Min Hong, Qinfei Ke

**Affiliations:** ^1^ Faculty of Materials Technology Shanghai Institute of Technology Shanghai China; ^2^ Centre for Future Materials School of Science Engineering and Digital Technologies University of Southern Queensland Springfield Central Queensland Australia

**Keywords:** Ag_2_Se, energy harvesting, flexible films, thermoelectric devices, wearable electronics

## Abstract

Ag_2_Se is widely recognized as a leading n‐type thermoelectric material for flexible and wearable applications owing to its narrow band gap, intrinsically low lattice thermal conductivity, and unusual room‐temperature plasticity. This review systematically summarizes recent advances in Ag_2_Se‐based thermoelectrics, beginning with its fundamental crystal structures, defect chemistry, and electronic band features that govern its semiconducting and superionic transport behavior. Advanced performance‐enhancement strategies are discussed in detail, including nanostructuring, stoichiometry tuning, doping, and the incorporation of inorganic or organic second phases. The progress in fabrication techniques, including vacuum‐assisted filtration, screen printing, magnetron sputtering, thermal evaporation, and additive manufacturing, has also been highlighted. Scalability, flexibility, and mechanical durability are emphasized. Furthermore, the assembly and application of Ag_2_Se‐based flexible thermoelectric devices are reviewed, covering thermoelectric generators, Peltier coolers, electronic skins, and photo‐thermoelectric hybrids. These devices demonstrate strong potential for energy harvesting, localized cooling, and smart sensing. Additionally, the challenges of device stability, large‐area integration, and multifunctional system design are assessed. This review links material‐level insights with device‐level applications to accelerate the deployment of Ag_2_Se‐based thermoelectrics in sustainable energy and wearable electronics.

## Graphical abstract

Ag_2_Se‐based thermoelectrics are reviewed from fundamental transport mechanisms to flexible device integration. Crystal structure, defect chemistry, and band features are correlated with performance optimization strategies and scalable fabrication routes. Recent progress in flexible generators, cooling devices, and smart sensors highlights Ag_2_Se as a key platform for wearable and sustainable energy technologies.

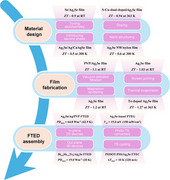

## 1 Introduction

Driven by the rapid expansion of artificial intelligence (AI) and the Internet of Things, the demand for smart wearable devices and their corresponding power sources is steadily increasing [1, 2, 3, 4]. Traditional power sources, such as lithium‐ion batteries, have limitations including the need for frequent recharging and/or replacement [5, 6, 7]. These constraints pose significant challenges to the seamless integration of power solutions into wearable technologies [8, 9, 10]. Thermoelectric (TE) technology can realize mutual conversion between heat and electricity, and therefore provides a promising solution for power generation [11, 12, 13]. The performance of a TE material is gauged by its dimensionless figure of merit (*ZT*  =  *S* ^2^ *σ* *T*/*κ*) [14, 15, 16, 17], where *S*, *σ*, *κ*, and *T* are the Seebeck coefficient, electrical conductivity, thermal conductivity, and absolute temperature, respectively [18, 19, 20, 21, 22]. A high *ZT* requires a high *S* ^2^ *σ*, but a low *κ* [23, 24, 25, 26, 27].

Traditional bulk TE materials, while effective, commonly lack the mechanical flexibility for new applications [28, 29]. Flexible TE devices (FTEDs), assembled from corresponding flexible TE films, can adapt to various shapes and non‐planar surfaces [30, 31, 32], with the advantages of operating without moving parts [33, 34, 35], good durability and reliability [36, 37], making them suitable for applications in wearable technology [38, 39, 40, 41], especially in flexible electronics [42, 43, 44] and portable power generation [45, 46, 47]. This capability makes them viable alternatives to conventional fossil fuel‐based energy sources, particularly in applications requiring reliable and portable energy solutions [48, 49].

Among the various TE materials, Ag_2_Se is an n‐type semiconductor with a narrow bandgap and exists in two primary phases: orthorhombic *β*‐Ag_2_Se at low temperature and cubic *α*‐Ag_2_Se at high temperature, and the phase transition between the two forms occurs at around 407 K [50, 51, 52, 53]. The orthorhombic *β*‐Ag_2_Se is stable at room temperature (RT) and exhibits excellent TE properties, due to its high carrier mobility (*µ*) and low *κ*  [54, 55]. These ideal properties make Ag_2_Se gain significant attention, particularly suitable for applications in FTEDs and wearable electronics [56, 57, 58].

Recently, considerable attention has been paid to the TE properties and mechanical flexibility of Ag_2_Se films [59, 60]. Methods, such as doping [61, 62], nanostructuring [50, 63], and the incorporation of second‐phase [53, 64], have been explored to optimize the TE properties of these films. For instance, doping with elements such as Te has been shown to enhance *S* and *σ* [61], whereas nanostructuring can significantly reduce *κ* through enhanced phonon scattering. Based on the above‐mentioned strategies, the TE properties and mechanical flexibility of flexible Ag_2_Se‐based films have been significantly enhanced. Thus, Ag_2_Se‐based flexible TE films have huge potential for the application in self‐powered wearable devices and efficient cooling systems for portable electronics [65, 66, 67]. However, challenges remain in the fabrication and stability of these films. Ensuring a consistent performance under various operational conditions, addressing potential degradation mechanisms, and developing scalable fabrication processes are crucial for the widespread adoption of flexible Ag_2_Se‐based TE films [68, 69, 70]. Therefore, a review summarizing the recent progress, existing challenges, and prospects of flexible Ag_2_Se‐based TE films is highly desirable.

**FIGURE 1:**
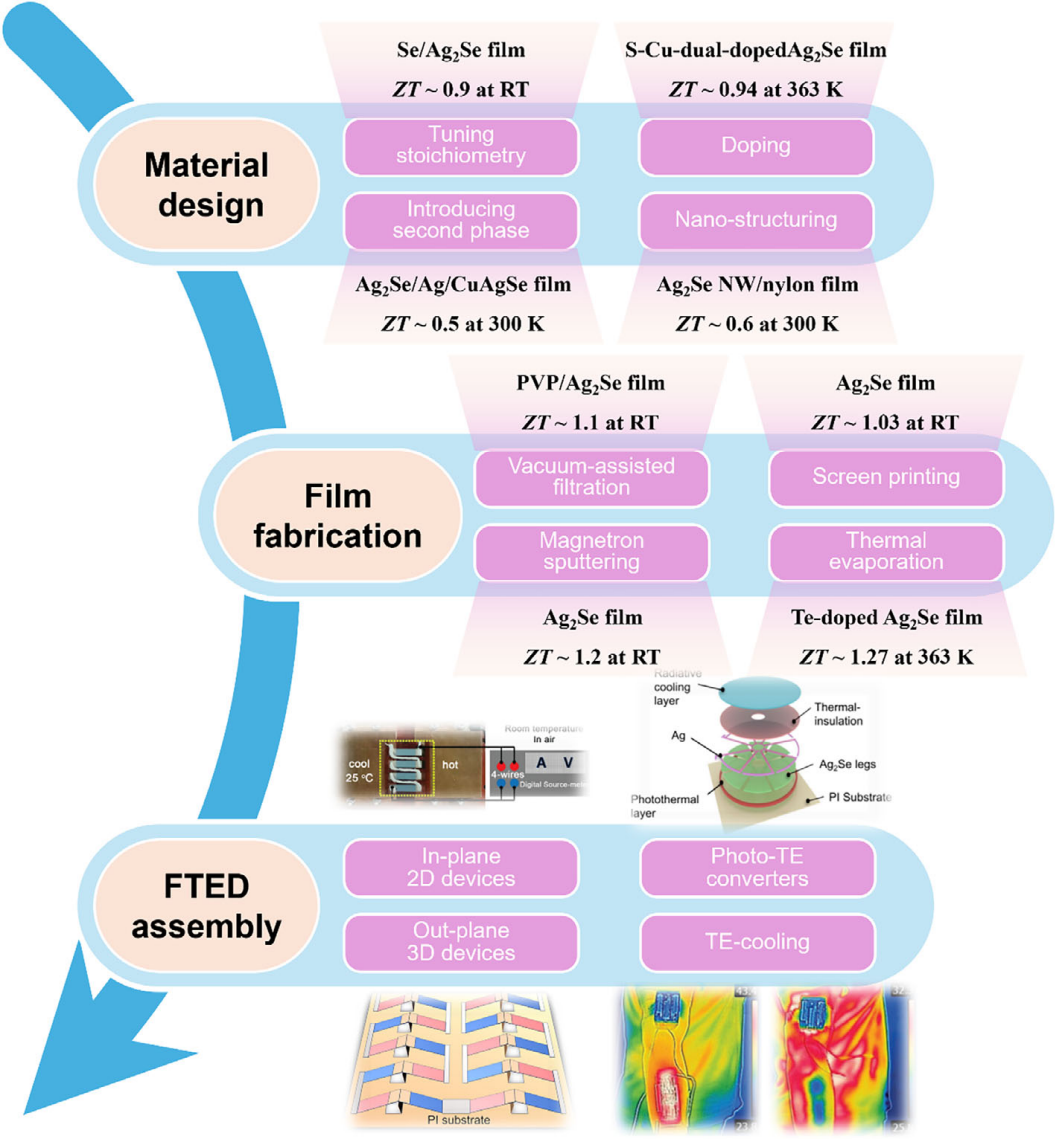
Strategies for advancing flexible Ag_2_Se‐based thermoelectrics: material design, film fabrication, and FTED assembly [7, 50, 55, 56, 61, 62, 64, 71, 72, 73, 74, 75]. Image for “In‐plane 2D devices”: Reproduced with permission [75]. Copyright 2022, John Wiley & Sons. Image for “Photo‐TE converters”: Reproduced under the terms of the Creative Commons CC‐BY Creative Commons Attribution 4.0 International license (https://creativecommons.org/licenses/by/4.0) [64]. Copyright 2024, The Authors, published by Springer Nature. Image for “Out‐of‐plane 3D devices”: Reproduced with permission [76]. Copyright 2025, Springer Nature. Image for “TE‐cooling”: Reproduced with permission [73]. Copyright 2021, John Wiley & Sons.

This review aims to provide an overview of recent progress in the development of flexible Ag_2_Se‐based TE films. It covers the fundamental properties of Ag_2_Se, including its thermodynamics, crystal structure, and electronic properties. Various synthesis and fabrication techniques, performance optimization strategies, and potential applications of flexible Ag_2_Se‐based TE films in wearable devices are also discussed. Figure 1 summarizes the reported TE performance of typical flexible Ag_2_Se‐based films. By highlighting the current existing challenges and future research directions, this review offers insights into flexible Ag_2_Se‐based TE films.

## 2 Fundamentals of Ag_2_Se‐Based Thermoelectrics

### 2.1 Crystal Structures

**FIGURE 2:**
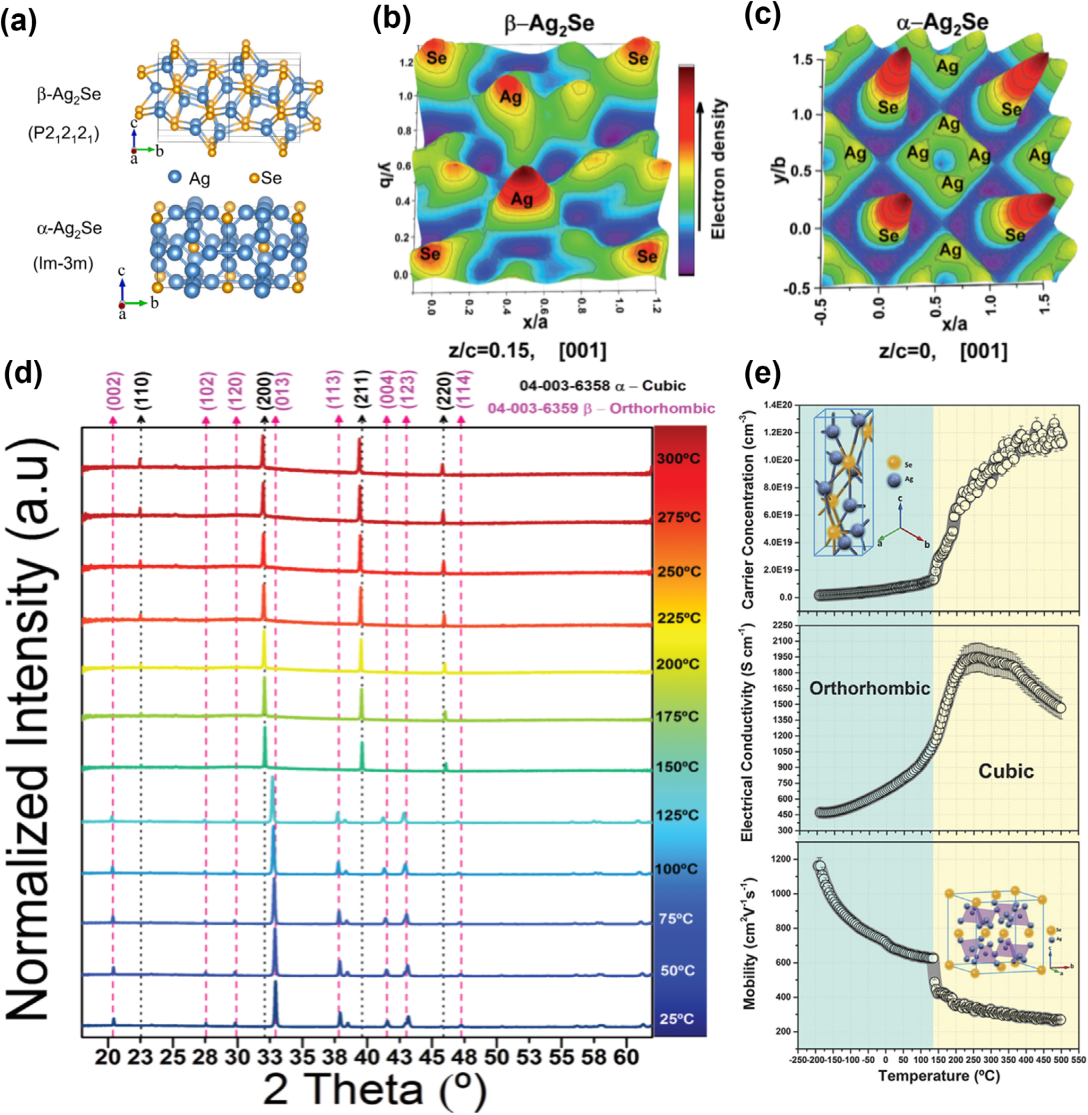
(a) The crystal structure of the *β*‐Ag_2_Se phase is shown at the top, while the *α*‐Ag_2_Se phase at high temperatures (above 407 K) is depicted at the bottom, reproduced with permission [77]. Copyright 2021, Elsevier. The Fourier maps illustrating the electron density distributions for the atoms Ag and Se are shown for the (b) *β*‐Ag_2_Se and (c) *α*‐Ag_2_Se phases. (b,c) Reproduced with permission [55]. Copyright 2020, Royal Society of Chemistry. (d) SR‐GIXRD patterns with temperatures ranging from 25°C (light blue) to 300°C (red) while measuring every 25°C. (e) Temperature‐dependent *n*, *σ*, and *µ* for the Ag_2_Se film. (d,e) Reproduced with permission [72]. Copyright 2017, John Wiley & Sons.

Ag_2_Se exhibits two distinct crystal structures depending on the temperature (Figure 2a). At RT and up to 407 K, it exists in the low‐temperature *β*‐phase with an orthorhombic structure, typically described by the space group *P*2_1_2_1_2_1_ [77]. In this structure, Ag atoms occupy different coordination environments: some adopt tetrahedral‐like surroundings, whereas others are nearly triangular. The lattice parameters are *a* = 4.33 Å, *b* = 7.06 Å, and *c* = 7.76 Å [77].

Above approximately 407 K, *β*‐Ag_2_Se transforms into the high‐temperature *α*‐Ag_2_Se, which adopts a cubic antifluorite‐type structure (*Im* $\bar{3}$ *m*) [77]. In this phase, the Se anions form a rigid backbone, while silver cations become highly disordered and mobile, leading to liquid‐like ionic conductivity [78]. This superionic behavior is a hallmark of *α*‐Ag_2_Se and makes it particularly attractive for applications in solid‐state ionic devices. The phase transition between *β*‐Ag_2_Se and *α*‐Ag_2_Se is central to its functional properties [79].

Furthermore, electron density distributions were also investigated in *β*‐Ag_2_Se and *α*‐Ag_2_Se phases, shown in Figure 2b,c [55]. In the *β*‐Ag_2_Se phase, the Se atom is positioned on the *z/c* = 0.15 plane with coordinates *x/a* = 0.112 and *y/b* = 0.998, while in the *α*‐Ag_2_Se phase, Se is located on the *z/c* = 0 plane with coordinates *x/a* = 0 and *y/b* = 0 [55].

The structural transition has been comprehensively examined by in situ synchrotron radiation grazing incidence X‐ray diffraction (SR‐GIXRD) measurements, shown in Figure 2d [72]. Diffraction patterns collected between RT and 300°C reveal a gradual disappearance of orthorhombic reflections (purple) and the simultaneous emergence of cubic reflections (black), providing direct evidence of the *β*‐to‐*α* phase transition. The carrier concentration (*n*), *σ*, and *µ* are plotted as functions of temperature (Figure 2e). In the orthorhombic *β*‐phase, *n* and *σ* remain low, consistent with semiconducting behavior. However, upon entering the cubic *α*‐phase, both parameters increase sharply, with *σ* increasing to values characteristic of a superionic conductor [72].

**FIGURE 3:**
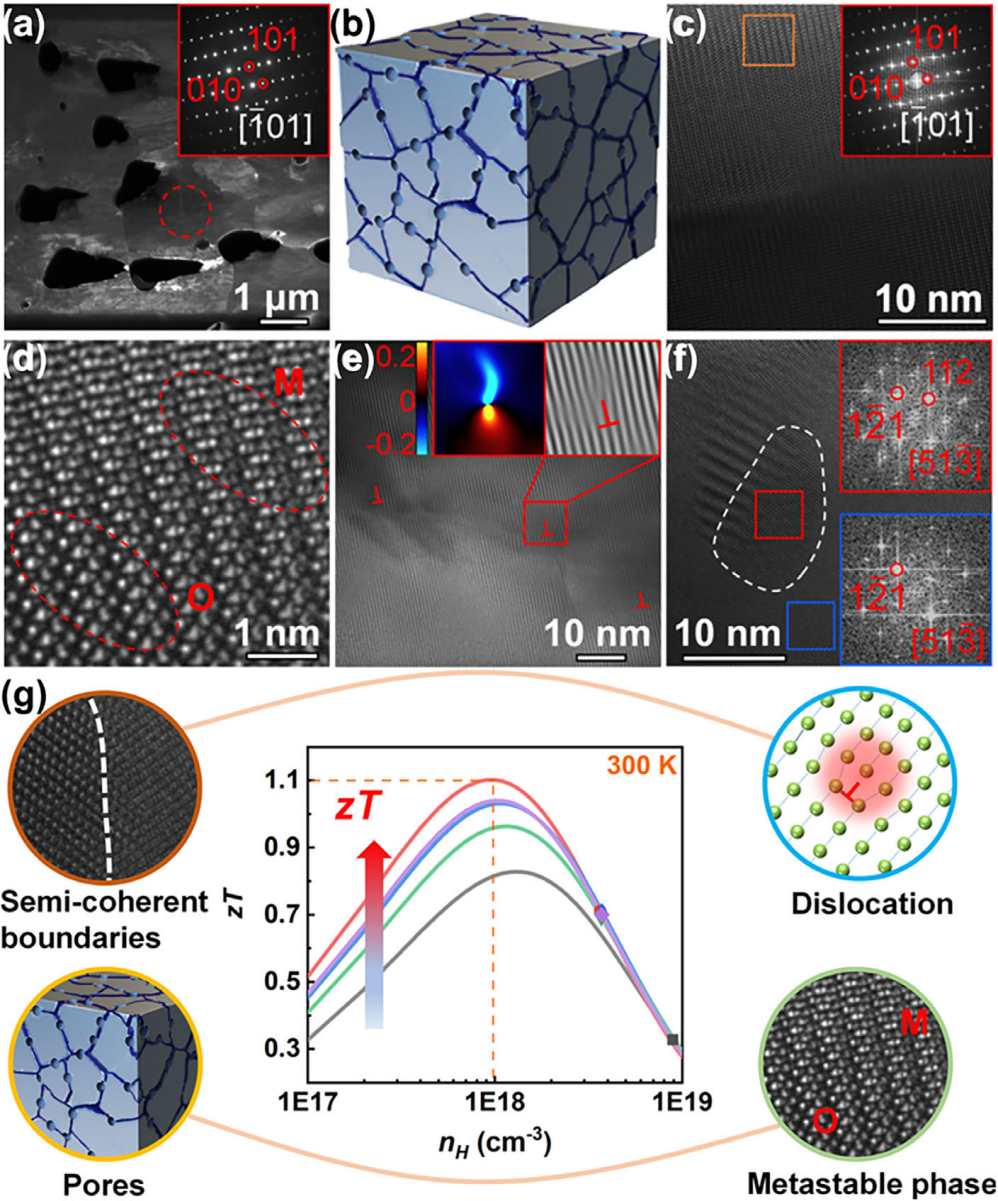
(a) Dark‐field TEM image revealing the grains and nanopores, with an inset displaying the SAED pattern. (b) Schematic illustration of nanopores. (c) HRTEM image with the inset showing an FFT pattern along the [$\bar{1}$01] zone axis. (d) Enlarged view of the framed area in (c), revealing the coexistence of orthorhombic (O) and the metastable (M) phases. (e) HRTEM image dislocations, with the inset showing an inverse FFT image. (f) Representative nanoscale grain along the [51$\bar{3}$]. (g) Optimized *ZT* values associated with different structural defects. (a–g) Reproduced with permission [80]. Copyright 2020, American Chemical Society.

Structural defects and pores significantly influence the TE performance of Ag_2_Se. Chen et al. [80] have demonstrated that porous Ag_2_Se samples annealed at 473 K (AS‐473) with hierarchical structures, including nanosized grains, metastable phases, grain boundaries, localized strains, and dense dislocations, exhibit enhanced TE properties (Figure 3). The transmission electron microscopy (TEM) image (Figure 3a) reveals the Ag_2_Se grains interspersed with pores, and the crystalline orientation is also confirmed by the corresponding selected area electron diffraction (SAED) pattern. A schematic representation of the nanopores located around grain interfaces is illustrated in Figure 3b, where porosity contributes to phonon scattering. Figure 3c shows a high‐resolution TEM (HRTEM) image with the zone axis along the [$\bar{1}$01], which reveals semi‐coherent grain boundaries. Figure 3d provides a zoomed‐in view of *β*‐Ag_2_Se and the coexisting metastable phase, highlighting the structural heterogeneity at the nanoscale [80].

Structural defects are further resolved in Figure 3e,f. The HRTEM image in Figure 3e shows dense dislocations embedded in the grain, whereas the inverse FFT captures a typical edge dislocation accompanied by localized strain fields, which could enhance phonon scattering. Figure 3f shows typical nanoscale grains with semi‐coherent interfaces, suggesting partial lattice matching between adjacent grains [80].

Collectively, pores, semi‐coherent boundaries, dislocations, and metastable phases are schematically summarized around the central plot in Figure 3g. By adjusting the sintering temperature of the Ag_2_Se samples, such structural defects and pores could be optimized, contributing to a low *κ*_l_ of 0.35 Wm^−1^K^−1^ and a *ZT* of ∼ 0.7 at 300 K for AS‐473 sample. This evidence demonstrates the defect‐property relationship in Ag_2_Se, where the controlled introduction of structural imperfections serves as a strategy to suppress *κ* while retaining favorable electrical transport, boosting the overall TE performance [80].

**FIGURE 4:**
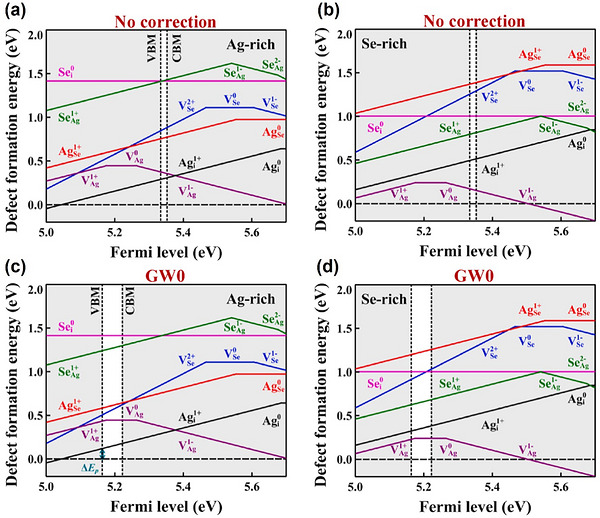
Formation energies calculated using PBE + U without correction under (a) Ag‐rich and (b) Se‐rich conditions. Formation energies calculated using GW_0_‐corrected band edges under (c) Ag‐rich and (d) Se‐rich conditions. The gradient of each line indicates the charge state of the defect. Vertical dotted lines denote the locations of the valence band maximum (VBM) and conduction band maximum (CBM). The p‐type dopability window (Δ*E*_p_) in (c) is specified at the VBM. (a–d) Reproduced with permission [81]. Copyright 2023, Elsevier.

Theoretical calculations further elucidated the defect chemistry in Ag_2_Se, emphasizing the significant roles of Ag interstitials and vacancies (Figure 4) [81]. Ag interstitials have notably low formation energies, favoring their formation during synthesis. Ag interstitials impact lattice distortions to a lesser extent than vacancies, favoring the structural stability of the material. Additionally, electron localization function (ELF) analysis revealed stable chemical bonding interactions formed by interstitial Ag atoms with surrounding Se and Ag atoms, thus stabilizing the lattice structure. Ag interstitials also drive the Fermi level (*E*_f_) closer to the conduction band, thereby contributing to the experimentally observed intrinsic n‐type conductivity of Ag_2_Se [81]. Such a detailed understanding of defect chemistry provides valuable insights for optimizing TE performance through precise defect engineering and controlled doping strategies.

### 2.2 Band Structures

**FIGURE 5:**
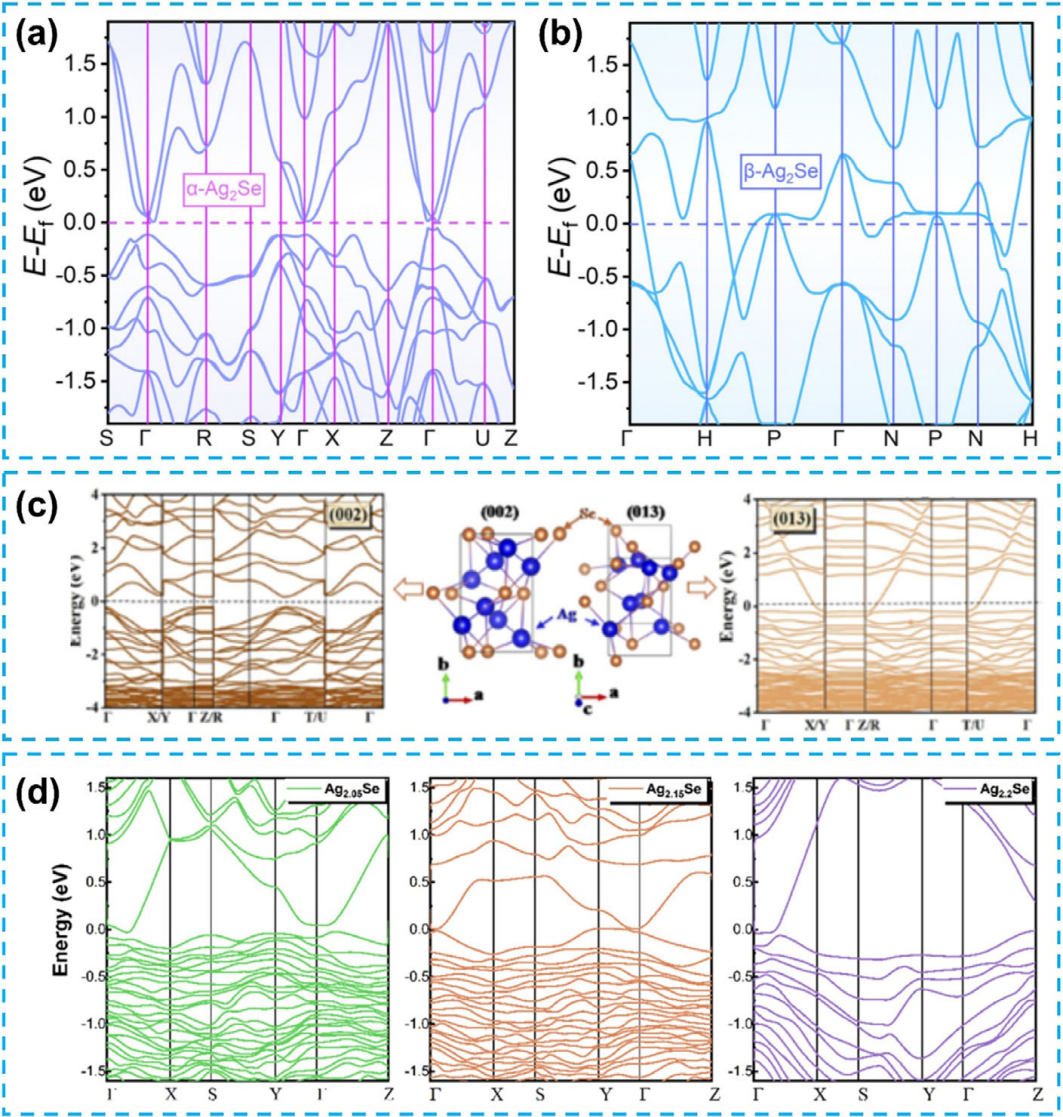
Band structures of (a) *α*‐Ag_2_Se and (b) *β*‐Ag_2_Se, reproduced with permission [65]. Copyright 2023, Royal Society of Chemistry. (c) Band structures of two crystal models of Ag_2_Se based on the electrical transport along planes (002) and (013), reproduced with permission [74]. Copyright 2022, Royal Society of Chemistry. (d) Calculated band structures for Ag_2_Se with Ag/Se ratios of 2.05, 2.15, and 2.20, reproduced with permission [82]. Copyright 2023, American Chemical Society.

The band structures of Ag_2_Se were investigated by calculations [65]. The calculated bandgap (*E*_g_) was small and matched the measured value of 0.03 eV (Figure 5a,b). The calculated density of states indicates that the conduction band near *E*_f_ is commonly dominated by Ag‐5s state, accompanied by additional weak contributions from Se‐4p and Ag‐4d states. Although the *E*_f_ of Ag_2_Se is positioned at the mid‐bandgap, the volatilization of selenium during the sintering process induces a substantial number of intrinsic Se vacancies, which provide electrons to the system. Furthermore, the migration of Ag ions increases the number of Ag interstitials, which contributes to the generation of free electrons. Overall, these Se vacancies and Ag interstitials lead to n‐type electrical transport. This explains the n‐type characteristics (*S* < 0) of the Ag_2_Se‐based materials [65].

Through strategic microstructural adjustments, flexible n‐type Ag_2_Se thin films were developed, achieving a power factor (*PF* = *S* ^2^ *σ*) of 2160 µWm^−1^K^−2^ at 348 K [74]. The electrical transport characteristics of Ag_2_Se across (002) and (013) crystallographic planes with the resulting band structures, using Density Functional Theory (DFT) calculations, were shown in Figure 5c. The band structure of Ag_2_Se (002) reveals a bandgap of approximately 0.35 eV, suggesting semiconductor properties, whereas Ag_2_Se (013) displays a band structure akin to that of a semi‐metal. These findings indirectly imply that Ag_2_Se films oriented along the (002) plane are likely to exhibit a lower *σ* and higher *S* when compared with those preferentially oriented along the (013) plane [74].

Similarly, a flexible Ag_2_Se film was fabricated on a polyimide (PI) membrane using a thermal diffusion method with various stoichiometric ratios of Ag/Se [82]. The band structures of Ag_2_Se with various Ag/Se ratios, as shown in Figure 5d, exhibit the intrinsic characteristics of narrow band gap semiconductors. The band structure facilitates electron transport in semiconductors, which is beneficial for increasing *σ*.

### 2.3 Electrical Transport Properties

The *σ* of TE materials can be calculated based on the Equation (1) as follows [83]:

(1) σ=neμ

For a degenerate semiconductor, *S* can be calculated [83]:

(2) $$S=m^{*}T\frac{8\pi^{2}{k_{B}}^{2}}{3{\mathrm{eh}}^{2}}{\left(\frac{\pi }{3n}\right)}^{\frac{2}{3}}$$

where *e* represents the electron charge, *k*_B_ is the Boltzmann constant, *m* ^*^ denotes the effective mass, and *h* is Planck's constant [84, 85]. Herein, *µ* and *m* ^*^ can be described by the following equations [86]:

(3) $$\mu \propto 1/{m_{b}}^{*\frac{5}{2}}$$

(4) $$m^{*}={N_{V}}^{\frac{2}{3}}{m_{b}}^{*}$$

where *N*_V_ and *m*_b_^*^ are the band degeneracy and effective mass of a single valley, respectively [87, 88, 89].

### 2.4 Thermal Properties

Among the various parameters influencing TE behavior, *κ* is particularly critical, which was comprised of electronic thermal conductivity (*κ*_e_) and lattice thermal conductivity (*κ*_l_), expressed as [83, 90, 91]:

(5) $$\kappa =\kappa_{e}+\kappa_{l}$$

*κ*_e_ can be calculated using the Wiedemann‐Franz relation, and *κ*_l_ was given via the kinetic formula, given below [90]:

(6) $$\kappa_{e}=L\sigma T$$

(7) $$\kappa_{l}=Cvl/3=Cv^{2}\tau /3$$

where *C*, *v*, *l*, and *τ* represent the specific heat, sound velocity, mean free path (*l* = *vτ*), and scattering time, respectively.

Experimentally, *κ* is commonly measured using the following methods: (1) the combined measurement of the materials’ density, thermal diffusivity, and specific heat capacity, (2) the 3*ω* method, (3) the hot‐disk (transient plane source) technique, (4) a transient photo‐electro‐thermal method, (5) the hot wire technique, etc. For instance, *κ* can be derived from the following equation [92, 93, 94]:

(8) $$\kappa =\rho \lambda C_{P}$$

where *ρ* and *λ* refer to the density and thermal diffusivity, and *C*_p_ represents the heat capacity, which could be either experimentally determined or approximated according to the Dulong–Petit law [95, 96, 97, 98]. Recent investigations have revealed that the phase transitions may cause deviations in the estimated *ZT* values [99, 100, 101]. According to differential scanning calorimetry measurements, Ag_2_Se exhibits a markedly slower phase transition process than Ag_2_S, Cu_2_Se, and Cu_2_S [102, 103, 104, 105, 106]. As a result, the *λ* of Ag_2_Se was found to remain nearly unchanged throughout the phase transition process [107, 108]. Nevertheless, Xiao et al. [109], systematically investigated the TE performance of Ag_2_Se during phase transition and revealed that *λ* sharply decreased to as low as 0.098 mm^2^/s at 408 K, followed by an increase with increasing temperature. In parallel, a maximum *ZT* of approximately 0.23 emerged around the phase transition point, which is ∼ 30% higher than the value at ∼ 393 K, highlighting the critical role of the transition in enhancing TE performance [109]. However, uncertainties remain regarding the phase transition behavior of Ag_2_Se.

The main effective strategies to reduce *κ* in Ag_2_Se include (1) compositing with organic materials [110], (2) dimensional reduction of Ag_2_Se [111, 112], (3) multilayered heterogeneous architecture [113, 114], (4) alloying and doping [61, 115, 116], and (5) phase transition tuning [109], which are discussed in the following sections.

### 2.5 Mechanical Properties

For flexible TE materials, given that flexible electronics are subject to inevitable bending and even folding during practical applications, especially with human activities, excellent mechanical performance is crucial along with superior TE performance [31, 117, 118]. Strong mechanical properties ensure that flexible TE materials can withstand various forms of physical damage in real‐world applications, thereby enhancing their reliability and lifespan [119, 120, 121, 122]. However, the compressive and tensile strengths of bulk Ag_2_Se are relatively low [123, 124, 125]. Additionally, like most inorganic semiconductors, bulk Ag_2_Se is hard and brittle, which makes it difficult to impart flexibility [126, 127, 128, 129]. Therefore, reducing the dimensionality of Ag_2_Se, e.g., converting it into thin films, is the key step toward improving its flexibility and enabling its application in flexible electronics.

**FIGURE 6:**
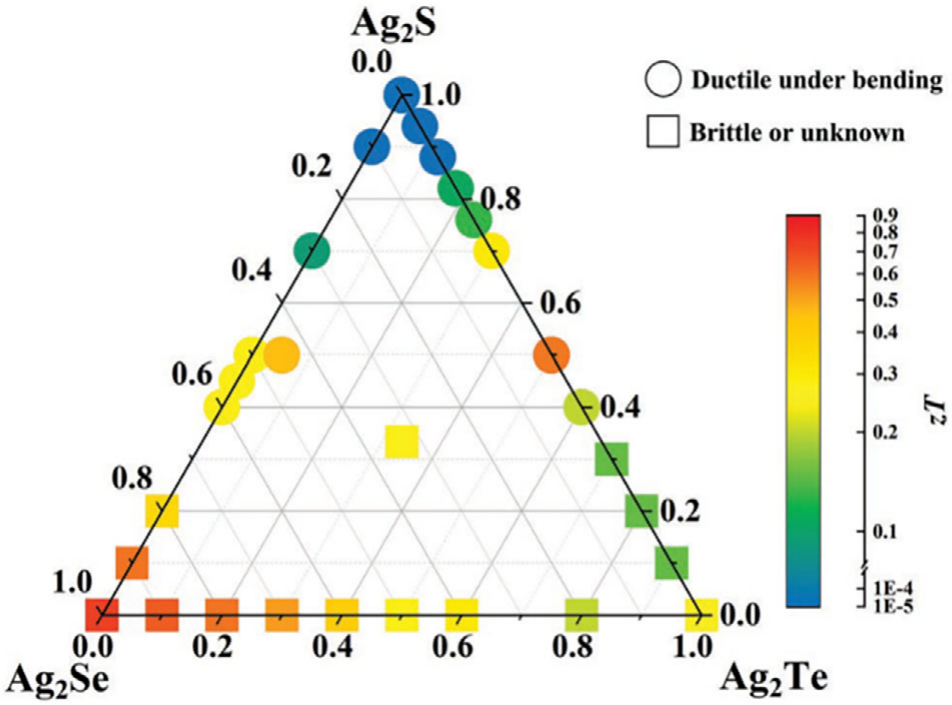
*ZT* value and plastic deformability of Ag_2_X‐based (X = S, Se, Te) materials around RT, reproduced with permission [126]. Copyright 2024, John Wiley & Sons.

The Ag_2_X (X = S, Se, Te) family is distinguished from most other inorganic TE materials by its exceptional ability to undergo plastic deformation around RT, which can be notably observed in Ag_2_S and more recently in the alloyed Ag_2_Se (Figure 6) [126, 130]. This metal‐like plasticity originates from favorable slip‐dominated deformation rather than brittle fracture, which is governed by a low slip barrier energy and high cleavage energy. At the atomic scale, the multicentered and spatially diffuse Ag‐Se bonds can dynamically rearrange under mechanical loading, maintaining structural integrity through continuous bond breaking and forming processes, thus facilitating plastic deformation [126].

**FIGURE 7:**
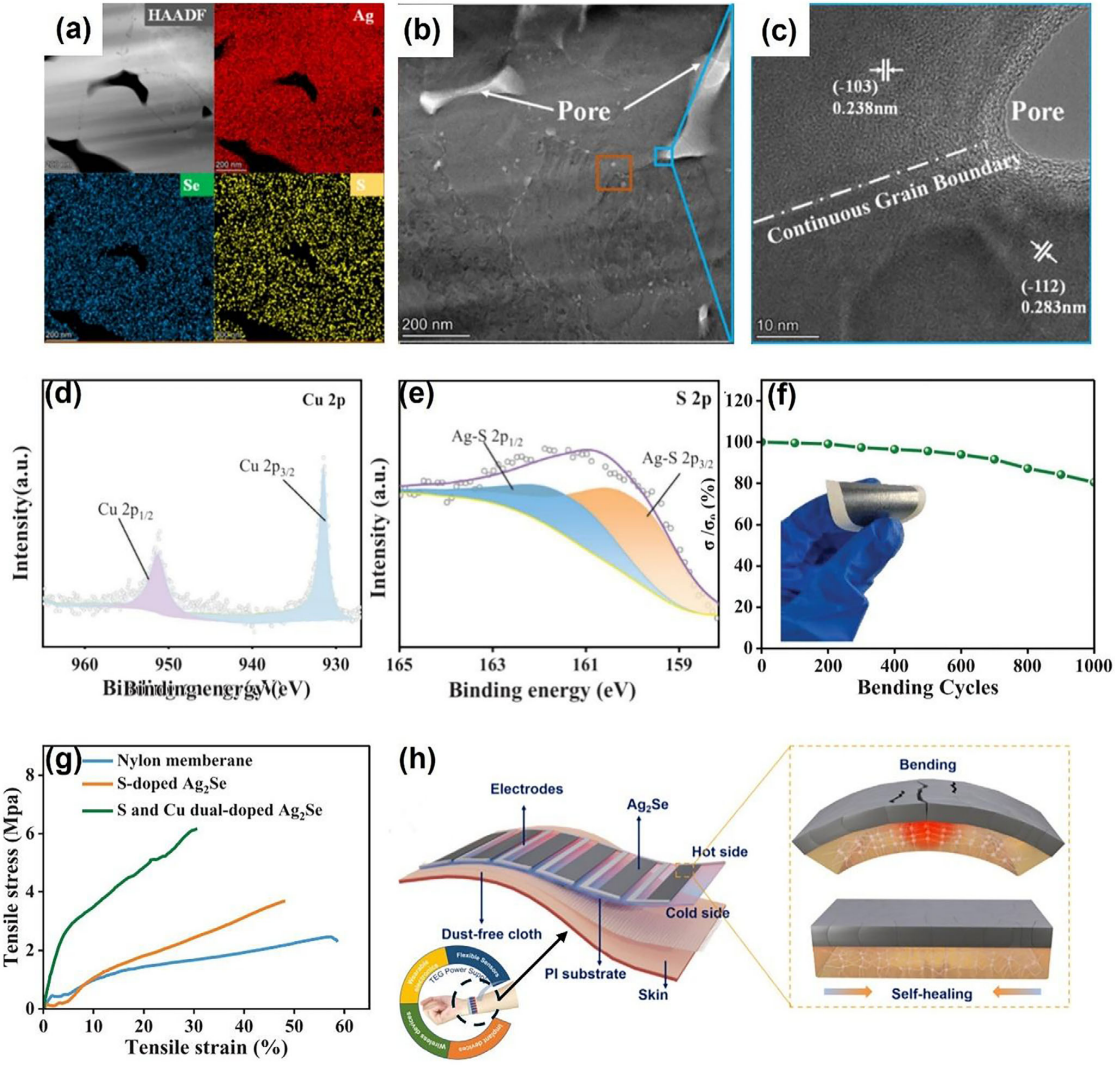
(a) HAADF image accompanied by elemental EDS mappings of Ag, Se, and S; (b) HAADF image of another representative region; (c) magnified view corresponding to the blue‐marked area in (b). (a–c) Reproduced with permission [131]. Copyright 2023, American Chemical Society. (d,e) XPS characterization of S/Cu dual‐doped Ag_2_Se nanomaterials, reproduced with permission [62]. Copyright 2024, John Wiley & Sons. (f) cyclic bending tests and (g) stress‐strain curves of S/Cu‐dual doped Ag_2_Se films; (h) Schematic diagram illustrating the structure of such in‐plane Ag_2_Se‐based wearable FTED, together with the associated self‐healing mechanism. (f‐h) Reproduced with permission [132]. Copyright 2022, Elsevier.

The mechanical properties of Ag_2_Se have been intensively studied in recent years, particularly using doping strategies involving Cu, S, and other elements. For example, Wu et al. [131] fabricated flexible Ag_2_S_1‐x_Se_x_ films on nylon membranes (Figure 7a–c), and the *σ* of the film remained at 94.6% after 2000 bends (bending radius of 4 mm). Additionally, flexible Ag_2_Se‐based TE films were fabricated via a sequential process combining vacuum‐assisted filtration and hot‐pressing, in which synergistic dual‐element doping with S and Cu was employed to enhance their mechanical flexibility [62]. The X‐ray photoelectron spectroscopy (XPS) spectra, as shown in Figure 7d,e, confirm the successful dual doping of S and Cu in Ag_2_Se. Cyclic bending measurements further verify the good flexibility of the film, with *σ* showing a 19.6% reduction after 1000 bends around a 5 mm radius rod (Figure 7f). Figure 7g demonstrates that the S/Cu‐dual doped Ag_2_Se films displayed a significantly enhanced tensile strength and Young's modulus than those without doping [62]. This mechanical performance can be attributed to the synergistic contribution of the continuous Ag_2_S_1‐x_Se_x_ solid‐solution framework and the compliant nylon substrate. Nevertheless, the bending flexibility is relatively lower than that reported in other Ag_2_Se‐based systems, due to the insufficient S content, as S incorporation has been demonstrated to effectively enhance ductility and strain tolerance in Ag_2_Se‐based matrices [62].

Mechanistically, Cu doping specifically enhances the plastic deformation of Ag_2_Se by introducing dense dislocations and nanophases (CuAgSe), which lower the slip barriers and impede crack propagation. Moreover, the densely nano‐twinned structures within CuAgSe promote the formation of dislocations, further improving flexibility. Hou et al. [132], presented a self‐healing FTED, which was constructed using Cu‐doped Ag_2_Se films (Figure 7h). The FTED exhibited good flexibility, with total resistance increasing by less than 5% after 1000 bending cycles. Notably, the resistance fully recovered to its initial value within 10 h after bending, revealing a self‐healing capability that was related to the viscoelastic nature of the PI substrate and the size of inorganic grain [132].

To provide a more quantitative assessment of the mechanical properties, the bending durability (as a representative example) of flexible Ag_2_Se‐based TE films reported in the literature has been systematically summarized, as shown in Table 1. Most flexible Ag_2_Se‐based TE films maintain more than 90% of their initial *σ* (*σ*_0_) after being bent at different bending radii (4–6 mm), indicating excellent mechanical robustness under repeated deformation. Compared with pristine Ag_2_Se, elemental doping (such as Ga, Cu, Te, and S) leads to improved *σ*/*σ*_0_, which can be attributed to dopant‐induced lattice distortion, refined grain structures, and enhanced strain accommodation. Composite and interface engineering further strengthen the mechanical durability, as most composite systems, such as carbon‐ or polymer‐modified Ag_2_Se films, consistently exhibit high *σ*/*σ*_0_ values even under small bending radii or after extended cycling. In contrast, films with reduced *σ*/*σ*_0_ may be associated with excessive porosity, weak interparticle bonding, or non‐optimized organic components, highlighting the sensitivity of mechanical reliability to microstructural integrity. The data shown in Table 1 clearly demonstrate that rational microstructural tuning through doping, composite, and interface engineering is an effective strategy for fabricating mechanically durable Ag_2_Se‐based films without sacrificing their TE performance.

**TABLE 1:** Bending durability of representative flexible Ag_2_Se‐based TE films.

Materials	Bending times	Bending radius (mm)	*σ*/*σ*_0_ (%)
Se‐doped Ag_2_S [131]	2000	4	94.6
S‐doped Ag_2_Se [133]	1000	4	94.4
S‐doped Ag_2_Se [134]	1000	6	> 91 (*PF*)
Te‐doped Ag_2_Se [76]	1000	5	96
Te‐doped Ag_2_Se [61]	1000	6.3	97.5
Ga‐doped Ag_2_Se [135]	1000	4	97
Cu‐doped Ag_2_Se [132]	1000	5	> 95
Cu‐doped Ag_2_Se [136]	1000	4	96.6
S/Cu‐doped Ag_2_Se [62]	1000	5	80.4
Ag_2_Se [137]	1000	4	94
Ag_2_Se [138]	1000	5	92 (*PF*)
Ag_2_Se [139]	2000	3	70.9
Ag/Ag_2_Se [140]	1000	4	94.8
SWCNT/Ag_2_Se [111]	1000	5	95
SWCNT/Ag_2_Se [141]	1000	4	94
rGO/Ag_2_Se [142]	500	4	94.62
rGO/Ag_2_Se [39]	1500	5	96.9
Ag_2_Se/carbon [143]	1500	10	86
Ag/Ag_2_Se/graphene [144]	800	4	94.1
Ag_2_Se/Ag/CuAgSe [7]	1000	4	93
PEDOT/Ag_2_Se/CuAgSe [145]	1000	4	92 (*PF*)
Ag/PVP/Ag_2_Se [110]	1000	4	93
Ag_2_Se/Ag/PVP [146]	1500	4	96.5
Ag_2_Se/Ag/PEDOT [147]	1000	4	94.5
Ag_2_Se/Se/PPy [53]	1000	4	93.5
PVP/Ag_2_Se [71]	1000	4	94.5
PEI/Ag_2_Se [57]	1000	6	93.5
PVP/Ag_2_Se/MC [90]	1000	4	93.76
Ag_2_Se [148]	1000	4	90.7 (*PF*)
Ag_2_Se [50]	1000	4	93
Ag_2_Se [149]	4000	4	91.52
Ag_2_Se [150]	1000	4	86.3

## 3 Preparation Methods of Ag_2_Se Materials

The morphology of Ag_2_Se plays a crucial role in enhancing its TE performance [50]. By controlling the morphology of Ag_2_Se, such as nanowires [50], nanorods [151], and nanoparticles [62], the TE properties of the material can be effectively improved, thereby increasing its potential for applications in flexible and wearable electronic devices.

### 3.1 Ag_2_Se Nanowire Synthesis

Ag_2_Se nanowires exhibit high *σ* and excellent flexibility owing to their 1D structure, making them ideal for flexible devices. The high aspect ratio of the nanowires allows for a more continuous electron transport pathway, reducing scattering and improving *σ*, which helps enhance the *ZT* value. Common fabrication methods, such as wet‐chemical synthesis using a Se or Ag nanowire template, are used to produce high‐quality Ag_2_Se nanowires [63, 150].

**FIGURE 8:**
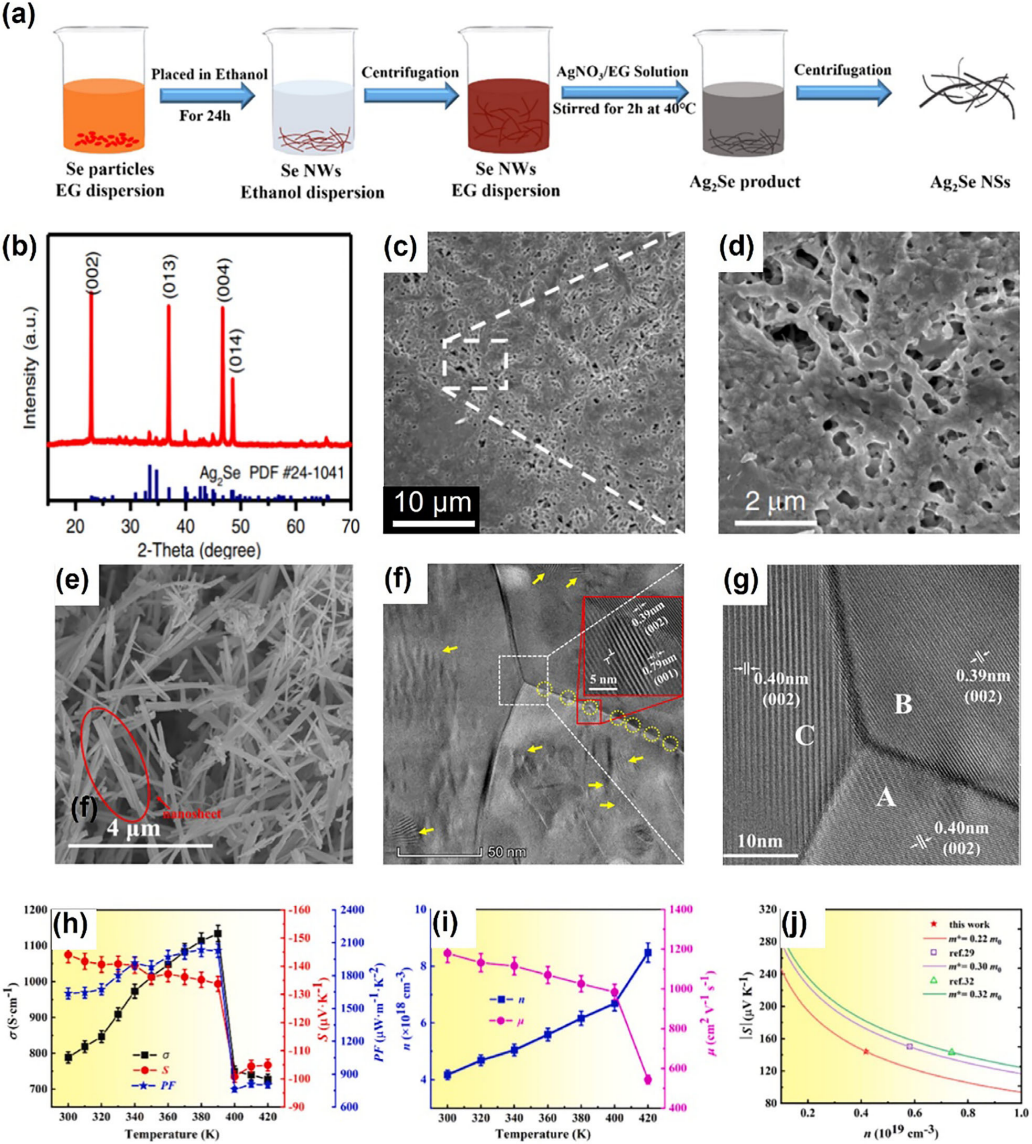
(a) Schematic illustration of the synthesis process for Ag_2_Se nanowires; (b) XRD pattern of the Ag_2_Se film; (c, d) surface FESEM images of the Ag_2_Se film at low and high magnifications, respectively; (e–g) FESEM image of Ag_2_Se nanowires and representative HRTEM images of Ag_2_Se films, highlighting the triangular grain boundaries. (a, e, f, and g) Reproduced with permission [148]. Copyright 2020, American Chemical Society. (b, c, and d) Reproduced with permission [50]. Copyright 2019, Springer Nature. (h–j) temperature‐dependent variations of *S*, *σ*, *PF*, *n*, and *µ* of the optimized Ag_2_Se film, as well as the correlation between |*S*| and *n* with estimated density‐of‐states (DOS) effective mass, reproduced with permission [150]. Copyright 2023, Elsevier.

In 2019, Ding et al. [50] prepared Ag_2_Se nanowires using a Se nanowire template and then fabricated a flexible Ag_2_Se film after vacuum‐assisted filtration and hot‐pressing (Figure 8a–d). The highly oriented crystallinity and dense structure were beneficial for enhancing its TE performance, achieving a *PF* of ∼ 987 µWm^−1^K^−2^ at 300 K [50]. Subsequently, researchers focused on Ag_2_Se‐based TE materials using the Se nanowire template method. Jiang et al. [148] further optimized the TE performance by controlling the reaction time and temperature, realizing an optimized *PF* of 1882 µWm^−1^K^−2^ at RT (reaction at 40°C for 2 h) (Figure 8e–g). Another approach involves the synthesis of Ag nanowire templates, followed by selenization to obtain Ag_2_Se nanowires. Based on this concept, Wang et al. [150] synthesized Ag_2_Se nanowires using Ag nanowires with a Se precursor at RT and then prepared Ag_2_Se‐based TE films via vacuum‐assisted filtration by combining the hot‐pressing process (Figure 8h–j). A *PF* of 1636.9 µWm^−1^K^−2^ at RT was achieved via optimizing the Ag/Se ratio.

### 3.2 Ag_2_Se Nanorod Synthesis

Ag_2_Se nanorods have a shorter aspect ratio than nanowires, and can also be prepared via conventional Se‐template‐assisted wet‐chemical routes, in which amorphous Se synthesized in solution is first converted into Se nanorods (e.g., dispersed in ethanol), followed by a reaction with Ag precursors to form Ag_2_Se nanorods while preserving the rod‐like morphology.

Based on this method, Ag_2_Se nanorods were incorporated into polymer matrices to fabricate flexible TE films. For instance, Ag_2_Se nanorods blended with polyvinylpyrrolidone (PVP) were prepared into uniform films via vacuum‐assisted filtration without any further post‐treatment [152]. Ag_2_Se/polyvinylidene fluoride (PVDF) composite films exhibited enhanced TE performance after cold‐pressing combined with an annealing process, benefiting from improved interfacial contact and film densification [153].

By adjusting the length and diameter of the nanorods, it was possible to maintain a high *σ* while further reducing the *κ*. By sequentially employing the glancing angle deposition (GLAD) technique and selenization process, Khan et al. [151] successfully prepared Ag_2_Se nanorod arrays, and a *PF* of 3229.21 µWm^−1^K^−2^ at RT was delivered. By the same GLAD technique, hierarchical zig‐zag Ag_2_Se nanorod films with different aspect ratios were further fabricated, and a *ZT* of ∼ 1.29 at 300 K was achieved, with a corresponding *PF* of ∼ 3100 µWm^−1^K^−2^ and a reduced *κ*_l_ of ∼ 0.72 Wm^−1^K^−2^ [154].

### 3.3 Ag_2_Se Nanoparticle Synthesis

Nanoparticles offer a more straightforward synthesis route, enabling scalable production [55]. For example, Ag_2_Se nanoparticles have been synthesized via an ambient aqueous solution, one‐pot reaction conducted at RT and atmospheric pressure, without the need for high‐temperature treatment, multistep processing, or organic solvents [155]. By precisely tuning reaction parameters (e.g., pH), Ag_2_Se, Ag/Ag_2_Se, and Cu‐doped Ag_2_Se nanoparticles, etc., can be selectively obtained, demonstrating good phase and composition controllability that makes them suitable for large‐scale production.

Notably, Ag_2_Se nanoparticles can be directly synthesized into printable slurries or inks through one‐step solution‐based processes. These slurries can be readily processed into continuous films using scalable techniques such as 3D printing or screen printing, enabling efficient fabrication over large areas [156]. In addition, these solution‐processable slurries offer excellent compatibility with roll‐to‐roll manufacturing strategies, making them particularly attractive for practical implementation in flexible and wearable TE devices.

**FIGURE 9:**
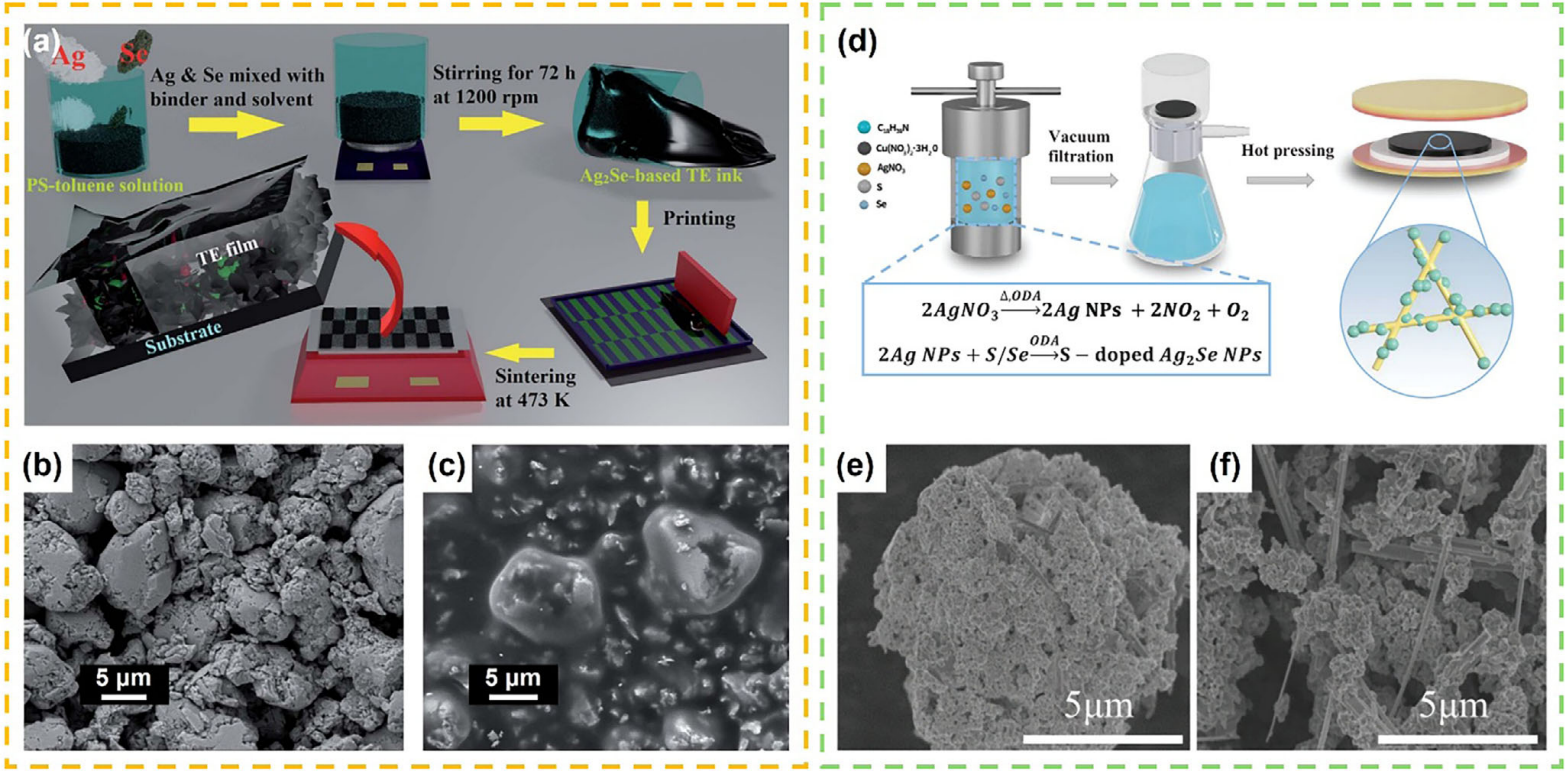
(a) Sequential steps involved in the synthesis of printable TE ink and fabrication of printed TE films. (b,c) SEM images of non‐sintered and sintered (473 K) films. (a–c) Reproduced with permission [55]. Copyright 2020, Royal Society of Chemistry. (d) Schematic of the preparation route for Ag_2_Se‐based TE nanomaterials. SEM images of (e) pristine Ag_2_Se and (f) S/Cu dual‐doped Ag_2_Se nanomaterials. (d–f) Reproduced with permission [62]. Copyright 2023, John Wiley & Sons.

For example, Mallick et al. [55] reported high‐performance Ag_2_Se‐based printed TE materials via a one‐pot synthesis, and a *ZT* of ∼ 1.03 was obtained at RT after optimizing the Ag/Se ratio and annealing process (Figure 9a–c). In this as‐prepared film, Ag_2_Se particles with different sizes below 10 µm were observed. After sintering, these particles agglomerated and lost their original morphologies. In addition to mixing Ag and Se powders, Han et al. [62] fabricated flexible S/Cu dual‐doped Ag_2_Se TE films (Figure 9d–f) using solvothermal synthesis followed by vacuum‐assisted filtration and hot‐pressing, and a *PF* of 2296.8 µWm^−1^K^−2^ at RT was achieved.

Scalability and cost‐effectiveness are also key considerations for the practical application of Ag_2_Se‐based flexible thermoelectric films and devices. The Ag_2_Se nanowires/nanorods are commonly synthesized via multi‐step and template‐assisted processes (e.g., Se template). The complex fabrication techniques and relatively high cost of Ag_2_Se nanowires/nanorods may pose challenges for large‐scale production. In contrast, nanoparticle‐based approaches, particularly those involving the one‐step synthesis of printable Ag_2_Se inks or slurries, allow direct processing via screen printing or solution 3D printing, significantly shortening the fabrication cycles and reducing costs.

## 4 Advanced Strategies for Performance Enhancement

To improve the performance of flexible Ag_2_Se‐based TE films, advanced strategies for optimizing the *PF* and *ZT* have become key areas of focus, such as adjusting nanostructuring, doping, stoichiometry, and introducing the second phases, thereby increasing their potential for applications in flexible wearable electronic devices (as summarized in Table 2). Unless otherwise specified, the TE performance metrics summarized in Table 2 are derived from experimentally measured data. Reports involving theoretical calculations or simulations were explicitly indicated.

**TABLE 2:** Measured TE performance of flexible Ag_2_Se‐based TE films via different advanced strategies.

Materials	*σ* (Scm^−1^)	*S* (µVK^−1^)	*PF* (µWm^−1^K^−2^)	*κ* (Wm^−1^K^−1^)	*ZT*	Temperature (K)
Doping						
Te‐doped Ag_2_Se [61]	990	−146.0	2110	∼0.55	1.15	300 K
Te‐doped Ag_2_Se [76]	∼1350	∼ −138	2570	∼0.73	∼1.06	303 K
Se‐doped Ag_2_S [131]	743	−81.4	492.6	∼0.57	∼0.26	300 K
S‐doped Ag_2_Se [134]	950	−150	2058			300 K
S‐doped Ag_2_Se [133]	605.2	−130.8	1035.4	∼0.65	∼0.48	300 K
Ga‐doped Ag_2_Se [135]	∼880	∼ −115	1162			300 K
Cu‐doped Ag_2_Se [164]	∼1220	∼ −145	2560	∼0.82	0.94	300 K
Cu‐doped Ag_2_Se [132]	∼1350	∼ −125	2080			300 K
Cu‐doped Ag_2_Se [136]	908	−150	2540	∼0.85	∼0.90	300 K
Cu/S‐doped Ag_2_Se [62]	1190	−138.5	2296.8			300 K
Stoichiometric ratio						
Ag_2_Se [74]	∼1760	∼ −110	2160	∼1.25	∼0.6	348 K
Ag_2_Se [137]	∼3050	∼ −88	2436	< 1.32	> 0.55	300 K
Ag_2_Se [82]	∼1400	∼ −112	1825			300 K
Ag_2_Se [162]	899	−140	1762			300 K
Ag_2_Se [77]	816	−143	1700			300 K
Ag_2_Se [177]	∼1650	∼ −122	2450.9			303 K
Ag_2_Se [178]	1091	−142	2190			300 K
Ag_2_Se [138]	840	−161.7	2205	0.61	1.10	300 K
Ag_2_Se [139]	∼1390	−149.3	3100	1.28	0.72	300 K
Ag/Ag_2_Se [140]	1549	−121	2275			300 K
Introducing second phase						
SWCNT/Ag_2_Se [111]	1656.64	−108.1	1936			300 K
SWCNT/Ag_2_Se [141]	704	−121	1030.7			300 K
MWCNT/Ag_2_Se [179]	∼241.1	∼ −145	∼510	∼1.08	∼0.18	300 K
rGO/Ag_2_Se [142]	183.37	−111.72	228.88			331 K
rGO/Ag_2_Se [39]	∼1480	∼ −158	3700	< 0.9	1.28	300 K
Ag_2_Se/carbon [143]	937	−131	1617	∼0.978	∼0.50	300 K
Ag/Ag_2_Se/graphene [144]	789.71	−144.24	1643			300 K
Ag_2_Se/CuAgSe [157]	1300	−96	1217	∼0.768	∼0.48	300 K
Ag/Ag_2_Se [64]	1040.2	∼ −92.5	889			300 K
Ag/Ag_2_Se [79]	∼3030	−116.3	> 4000	∼1.77	0.70	303 K
Cu_2_Se/Ag_2_Se [165]	1065	−126	1680	0.909	∼0.55	300 K
Ag_2_Se/Ag/CuAgSe [7]	10770	−45.5	2231.5	1.32	∼0.50	300 K
PVDF/Ag_2_Se [153]	421.9	−146.4	904.6			360 K
PEDOT:PSS/Ag_2_Se [163]	∼640	∼ −72	327.15			300 K
PEDOT/Ag_2_Se/CuAgSe [145]	∼1080	−121.8	∼1603	0.46‐0.80	0.60‐1.05	300 K
Terpineol/Ag_2_Se [54]	814	−138	1550	∼0.57	∼0.80	300 K
Ag/PVP/Ag_2_Se [110]	1286	∼ −139	2478	∼0.71	∼1.05	300 K
Ag_2_Se/Ag/PVP [146]	∼5550	−75	3119	1.09	∼0.86	300 K
Ag_2_Se/Ag/PEDOT [147]	∼5957.3	∼ −49.2	∼1442.5	∼1.32	∼0.42	300 K
Ag_2_Se/Se/PPy [53]	1064	−144	∼2240	∼0.71	∼0.94	300 K
PVP/Ag_2_Se [71]	929	−143	1910	∼0.53	∼1.10	300 K
PEI/Ag_2_Se [57]	∼1120	∼ −142	2239			300 K
PVP/Ag_2_Se/MC [90]	753.92	−147.24	1634.37	∼0.52	0.94	300 K
Ag_2_Se/MXene	1797	−109	2125	∼0.92	0.59	300 K
Nanostructuring						
Ag_2_Se/MC [63]	789.8	−128.89	1312.08			300 K
Ag_2_Se [148]	920	−143	1882	< 0.706	∼0.80	300 K
Ag_2_Se [180]	692	−130	1169			300 K
Ag_2_Se [158]	540	−215	2500			300 K
Ag_2_Se [181]	931	−151.7	2140	1.08	0.59	300 K
Ag_2_Se [182]	623	−116.2	840			300 K
Ag_2_Se [183]	1060	−121	∼1552	0.892	0.514	300 K
Ag_2_Se [72]	796	−175	∼2438	∼0.61	1.2	300 K
Ag_2_Se [50]	497	−140	987	< 0.478	∼0.60	300 K
Ag_2_Se [184]	958.9	−137.9	1825.1	∼0.80	0.68	300 K
Ag_2_Se [149]	40.8	−134.4	73.7			300 K
Ag_2_Se [150]	788.3	−144.1	1636.9			300 K

### 4.1 Nanostructuring

Nanostructuring is a common strategy for optimizing the TE performance by reducing the dimensions of the material. Nanostructured Ag_2_Se‐based films have achieved good TE properties even after being bent for different times, such as Ag_2_Se (*PF* ∼ 987 µWm^−1^K^−2^ at 300 K, *σ* retaining 93% after 1000 bends) [50], Ag_2_Se (*PF* ∼ 1884 µWm^−1^K^−2^ at 300 K, *PF* retaining 90.7% after 1000 bends) [148], and Ag_2_Se/PVDF (*PF* ∼ 904.6 µWm^−1^K^−2^ at 360 K, *σ* retaining 98.3% after 100 bends) [153].

By nanostructuring Ag_2_Se (e.g., nanowires, nanorods, or nanoparticles), the number of internal interfaces in the material can be increased, which is beneficial for reducing *κ* [140]. As Ding et al. reported [50], such nano‐crystal boundaries and microstructural defects formed during nanowire sintering enable effective phonon scattering, thereby reducing the in‐plane *κ* to below 0.48 Wm^−1^K^−1^ at 300 K, and yielding a corresponding *ZT* of ∼ 0.6. Similarly low *κ* has been widely observed in various nanostructured Ag_2_Se‐based systems, e.g., PVP/Ag_2_Se (∼ 0.53 Wm^−1^K^−1^) [71], Te‐doped Ag_2_Se (∼ 0.55 Wm^−1^K^−1^) [61], S‐doped Ag_2_Se (0.65 Wm^−1^K^−1^) [133], Ag_2_Se/CuAgSe (0.77 Wm^−1^K^−1^) [157], terpineol/Ag_2_Se (∼ 0.57 Wm^−1^K^−1^) films [54].

Additionally, in some nanostructured Ag_2_Se systems, the emergence of secondary phases and further enrichment of internal interfaces might introduce an energy‐filtering effect, resulting in an enhanced *S*. For example, Ji et al. [158] fabricated dense and flexible Ag_2_Se films via vacuum‐assisted filtration and hot‐pressing from Ag_2_Se nanowires and nanoparticles. In this process, excess Ag atoms act as effective fusing agents, promoting plastic deformation and enabling close grain contact while preserving a high grain boundary density. The resulting grain boundaries induce a pronounced energy‐filtering effect, which selectively scatters low‐energy carriers and then enhances *S* without degrading *σ*. As a result, the Ag_2_Se films exhibit an outstanding *S* of −215 µVK^−1^ and a high *PF* of ∼ 2500 µWm^−1^K^−2^ at RT [158].

### 4.2 Doping

In Ag_2_Se‐based materials, doping with metal (e.g., Cu and Sb) or non‐metal elements (e.g., S and Te) can effectively improve *σ* and/or *S* [159, 160, 161]. For instance, doping with Cu or Te can increase *n* and *µ*, thus improving the *σ* of the material [62]. Additionally, doping can also modulate *κ*_l_ by enhancing phonon scattering, which reduces *κ* and improves TE performance [61, 155]. Furthermore, doping can enhance the mechanical flexibility of a material, providing better reliability in complex working environments [132].

**FIGURE 10:**
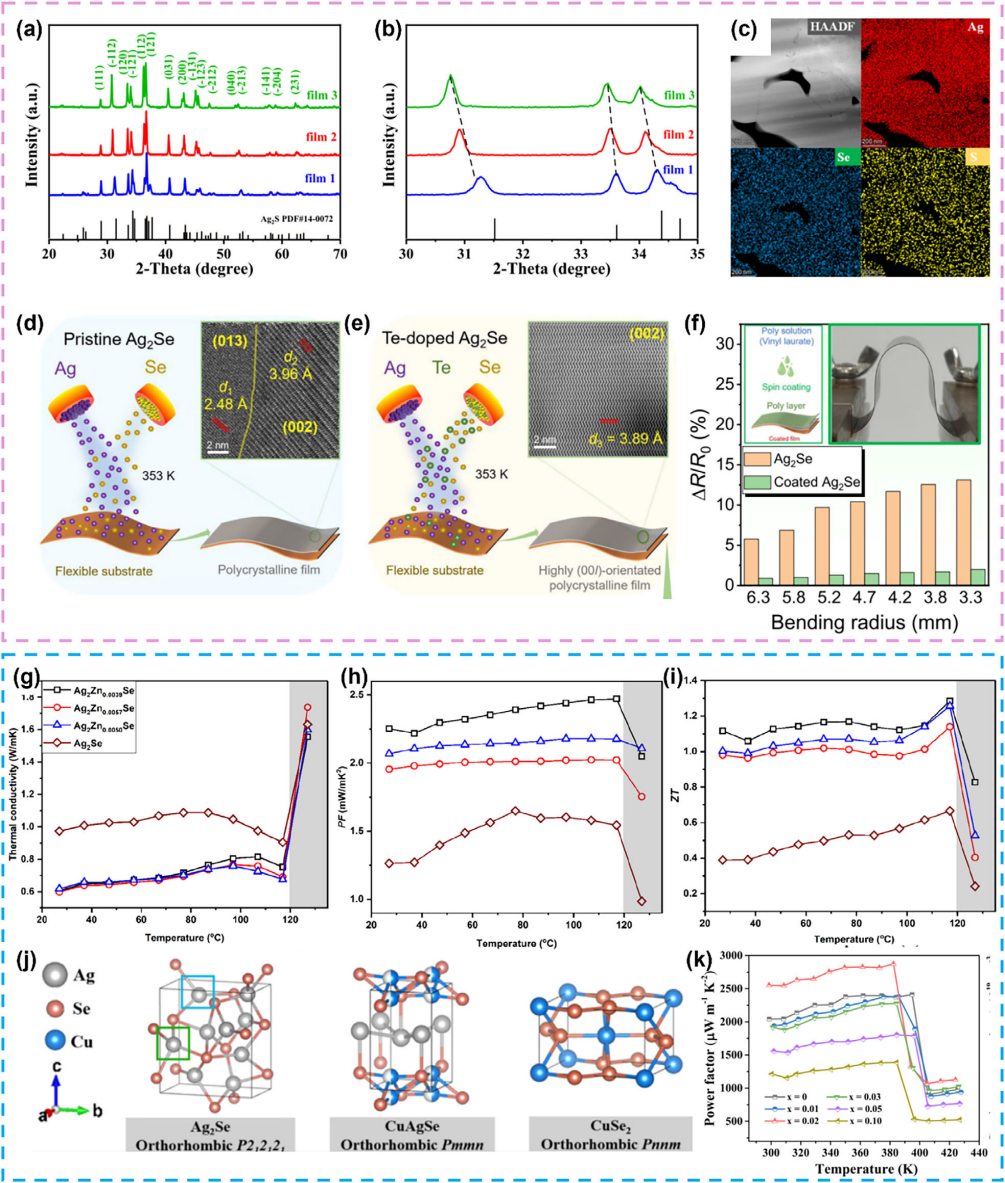
(a) XRD patterns of films with different Se/Ag_2_S ratios; (b) enlarged view of the XRD peaks at diffraction angle of 2*θ* = 30 – 35°; and (c) HAADF image for the optimized film and corresponding EDS mapping. (a‐c) Reproduced with permission [131]. Copyright 2023, American Chemical Society. Illustrations of the vacuum thermal co‐evaporation process for fabricating Ag_2_Se films (d) without and (e) with Te doping. TEM image in (d) reveals the polycrystalline nature of the pristine Ag_2_Se film, whereas for the Te‐doped Ag_2_Se film, the TEM image in (b) shows the pronounced (00*l*)‐preferred orientation. (f) Δ*R*/*R*_0_ of the optimized Te‐doped Ag_2_Se films with and without protective coating. (d‐f) Reproduced under the terms of the Creative Commons CC‐BY Creative Commons Attribution 4.0 International license (https://creativecommons.org/licenses/by/4.0) [61]. Copyright 2024, The Authors, published by Springer Nature. Temperature dependence of (g) *κ*, (h) *PF*, and (i) *ZT* for pristine Ag_2_Se and doped Ag_2_Zn_x_Se samples (x = 0.0039, 0.0050, and 0.0057, corresponding to 0.2, 0.25, and 0.3 at.% Zn doping, respectively), and the two‐phase (*β* to *α*) region is shaded in gray, reproduced with permission [69]. Copyright 2024, American Chemical Society. (j) Schematic crystal structures of Ag_2_Se, CuAgSe, and CuSe_2_; and (k) temperature‐dependent *PF* of the (Ag_1‐x_Cu_x_)_2_Se (x = 0 – 0.10) films. (j, k) Reproduced with permission [136]. Copyright 2024, American Chemical Society.

Stepwise optimization of the dopant concentration provides an effective route for fabricating films with good TE properties and excellent flexibility. Wu et al. [131] prepared flexible Ag_2_S_1‐x_Se_x_ composite films using a combined approach involving wet‐chemical synthesis, vacuum‐assisted filtration, and hot‐pressing. XRD patterns and EDS mapping confirmed the introduction of S (Figure 10a–c). With a Se/Ag_2_S molar ratio of 0.6, the film delivered a *PF* of 477.4 µWm^−1^K^−2^ at RT, reflecting improved TE performance, while its 𝜎 decreased by merely ∼ 5.4% after 2000 bends at a 4 mm radius, demonstrating excellent flexibility. Following these S‐doping strategies, flexible Ag_2_Se_1‐x_S_x_ TE films were fabricated on nylon membranes via wet‐chemical powder synthesis, vacuum‐assisted filtration, and hot pressing [133]. A clear phase evolution from Se‐substituted Ag_2_S to S‐substituted Ag_2_Se was achieved by tuning the molar ratio of Se/Ag_2_S, with the optimized film delivering a *PF* of ∼ 1035 µWm^−1^K^−2^ at 300 K and retaining ∼ 94.4% *σ* after 1000 bends. Afterward, a six‐leg FTED was assembled from the optimized films, yielding a maximum output power (*P_max_*) of ∼ 5.29 µW at a Δ*T* of 28.6 K.

Recent studies have shown that dopants can further induce crystallographic orientation in Ag_2_Se‐based films, which can contribute to improved TE performance. Yang et al. [61] developed Ag_2_Se‐based films by incorporating 3.2 at.% Te substituting for Se sites, realizing the doping‐induced orientation phenomenon, and a high *ZT* of 1.27 was obtained at 363 K (Figure 10d–f). Mechanistically, (1) Te doping significantly improves the film uniformity and promotes a pronounced (00*l*) preferred orientation by lowering the formation energy of the (00*l*) plane, as supported by the combined computational and experimental evidence. (2) This orientation regulation can enhance *µ* and *S* without sacrificing *σ*, resulting in a high *PF*. (3) The introduction of Te_Se_ point defects strengthens phonon scattering, effectively suppressing *κ*_l_. Furthermore, a corresponding FTED was assembled using the optimized film, which exhibited a maximum output power density (*PD*_max_) of 1.5 mWcm^−2^ at Δ*T* = 20 K [61]. A protective polymer composite layer was adopted to improve the mechanical flexibility [61]. Flexible Te‐doped Ag_2_Se films were fabricated via sequential solvothermal synthesis, screen printing, and spark plasma sintering (SPS) [76]. The introduction of a small amount of Te can also improve film densification, and a *PF* of 2570 µWm^−1^K^−2^ was obtained at 303 K. The films exhibit excellent mechanical robustness, retaining ∼ 96% of their initial *PF* after 1000 bends (bending radius of 5 mm) [76].

In addition to anion doping, cation doping (such as Cu, Zn, and Ga) is an effective approach. Abusa et al. [69] produced Zn‐doped Ag_2_Se and achieved a *ZT* of 1.30 at 393 K with 0.2 at.% Zn (Figure 10g–i). The *ZT* enhancement is due to Zn‐induced point defects, which scatter phonons, thereby significantly reducing *κ*. Excessive doping may destabilize the original crystal framework and induce the formation of secondary or tertiary phases. For example, Lu et al. [135] fabricated flexible Ga‐doped Ag_2_Se films by vacuum‐assisted filtration combining hot pressing, and obtained a *PF* of ∼ 1162 µWm^−1^K^−2^ at 300 K with 1 at.% Ga doping, while Ga_2_O_3_ was formed. Besides, flexible (Ag_1‐x_Cu_x_)_2_Se (x = 0 – 0.10) films were prepared using a sequential combination approach with a one‐pot synthesis, vacuum‐assisted filtration, and hot pressing (Figure 10j,k) [136]. Cu incorporation into the Ag_2_Se matrix induced the formation of additional phases, including CuAgSe and CuSe_2_. Notably, a *PF* of ∼ 2540 µWm^−1^K^−2^ (corresponding *ZT* ∼ 0.90) was obtained for the flexible (Ag_1‐x_Cu_x_)_2_Se with x = 0.02 at RT.

### 4.3 Stoichiometry

Optimizing the stoichiometric ratio is crucial for Ag_2_Se‐based TE materials because their performance is highly dependent on the precise elemental composition (Ag to Se ratio) [56]. However, excess Ag or Se can increase defects within the crystal structure, potentially affecting phonon and electron transport. Therefore, regulating the stoichiometric ratio has been widely recognized as an effective strategy for optimizing the TE performance of Ag_2_Se [140, 162].

**FIGURE 11:**
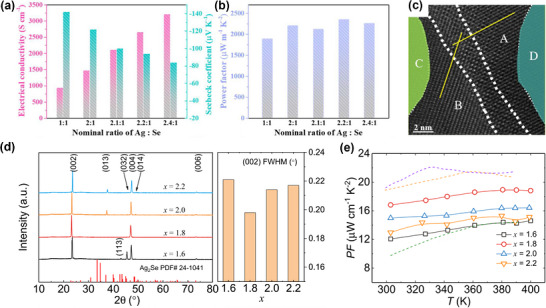
(a) *σ*, *S*, and (b) *PF* at RT of the Ag_2_Se films with different Ag/Se nominal ratios; (c) HRTEM image of an area containing a transition grain boundary. (a–c) Reproduced with permission [140]. Copyright 2024, Elsevier. (d) XRD patterns of the Ag_x_Se (x = 1.6, 1.8, 2.0, and 2.2) films, accompanied by a comparative analysis of the full width at half maximum (FWHM) for the (002) crystal plane, and (e) Temperature‐dependent *PF* (symbol lines) of Ag_x_Se (x = 1.6, 1.8, 2.0, and 2.2) films. (d,e) Reproduced with permission [77]. Copyright 2021, Elsevier.

Following this strategy, Zhang et al. [139] systematically investigated Ag_2_Se films deposited with different Ag/Se ratios. Around the RT, the *n*, *σ*, and *PF* initially increased and then decreased with tuning Ag/Se ratio from 1.92 to 2.24, whereas *µ* and *S* exhibited the opposite trend. A *PF* of 3100 µWm^−1^K^−2^ was achieved for the optimized film (Ag/Se ratio = 2.0). Liu et al. [140] prepared Ag/Ag_2_Se composite films using a sequential approach combining one‐pot synthesis, vacuum‐assisted filtration, and hot‐pressing. As the Ag/Se molar ratio increased from 1/1 to 2.4/1, *σ* increased, while *S* sharply decreased owing to the excess Ag. As a result, a *PF* of ∼ 2275 µWm^−1^K^−2^ at 300 K was achieved for the film with the Ag/Se molar ratio of 2.2/1, which can be ascribed to its unique microstructure (such as translation grain boundaries, as shown) and the synergistic effect between Ag and Ag_2_Se (Figure 11a–c) [140]. Hou et al. [77] fabricated Ag_x_Se films (x = 1.6, 1.8, 2.0, and 2.2) on a PI substrate via thermal evaporation. The Ag_1.8_Se film achieved a high *µ* due to its high orientation, crystallization, and reduced effective mass, resulting in a room‐temperature *PF* of ∼ 1700 µWm^−1^K^−2^ and a peak *PF* of ∼ 1900 µWm^−1^K^−2^ at 380 K (Figure 11d,e).

Similar to dopant‐induced orientation engineering, the crystallographic orientation can be tuned through stoichiometric control in the studies [56, 82], which is critical for enhancing the TE performance of Ag_2_Se‐based films. Zheng et al. [82] also developed flexible Ag_2_Se films through controlling the molar ratio of Ag/Se. Corresponding films with Ag/Se of 2.02 exhibited a highly (00*l*)‐textured structure and high crystallinity, resulting in a *PF* of 2160 µWm^−1^K^−2^ at 348 K. Following the same orientation‐regulation strategy, Cao et al. [56] reported a wet‐chemical selenization‐based method to prepare Ag_2_Se films with high (00*l*) and (01*l*) crystallographic orientations. Such optimized Ag_2_Se films yielded a *PF* of 3080 µWm^−1^K^−2^ at 343 K with good durability, retaining over 90% *PF* after 6‐month air exposure, as well as good flexibility, with *PF* variation below 5% after 2000 bends.

### 4.4 Introducing Secondary Phases

Introducing a second phase into Ag_2_Se‐based materials to form a composite structure is another strategy to enhance their TE performance [63]. The second‐phase material can be a metal [64], conductive polymer [163], or insulating phase [110]. Moreover, the formation of heterogeneous interfaces may induce an energy‐filtering effect [158], selectively scattering low‐energy carriers and thereby significantly improving *S* without compromising *σ*, thus offering a pathway for decoupling the interdependent TE parameters. Additionally, the selection of the second phase and its distribution within the Ag_2_Se matrix is also critical to optimizing its mechanical flexibility [71].

#### 4.4.1 Inorganic Phase

**FIGURE 12:**
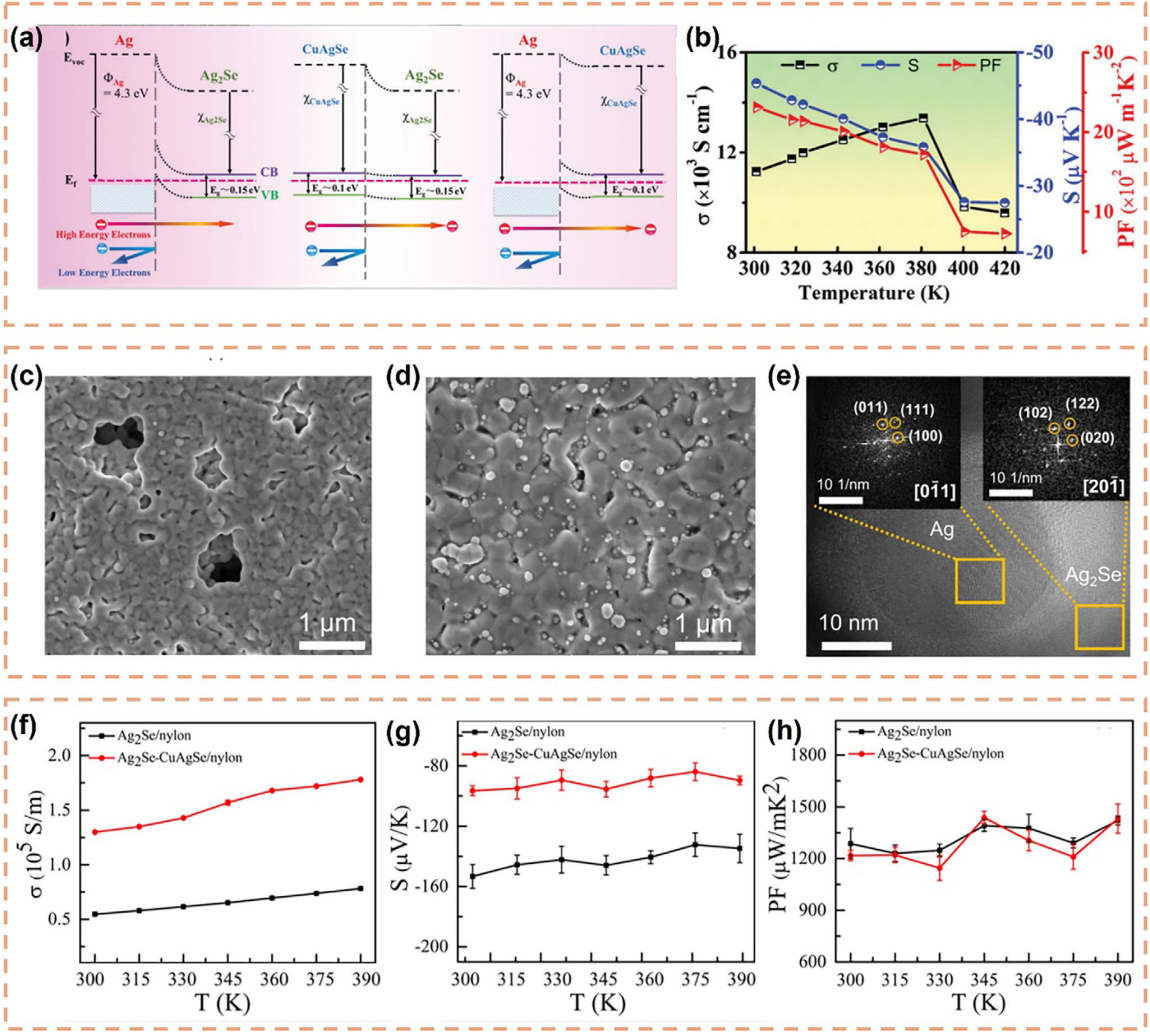
(a) Equilibrium band structures of the Ag/Ag_2_Se, CuAgSe/Ag_2_Se, and Ag/CuAgSe, and (b) variation of TE properties (*σ*, *S*, and *PF*) with temperature for the Cu1Ag4Se3 film, reproduced with permission [7]. Copyright 2020, Royal Society of Chemistry. (c, d) SEM image of Ag_2_Se film and Ag_2_Se/15%Ag composite film, and (e) High‐resolution TEM image of Ag_2_Se/15%Ag composite film with the inset showing FFT images corresponding to Ag_2_Se grain and Ag grain, reproduced under the terms of the Creative Commons CC‐BY Creative Commons Attribution 4.0 International license (https://creativecommons.org/licenses/by/4.0) [64]. Copyright 2024, The Authors, published by Springer Nature. (f) *σ*, (g) *S*, and (h) *PF* of Ag_2_Se/nylon and Ag_2_Se‐CuAgSe/nylon as a function of temperature, reproduced with permission [157]. Copyright 2023, American Chemical Society.

Inorganic secondary phases, such as metal nanoparticles [64, 137], alloys [7, 164], carbon materials [111], and MXenes [59], have been widely introduced to boost the overall TE performance of Ag_2_Se‐based composites. A flexible Ag_2_Se/Ag/CuAgSe TE film was fabricated on a nylon substrate, and a *PF* of 2231.5 µWm^−1^K^−2^ at 300 K was achieved. HAADF‐STEM images confirmed the random dispersion of numerous Ag and CuAgSe nanoparticles along the Ag_2_Se grain surfaces and boundaries. Such high TE performance was mainly related to the interfacial energy filtration effect between each composition (Figure 12a,b) [7]. Liu et al. [64] prepared Ag_2_Se/Ag films via inkjet printing technology, and the printed films exhibit a (00*l*)‐textured structure. A *PF* of 1097 µWm^−1^K^−2^ was achieved at 377 K, which could be attributed to optimized film composition (optimizing Ag content) and microstructure (promoting electron transfer via introducing Ag particles) (Figure 12c–e) [64].

Meanwhile, adding TE alloys into Ag_2_Se‐based TE materials can offer enhanced electron transport, even increase phonon scattering owing to the lattice mismatch between different phases, effectively reducing *κ*. Kumar et al. [157] fabricated flexible TE films using Ag_2_Se and CuAgSe on nylon via a chemical route, followed by cold pressing. Notably, after the introduction of the CuAgSe phase, *σ* increased significantly, whereas *S* decreased sharply, leading to no obvious change in the *PF* (Figure 12f–h). As reported by Won et al. [165], introducing trace Cu_2_Se nanoparticles (50 ppm) enhances the TE performance of freestanding flexible Ag_2_Se films, delivering a *ZT* of ∼ 0.55 around RT with a lower *κ* [165].

**FIGURE 13:**
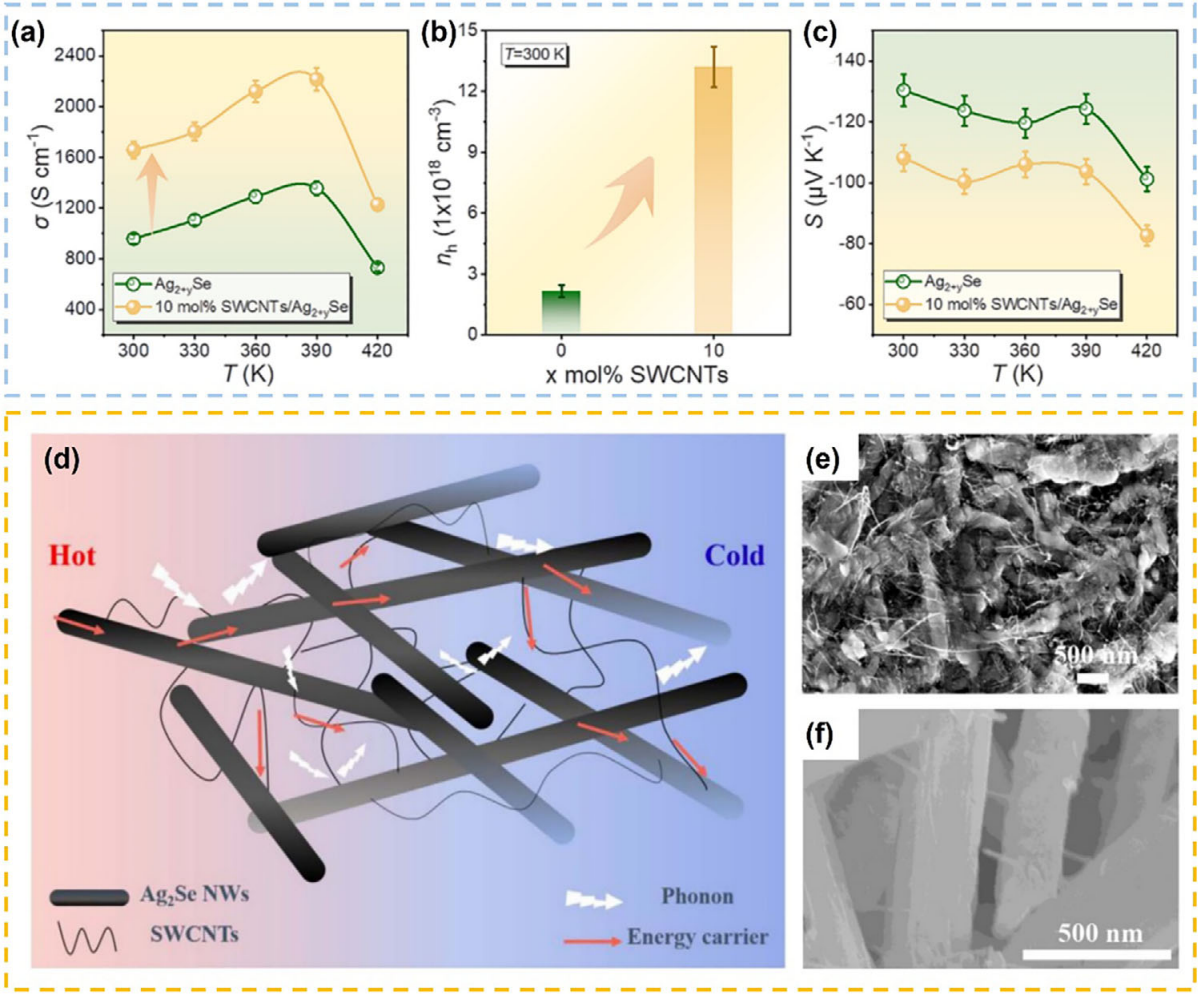
TE performance of Ag_2+y_Se film and 10 mol% SWCNTs/Ag_2+y_Se film (a) temperature‐dependent *σ*, (b) *n* at 300 K, and (c) temperature‐dependent *S*, reproduced with permission [111]. Copyright 2023, Elsevier. (d) A sketch showing the transport mechanism of the carriers and phonons in the hybrid films, and (e,f) Low and high magnification surface FESEM images of the hybrid film, reproduced with permission [141]. Copyright 2021, IOP Publishing.

Carbon‐based materials, including graphite, carbon nanotubes (CNTs), and graphene, offer high *σ*, mechanical strength, and flexibility, and therefore have been widely investigated as secondary phases for incorporation into TE materials. Hu et al. [111] reported single‐walled carbon nanotubes (SWCNTs)/Ag_2+y_Se films via an in situ grown method, yielding a *PF* of 1936 µWm^−1^K^−2^ at 300 K (Figure 13a–c). This optimized TE performance is due to the enhanced *n* and increased *m* ^*^. Geng et al. [141] prepared Ag_2_Se/SWCNTs hybrid films with a *PF* of ∼1030.7 µWm^−1^K^−2^ and good flexibility at RT, which is mainly associated with the existing network and nanobridge structure, as shown in Figure 13d–f. Extending carbon‐assisted strategies to multi‐component composite systems, Lv et al. [144] prepared Ag/Ag_2_Se/graphene (0D/1D/2D) ternary composites via vacuum‐assisted filtration and hot pressing, where the coexistence of Ag nanoparticles and Ag_2_Se nanowires effectively modulates *n*, while the Ag_2_Se/graphene interfaces simultaneously enhance the TE performance and mechanical flexibility. As a result, an optimized Ag/Ag_2_Se/graphene composite film with 0.25 wt.% graphene achieves an improved *PF* of 1643 µWm^−1^K^−2^ around RT together with good flexibility [144].

As a class of two‐dimensional transition metal carbides and nitrides, MXenes have attracted increasing attention owing to their potential in TE applications [166, 167]. For example, Ti_3_C_2_T_x_ MXene has demonstrated a *σ* of ∼ 20000 Scm^−1^ and a *PF* of ∼ 156 µWm^−1^K^−2^ [168]. Such a high *σ* of MXene can be leveraged to optimize the overall TE properties of MXene/Ag_2_Se films. Based on this concept, Park et al. [169] fabricated a Ti_3_C_2_T_x_/Ag_2_Se composite film via a wet chemical method, combining with a sintering process, and a *PF* of ∼ 933.4 µWm^−1^K^−2^ was achieved at 400 K. Qin et al. [59] fabricated composite films made of Mo_2_TiC_2_ nanosheets and Ag_2_Se nanowires via vacuum‐assisted filtration and hot‐pressing process. The cross‐dimensional structure was beneficial for carrier transport and flexibility, resulting in a *PF* of 2125 µWm^−1^K^−2^ at 300 K and only a 7% drop in *σ* after 3000 bends.

#### 4.4.2 Organic Phase

Organic second phases, particularly conducting polymers like poly(3, 4‐ethylenedioxythiophene): poly(styrenesulfonate) (PEDOT:PSS) or polypyrrole (PPy), can be introduced into Ag_2_Se to form flexible composites due to their tunable *σ*, inherently low *κ*, and good flexibility [170, 171, 172]. Corresponding composite material combines the TE advantages of Ag_2_Se, together with the TE performance, flexibility, and processability of conducting polymers, making it ideal for applications that require lightweight and bendable devices. Insulating polymers, such as PVDF and PVP, commonly serve as low‐*κ* matrices for dispersing Ag_2_Se, which can primarily improve the mechanical flexibility of Ag_2_Se‐based composites. Notably, the insulting polymer content must be carefully optimized, as excessive polymer content hinders charge transport (such as PVP/Ag_2_Se [173], bacterial cellulose/Ag_2_Se [174], or PVDF/Ag_2_Se [175]), whereas insufficient polymer content leads to limited mechanical flexibility and relatively high *κ*.

**FIGURE 14:**
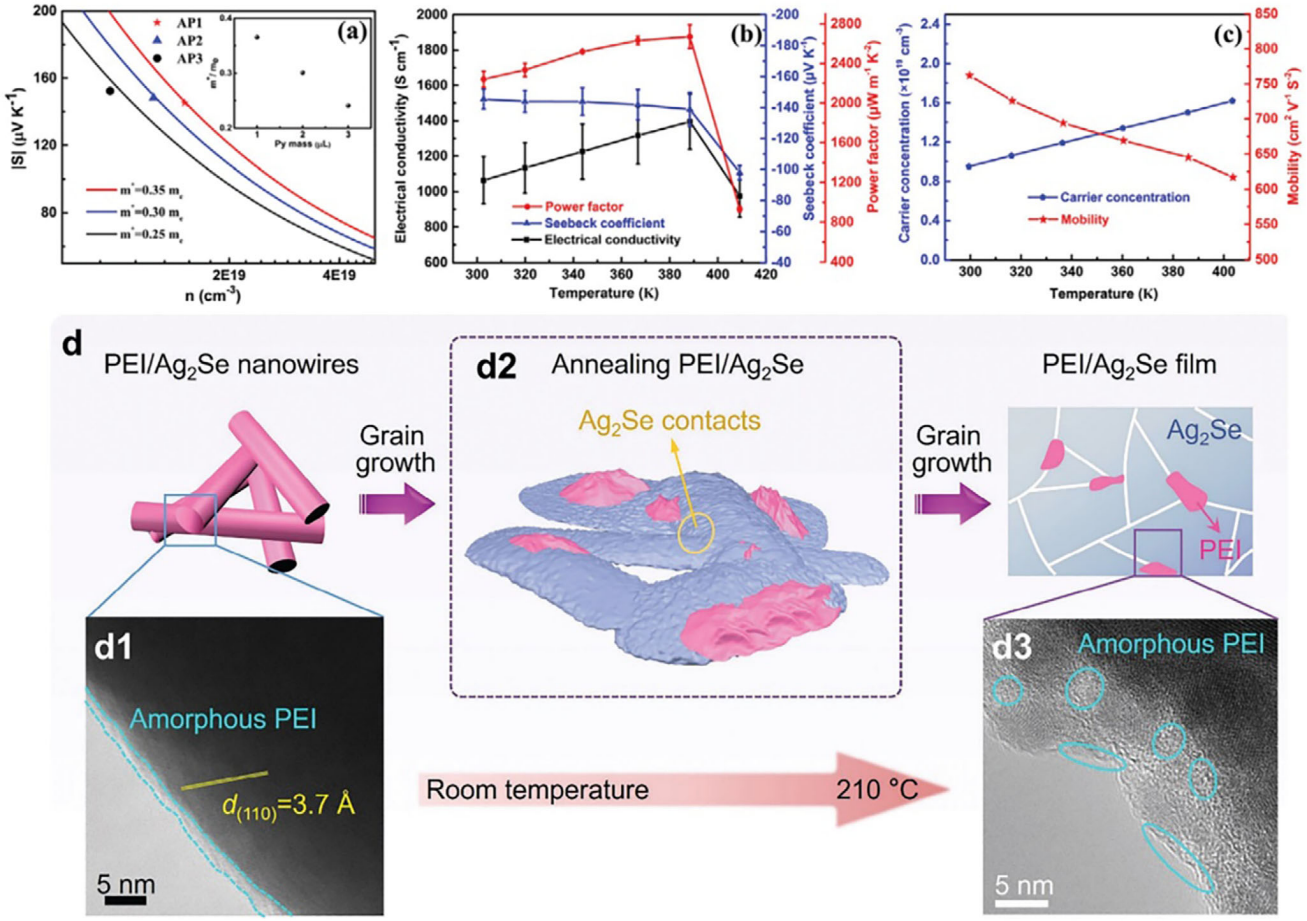
(a) Variation of *S* as a function of *n*, accompanied by calculated DOS effective mass (solid lines); the inset presents the DOS effective mass at RT. (b) *σ*, *S*, and *PF* versus temperature, and (c) *n* & *µ* of the optimized Ag_2_Se/Se/PPy TE composite film. (a, b) Reproduced with permission [53]. Copyright 2022, John Wiley & Sons. (d) Formation process of PEI inhomogeneous distribution: (d1) The high‐magnification TEM image of the 6 mol% PEI/Ag_2_Se nanowire, (d2) Schematic diagram of PEI/Ag_2_Se nanowires growth during annealing, (d3) High‐magnification TEM image of the 6 mol% PEI/Ag_2_Se composite film, reproduced with permission [57]. Copyright 2024, John Wiley & Sons.

Li et al. [53] prepared a flexible Ag_2_Se/Se/PPy composite film by sequential in situ polymerization, vacuum‐assisted filtration, and hot pressing process (Figure 14a–c). The optimal composite film achieved a *PF* of ∼ 2240 µWm^−1^K^−2^ at 300 K, which could be attributed to the synergy between crystalline Ag_2_Se, Se, and PPy. After 1000 bends at a bending radius of 4 mm, *σ* decreased by 6.5%, indicating good flexibility [53].

Hu et al. [57] applied a carrier separation strategy by compositing n‐type Ag_2_Se with polyethyleneimine (PEI) (Figure 14d1), where PEI is an electron donor. The non‐uniform distribution of PEI was found to attract minority carriers (holes) and mitigate the scattering of majority carriers through coulombic repulsion (Figure 14d2,d3), thereby yielding a high *µ* of 1551.99 cm^2^V^−1^s^−1^, and a *PF* of 2239 µWm^−1^K^−2^ at 300 K in the 6 mol% PEI/Ag_2_Se composite film. Moreover, the incorporation of PEI improved the flexibility, as evidenced by only a 6.5% increase in resistance after 1000 bends, indicating high flexibility [57].

**FIGURE 15:**
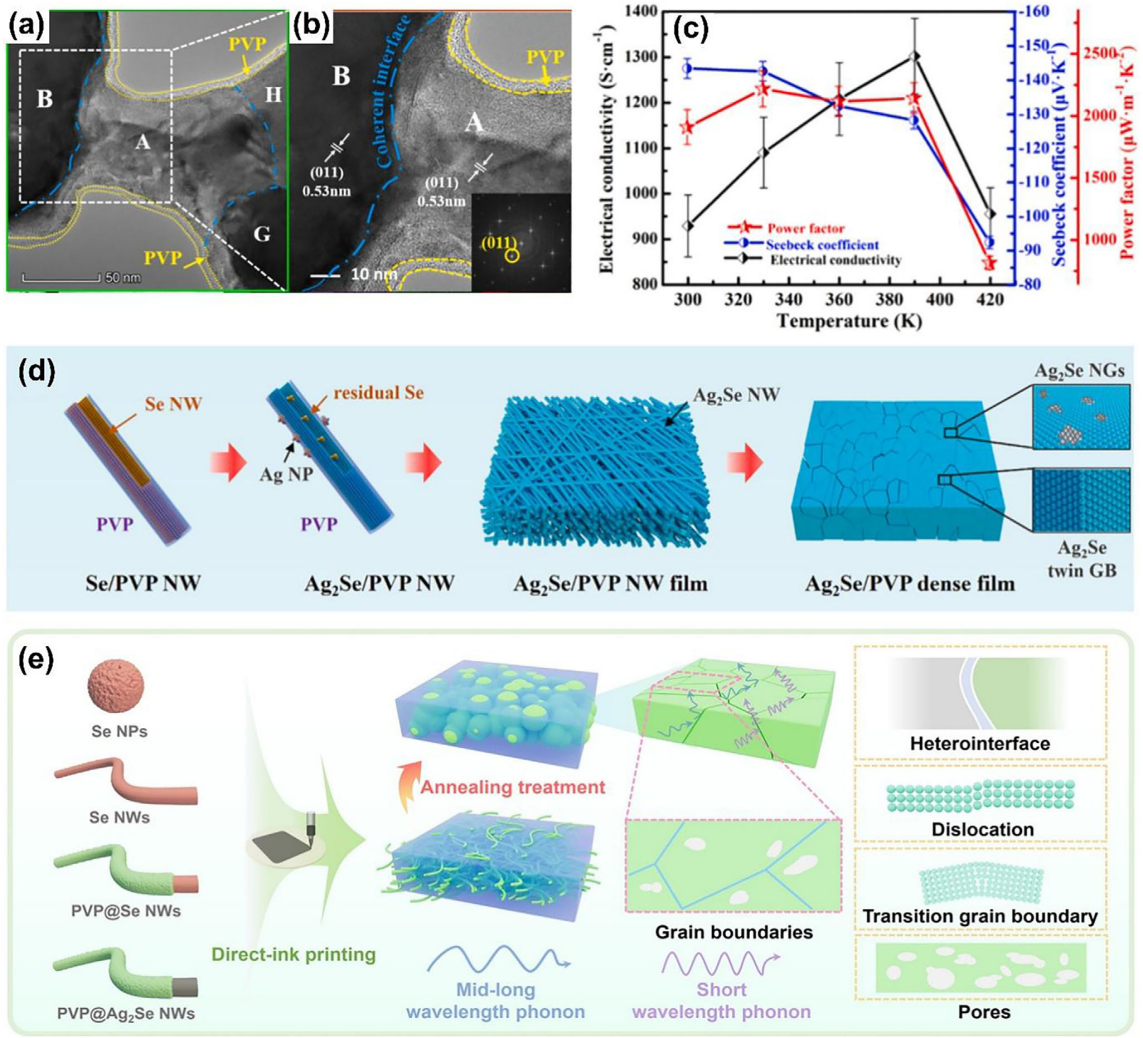
(a) Magnified view showing four Ag_2_Se grains encapsulated by PVP; (b) HRTEM image of the white box in (a) highlighting a coherent interface, with the corresponding fast Fourier transform (FFT) pattern provided in inset; (c) Temperature‐dependent *S*, *σ*, and *PF* of the PVP/Ag_2_Se composite film. (a–c) Reproduced with permission [71]. Copyright 2021, Elsevier. (d) Illustration of the growth mechanism of PVP/Ag_2_Se nanowires and the formation of PVP/Ag_2_Se composite film, reproduced with permission [110]. Copyright 2024, Elsevier. (e) Schematic diagrams illustrating the fabrication of PVP@Ag_2_Se/MC films, emphasizing the generation of intriguing phase interfaces, lattice defects, etc., reproduced with permission [90]. Copyright 2025, Springer Nature.

For example, Kumar et al. [176] prepared an Ag_2_Se film on a flexible PI substrate via the drop casting method and a *PF* of 2100 µWm^−1^K^−2^ at 405 K was obtained. Jiang et al. [71] prepared flexible PVP/Ag_2_Se composites via in situ synthesis, followed by vacuum‐assisted filtration and hot‐pressing. TEM analysis (Figure 15a,b) reveals that PVP coats the Ag_2_Se nanostructures and promotes the formation of coherent interfaces between adjacent Ag_2_Se grains, which facilitates carrier transport while suppressing interfacial scattering. Therefore, the film exhibits a *PF* and a *ZT* of ∼ 1910 µWm^−1^K^−2^ and ∼ 1.1 at 300 K, respectively (Figure 15c), with only a 5.5% decrease in *σ* after 1000 bends at a bending radius of 4 mm.

Building on these advances, Lu et al. [110] prepared PVP‐coated Ag/Ag_2_Se composite films by vacuum‐assisted filtration and hot‐pressing (Figure 15d). The optimized film achieved a high *PF* of 2478 µWm^−1^K^−2^ (corresponding *ZT* of ∼ 1.05) at 300 K, which could be ascribed to the densified Ag_2_Se grains, embedded Ag nanograins, and minimal PVP in the nanopores. Then, Zhang et al. [146] fabricated Ag_2_Se/Ag/PVP composite films using the same procedure. Owing to the dense microstructure (coherent/twin Ag_2_Se‐Ag grain boundaries) and PVP‐assisted grain binding, the optimized film delivered a high *PF* of 3119 µWm^−1^K^−2^ at RT while maintaining good flexibility (*σ*/*σ*_0_ = 96.5% after 1500 bending cycles). An FTED assembled from the optimized film achieved a *PD*_max_ of 64.8 Wm^−2^ (corresponding to a normalized power density of 704 µWm^−1^K^−2^) at a Δ*T* of 42.9 K.

Furthermore, Qin et al. [90] prepared PVP‐coated Ag_2_Se/methylcellulose (MC) composite films via scalable direct‐ink printing (Figure 15e). Notably, the heterointerfaces, pores, boundaries, and dislocations were formed in the composite films, which are beneficial for enhancing *S* and *σ*, while reducing *κ* simultaneously. Thus, a high *PF* of 2191.5 µWm^−1^K^−2^ at 400 K, and a *ZT* of ∼ 0.94 at 300 K was delivered. Meanwhile, the as‐prepared film retained over 93% of its initial *PF* after 1000 bends, demonstrating good flexibility [90].

As discussed above, many strategies (nanostructuring, doping, stoichiometry, and introducing second phases) can effectively improve the *PF* and *ZT* of Ag_2_Se‐based flexible TE materials. Despite the significant progress achieved, several intrinsic limitations and practical trade‐offs must be considered. (1) A relatively high or low Ag/Se ratio would induce the accumulation of Ag or Se ions, even may result in the formation of the second phase of Ag/Se purity. Thus, the stoichiometric ratio should be adjusted to a rational range. (2) Excessive or poorly controlled doping may introduce phase instability, increase material costs, or degrade mechanical flexibility. Especially, high doping concentrations can exacerbate lattice distortion and defect accumulation, which may negatively affect the long‐term stability. (3) Many studies on polymer‐coated Ag_2_Se‐based composite films have exhibited both high TE properties and good flexibility, and more attention should be paid to the type and content of the introduced polymers. (4) In Ag_2_Se‐based composite films, the energy filtering effect is an efficient approach for improving *S* while maintaining *σ*. (5) In practical large‐scale production, especially prolonged or complex experiments, as well as rare, expensive, and toxic components, should be carefully considered. Considering these limitations, a combined strategy should be applied for balancing TE performance with stability, cost, and scalability.

## 5 Flexible Ag_2_Se‐Based Films via Various Methods

In the fabrication of flexible Ag_2_Se‐based TE films, selecting an appropriate processing method is crucial for enhancing the TE performance and flexibility, which are important for meeting the demands of wearable electronic devices. Several commonly used fabrication methods include vacuum‐assisted filtration [7, 50, 53, 57, 59, 62, 71, 110, 111, 133, 135, 136, 137, 140, 141, 147, 148, 157, 169, 179, 185, 186, 187, 188, 189], screen printing [54, 55, 63, 76, 134, 143, 156, 173, 190, 191, 192], magnetron sputtering [72, 132, 139, 164, 193], thermal evaporation [61, 74, 77, 82, 162, 178, 181, 194, 195], drop casting [163, 165, 175, 176, 177, 182, 183, 196], and other innovative techniques [58, 64, 73, 90, 138, 151, 153, 174, 180, 197, 198]. Each method has its own advantages and challenges.

**FIGURE 16:**
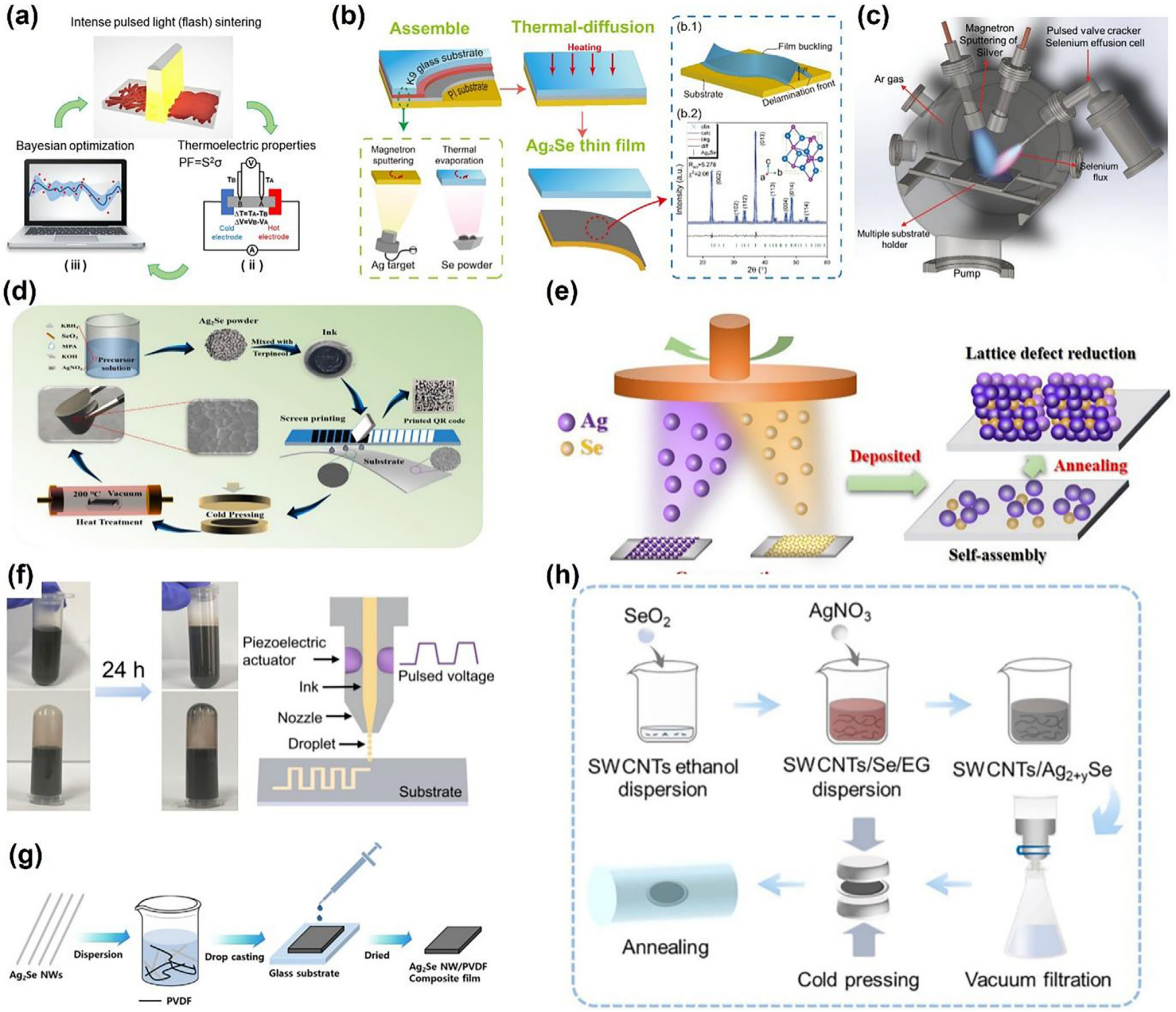
Flexible Ag_2_Se‐based TE films fabricated via different methods [54, 64, 72, 82, 111, 138, 162, 175]. (a) Reproduced with permission [138]. Copyright 2022, Royal Society of Chemistry. (b) Reproduced with permission [82]. Copyright 2023, American Chemical Society. (c) Reproduced with permission [72]. Copyright 2017, John Wiley & Sons. (d) Reproduced with permission [54]. Copyright 2024, Elsevier. (e) Reproduced with permission [162]. Copyright 2021, Elsevier. (f) Reproduced under the terms of the Creative Commons CC‐BY Creative Commons Attribution 4.0 International license (https://creativecommons.org/licenses/by/4.0) [64]. Copyright 2024, The Authors, published by Springer Nature. (g) Reproduced with permission [175]. Copyright 2021, Elsevier. (h) Reproduced with permission [111]. Copyright 2022, Elsevier.

Figure 16 presents typical fabrication strategies for flexible Ag_2_Se‐based TE films. Vapor‐ and solid‐phase processes (e.g., thermal evaporation and magnetron sputtering) lead to dense films with some degree of crystallographic orientation, which is favorable for carrier transport. While solution‐based procedures (e.g., vacuum‐assisted filtration and drop casting) and printing‐related methods (e.g., screen printing and 3D printing) provide better process flexibility but may introduce higher porosity and then result in lower electrical transport. Overall, the fabrication methods should be selected according to specific application requirements.

### 5.1 Vacuum‐Assisted Filtration

Vacuum‐assisted filtration is a method in which a dispersion of nanomaterials is filtered under vacuum to form a film that is particularly suitable for producing flexible films [199, 200, 201]. This method offers good control over the film thickness and surface smoothness while maintaining material flexibility [202]. Zhao et al. [152] prepared flexible Ag_2_Se/PVP composite films with different Ag_2_Se content by solution mixing and vacuum‐assisted filtration without post‐treatment. The TE performance could be tuned by the Ag_2_Se content, yielding a *PF* of 16.18 µWm^−1^K^−2^ at 320 K with 0.15 wt.% PVP.

**FIGURE 17:**
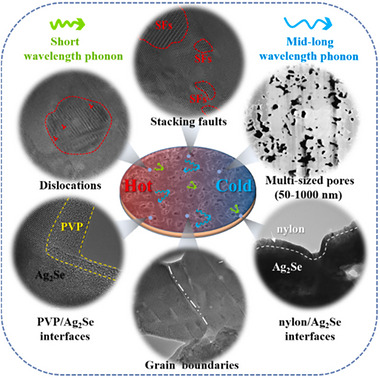
A schematic representation depicting the phonon scattering mechanisms in the PVP/Ag_2_Se film, reproduced with permission [71]. Copyright 2020, Elsevier.

The TE performance can be further enhanced by several post‐treatment methods, such as cold pressing, cold pressing combined with annealing, or hot pressing. Ding et al. [50] fabricated a flexible Ag_2_Se film on a nylon membrane via vacuum‐assisted filtration followed by hot pressing (Figure 8a–d). The highly oriented crystallinity and dense structure were beneficial for enhancing its TE performance, achieving a *PF* of ∼ 987 µWm^−1^K^−2^ at 300 K. Based on such fabrication and post‐treatment methods, Jiang et al. [71] prepared a PVP/Ag_2_Se composite film, and a *ZT* of ∼ 1.1 at 300 K was achieved. The high *ZT* could be ascribed to low *κ* resulting from the phonon scattering mechanisms in the film (Figure 17). Li et al. [137] fabricated an Ag/Ag_2_Se TE film using microwave‐assisted synthesis, vacuum filtration, and a hot‐pressed process. By optimizing the Ag/Se ratio, a *PF* of 2436 µWm^−1^K^−2^ at RT was achieved. Hu et al. [57] prepared a PEI/Ag_2_Se composite film via vacuum‐assisted filtration and cold pressing, combining an annealing process, and an optimized *PF* of 2239 µWm^−1^K^−2^ at 300 K for a 6 mol% PEI/Ag_2_Se composite film.

Notably, vacuum‐filtered films are normally deposited onto flexible substrates (e.g., Nylon, PVDF) and possess micrometer‐scale thicknesses, endowing them with excellent mechanical flexibility. Wang et al. [147] prepared Ag_2_Se/Ag/PEDOT composite films via vacuum‐assisted filtration and hot pressing. The optimized film achieved a *PF* of ∼ 1442.5 µWm^−1^K^−2^, ultrahigh *σ* of ∼ 5957.3 Scm^−1^, and good flexibility, with only a 5.5% decrease in *σ* after 1000 bending cycles. Such flexibility could be ascribed to the porous Ag_2_Se nanograin network, intrinsic flexibility of substrate, and the strong bonding interactions between the Ag_2_Se and substrate, as evidenced by several other representative studies, Ag_2_Se/Nylon (*σ*/*σ*_0_ = 93% after 1000 bends) [50], Ag/Ag_2_Se/Nylon (*σ*/*σ*_0_ = 93.3% after 1000 bends) [185], and SWCNTs/Ag_2_Se/Nylon (*σ*/*σ*_0_ = ∼ 95% after 1000 bends) [111].

### 5.2 Screen Printing

Screen printing is a widely used technique in electronics manufacturing, in which Ag_2_Se‐based paste is printed onto a substrate to form a film through a mesh screen [54]. It is low‐cost and well‐suited for large‐area production, providing effective control over the film thickness and shape [46, 203, 204].

Liu et al. [173] developed an Ag_2_Se/PVP film via screen printing, and the optimized film achieved a *PF* = 4.3 µWm^−1^K^−2^ and *σ*/*σ*_0_ = 81% after 1500 bending cycles. Zhang et al. [54] reported a flexible, self‐healing Ag_2_Se/terpineol composite film on nylon via a one‐pot synthesis, screen printing, and annealing process. The dense microstructure of the film, with Ag_2_Se grains and minimal terpineol at the boundaries, resulted in a *PF* of 1550 µWm^−1^K^−2^ (*ZT* ∼ 0.8) at RT, along with good flexibility and self‐healing properties. Xiao et al. [63] prepared Ag_2_Se/MC composite films via screen printing, cold pressing combined with an annealing process, and a *PF* of 1312.08 µWm^−1^K^−2^ was obtained at RT, with a *σ* and *S* of 789.8 Scm^−1^ and −128.89 µVK^−1^.

**FIGURE 18:**
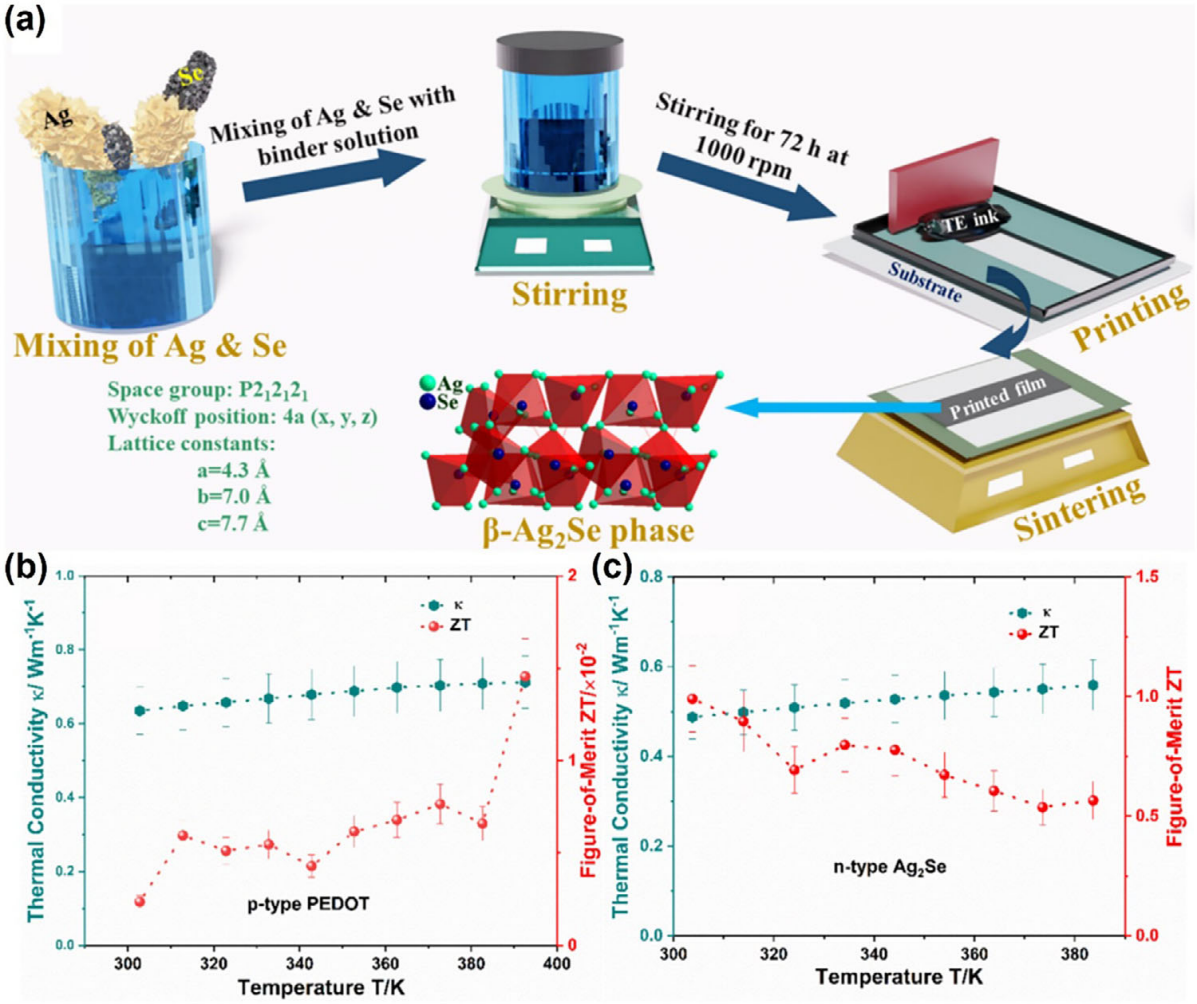
(a) Schematic representation of the fabrication process for printable Ag_2_Se‐based films. In‐plane *κ* and *ZT* versus temperature of (b) the p‐type PEDOT film and (c) the n‐type Ag_2_Se film. (a–c) Reproduced with permission [156]. Copyright 2020, American Chemical Society.

By extending screen printing to carbon‐based composite systems, Zhang et al. [143] prepared flexible Ag_2_Se/carbon nanocomposite films on PI substrates, delivering a *PF* of 1617 µWm^−1^K^−2^ at RT together with good flexibility. The enhanced TE performance was attributed to the (00*l*)‐oriented Ag_2_Se grains with coherent boundaries and a small amount of amorphous carbon [143]. The corresponding FTED assembled from the optimized films achieved a *PD*_max_ of 29.1 Wm^−2^ at Δ*T* = 35.4 K. Moreover, Mallick et al. [156] developed an Ag_2_Se‐based TE ink via a one‐step slurry formulation combined with screen printing, and a *ZT* of ∼ 1 at RT was ultimately achieved (Figure 18). This strategy offers high design freedom and shortens experimental cycles, demonstrating that one‐step printable inks coupled with screen printing can deliver competitive TE performance while enabling efficient and scalable fabrication for flexible Ag_2_Se‐based TE materials [156].

### 5.3 Magnetron Sputtering

Magnetron sputtering, a physical vapor deposition technique, enables precise control over nanostructure, composition, and properties [139, 205]. This technique allows the fabrication of highly pure and uniform films, and it is suitable for a variety of substrates. Films prepared by this technique typically exhibit high TE performance and strong adhesion, rendering magnetron sputtering ideal for high‐performance TE devices [72]. However, it requires complex equipment and high‐vacuum conditions, leading to high costs [206, 207].

For example, the Ag_2_Se film (< 500 nm thick), deposited on PI substrates via magnetron sputtering, achieved a *PF* of 526.86 µWm^−1^K^−2^ after annealing treatment [193]. Taborda et al. [72] reported an Ag_2_Se film grown by pulsed hybrid reactive magnetron sputtering, and a *PF* of 2440 µWm^−1^K^−2^ and a corresponding *ZT* of 1.2 at RT were obtained.

**FIGURE 19:**
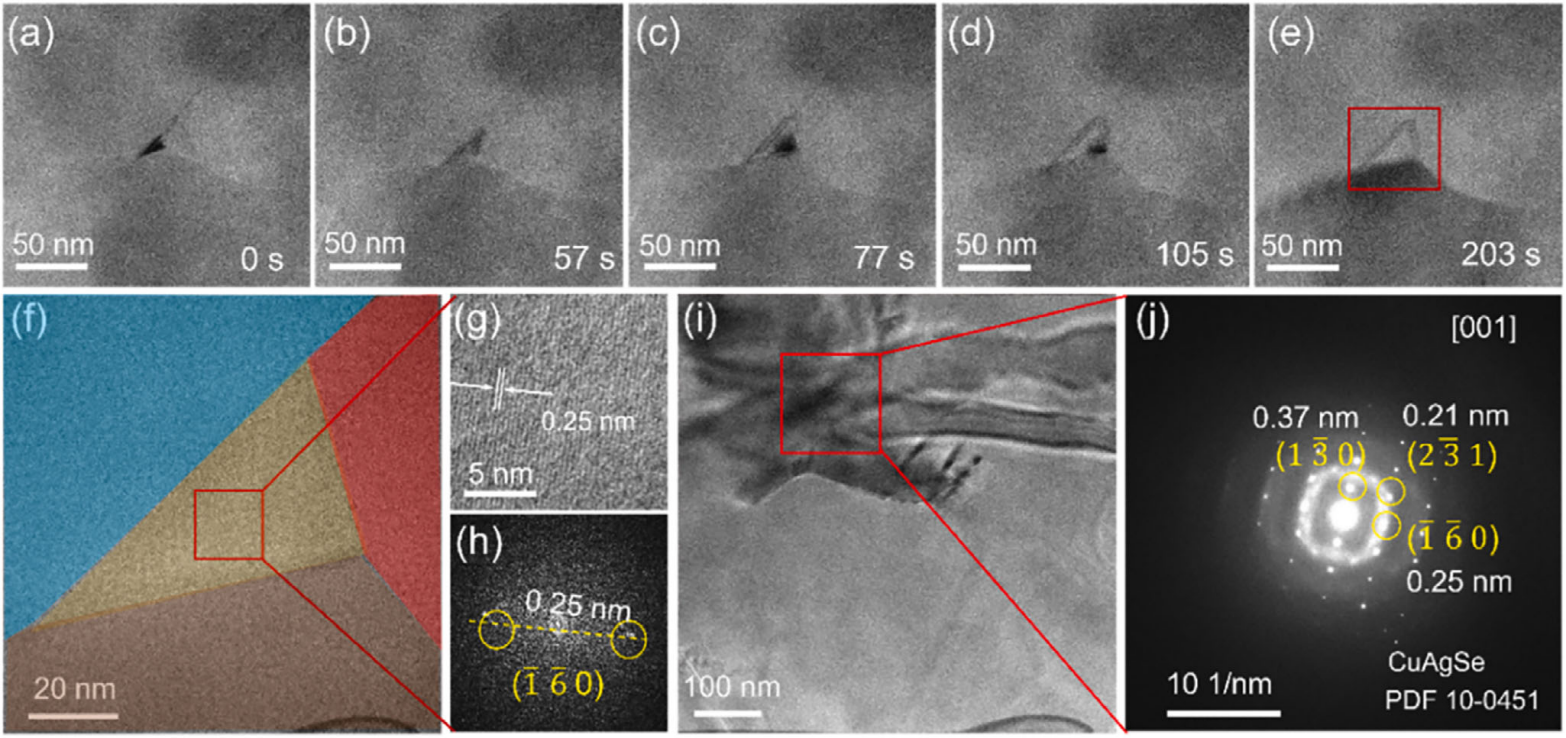
(a–e) In situ TEM images captured at different heating durations at 200°C for the Ag_2_Se/Cu_1.8_Se flexible film. (f) Magnified TEM image of the red square region in (e). (g) HRTEM image showing lattice fringes of the crystallized region, and (h) selected area electron diffraction (SAED) pattern of the same region confirming its crystalline structure. (i) Low‐magnification TEM image after thermal treatment, displaying the overall microstructural evolution. (j) SAED pattern of the red‐framed area in (i). (a‐j) Reproduced with permission [164]. Copyright 2023, Elsevier.

Hou et al. [164] prepared a flexible Ag_2_Se/CuAgSe composite film using magnetron sputtering in which Ag_2_Se and Cu_1.8_Se served as targets (the sample P14 was prepared with the Ag_2_Se target operated at 100 W and the Cu_1.8_Se target at 140 W), achieving a *PF* of ∼ 2700  µWm^−1^K^−2^ at 380 K. The in situ TEM images observed in Figure 19 provide direct evidence for the structural evolution of the Ag_2_Se/CuAgSe flexible film. Upon heating at 200°C, the amorphous matrix gradually developed crystalline nuclear, which expanded with increasing heating duration (Figure 19a–e). The enlarged TEM image of the red square region (Figure 19f) confirms the presence of multiple grains separated by clear boundaries. HRTEM analysis and SAED pattern (Figure 19g,h) reveal the high‐orientation Ag_2_Se along ($\bar{1}$ $\bar{6}\;0$). The TEM images in Figure 19i and the corresponding SAED pattern (Figure 19j) demonstrate the existence of CuAgSe phase. Therefore, thermal treatment can be an effective method to optimize the TE properties of the Ag_2_Se‐based films fabricated using magnetron sputtering.

### 5.4 Thermal Evaporation

Thermal evaporation is a technique in which a material is heated until it evaporates and condenses onto a substrate to form a film. In this process, Ag_2_Se material is evaporated by a heat source and then deposited onto a flexible substrate [61]. This method can prepare film with high uniformity, strong adhesion, as well as tunable thickness and composition [208, 209]. However, this process is also time‐consuming and requires specialized equipment.

Apart from the already‐mentioned Ag_x_Se (x = 1.6, 1.8, 2.0, and 2.2) films on PI prepared via thermal evaporation [77], Zhang et al. [82] fabricated a flexible Ag_2_Se film on a PI substrate using a thermal evaporation process. The as‐prepared films preferentially grow along the (013) orientation, which is beneficial for enhancing *S*. The optimal film achieved a *PF* of 1825 µWm^−1^K^−2^ at RT and 2170 µWm^−1^K^−2^ at 393 K. Niu et al. [195] reported Ag_2_Se TE films via an in situ growth method using co‐evaporation deposition, and a *PF* of 1534 µWm^−1^K^−2^ was achieved.

**FIGURE 20:**
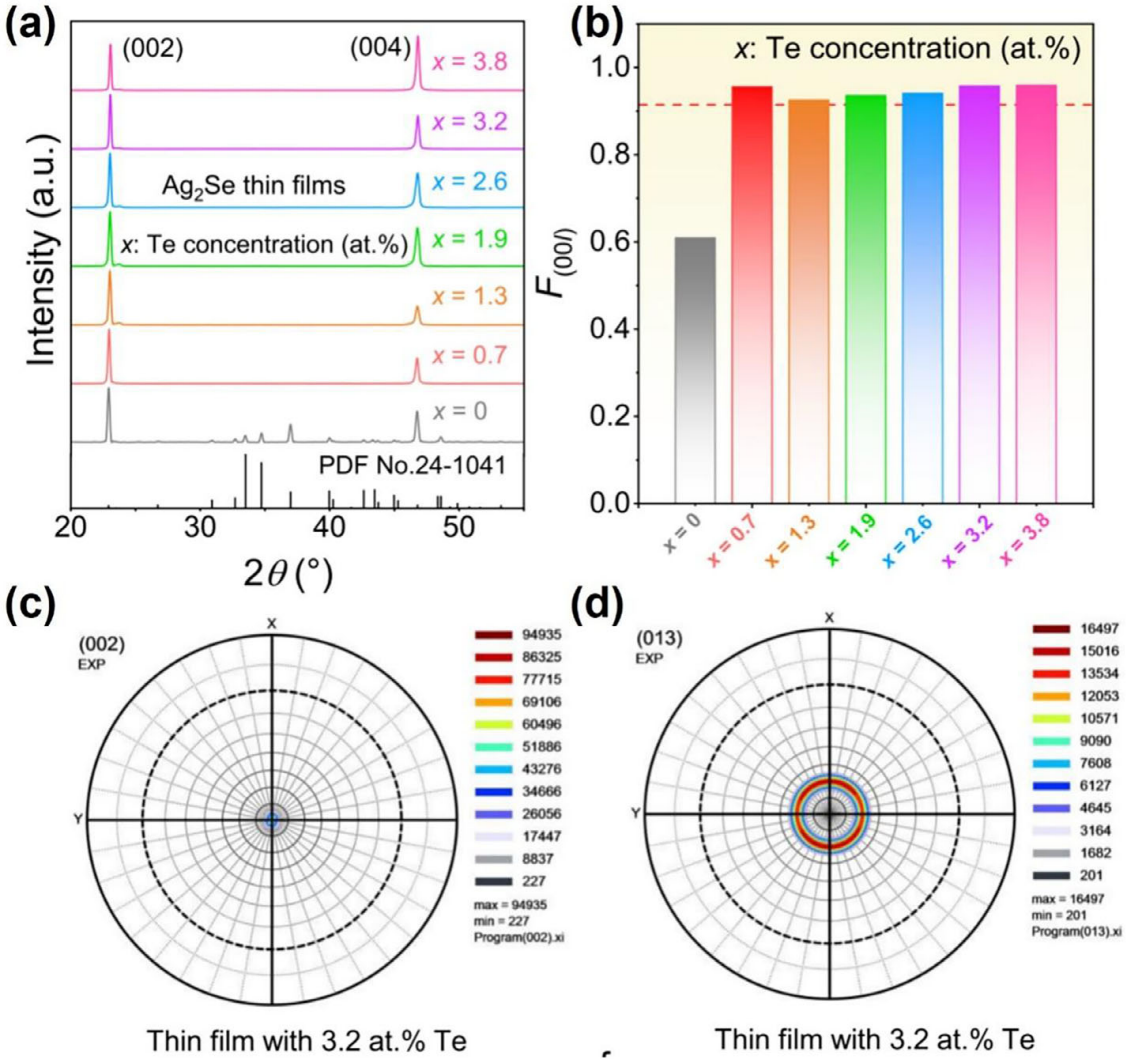
(a) XRD patterns of Ag_2_Se films with Te concentrations ranging from 0 to 3.8 at.%. (b) Calculated orientation factors (*F*) along the (00*l*) axis, suggesting enhanced (00𝑙)‐texturing with increasing Te incorporation. (c,d) Pole figures of the 3.2 at.% Te‐substituted Ag_2_Se film along the (002) and (013) orientations. (a–d) Reproduced under the terms of the Creative Commons CC‐BY Creative Commons Attribution 4.0 International license (https://creativecommons.org/licenses/by/4.0) [61]. Copyright 2024, The Authors, published by Springer Nature.

Recently, Yang et al. [61] fabricated Te‐doped Ag_2_Se thin films using a vacuum thermal co‐evaporation method, and a maximum *ZT* of 1.27 was achieved with 3.2 at.% Te doping on Se sites at 363 K (Figure 20). The diffraction patterns reveal a progressive enhancement of the (00*l*) orientations, particularly the (004) peak, as the Te concentration increases from 0 to 3.8 at.% (Figure 20a). The calculated orientation factor (*F*) along (00*l*) (Figure 20b) increases steadily with Te content, reaching a maximum at ∼ 3.2‐3.8 at.% Te. The pole figures along the (002) and (013) directions (Figure 20c,d) for the 3.2 at.% Te‐doped film exhibit sharp and symmetric patterns, respectively, consistent with the strong texturing along the *c*‐axis [61].

Zhang et al. [181] prepared (201)‐textured Ag_2_Se films using a thermal evaporation process with Se precursor control, and a *PF* of 2140 µWm^−1^K^−2^ at 300 K was delivered. Both experimental and theoretical analyses indicate that this preferred orientation affords enhanced *µ* and *S*, while the inherently low *κ* of Ag_2_Se is further reduced by nanopores. Consequently, an optimal *ZT* of 0.73 at 363 K was achieved, highlighting the potential of orientation control in optimizing the TE performance of Ag_2_Se films [181].

### 5.5 Drop Casting

Drop casting is a simple method in which dispersions are dropped onto a substrate, and the solvent is allowed to evaporate, thus forming a film [210, 211]. Owing to its high deposition rate and easy scalability, drop casting is widely used for fabricating TE films [199, 212]. The film thickness can be tuned by modifying the process parameters [182]. The mold determines the shape of the composite TE films, which is also a key limitation for this method [199].

Park et al. [175] fabricated Ag_2_Se nanowire/PVDF composite films on Nylon substrate via drop casting Ag_2_Se with various content (50–80 wt.%). The optimized sample with 70 wt.% Ag_2_Se showed good flexibility and a *PF* of 180.6 µWm^−1^K^−2^ at 400 K. Kumar et al. [176] prepared an Ag_2_Se film on a flexible PI substrate via the drop casting method, and the obtained film exhibited a *PF* of 1400 µWm^−1^K^−2^ at 300 K, with no obvious degradation after 1500 bending cycles.

**FIGURE 21:**
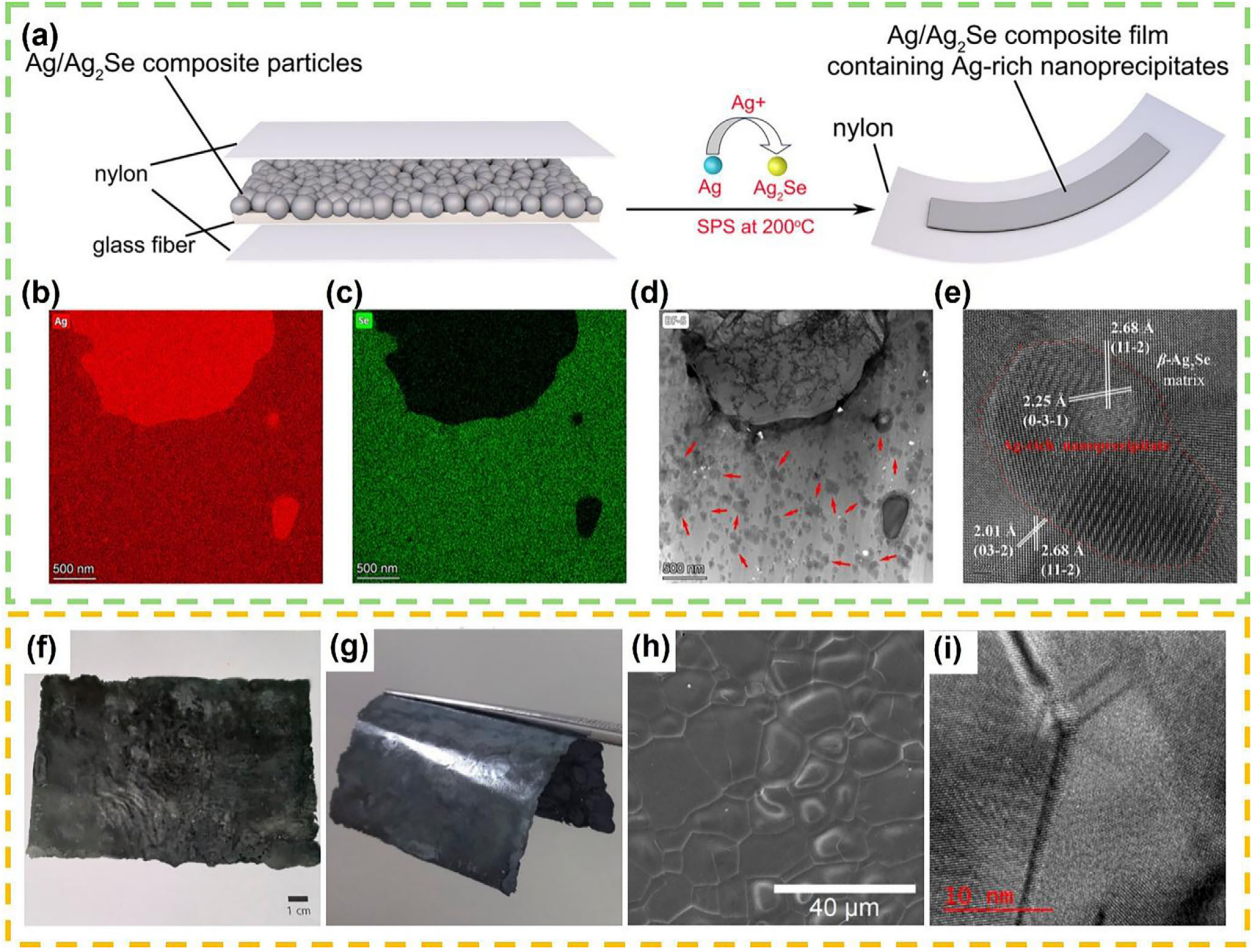
(a) Schematic diagram illustrating the fabrication of nylon‐supported flexible composite films. (b, c) EDS elemental maps of Ag and Se, respectively, and (d) bright‐field TEM image of the Ag_2.3_Se‐SPS (200°C, 5 min) film, in which typical Ag‐rich nanoprecipitates are highlighted by red arrows. (e) HRTEM image showing a coherent interface (marked by a red dotted circle) between an Ag‐rich nanoprecipitate and the *β*‐Ag_2_Se matrix. The lattice spacings marked by white lines correspond to the crystallographic planes of orthorhombic *β*‐Ag_2_Se. (a–e) Reproduced with permission [79]. Copyright 2025, Springer Nature. (f, g) Photos of flexible, free‐standing Ag_2_Se films, prepared by drop casting. (h) SEM and (i) TEM images of the free‐standing Ag_2_Se film. (f–i) Reproduced with permission [183]. Copyright 2023, American Chemical Society.

Gao et al. [79] fabricated flexible Ag/Ag_2_Se composite films via sequential drop casting on a piece of glass‐fiber sheet and SPS process (Figure 21a). Ag‐rich nanoprecipitates were formed during SPS process due to the migration and precipitation of Ag ions (Figure 21b–e). Such Ag‐rich nanoprecipitates significantly enhance *n* and DOS effective mass, resulting in an unprecedented *PF* exceeding 4000 µWm^−1^K^−2^ at 303 K. A FTED assembled from five optimized films delivered a normalized output power density of 1089 µWm^−2^K^−2^ at Δ*T* = 26.0 K [79].

Lee et al. [183] prepared free‐standing Ag_2_Se TE films via drop casting and achieved a *ZT* of 0.514 at RT. As shown in the photographs (Figure 21f,g), the films exhibited good mechanical flexibility and could be bent or folded without fracture. The SEM image (Figure 21h) reveals the dense surface morphology, and the TEM image (Figure 21i) indicates the well‐developed crystallinity of the Ag_2_Se phase. These structural features make the free‐standing Ag_2_Se films highly promising candidates for integration into FTEDs and wearable energy conversion systems [183].

### 5.6 Other Techniques for Fabricating Ag_2_Se‐based TE Films

In addition to the afore‐mentioned traditional film fabrication methods, other techniques such as inkjet printing [64], 3D printing [90, 153, 198], electrodeposition [180], and the GLAD technique [151] have also been used to fabricate flexible Ag_2_Se‐based TE films.

Inkjet printing enables the fabrication of high‐precision patterned films with complex shapes. Liu et al. [64] prepared Ag_2_Se‐based TE films with (00*l*) texture via such inkjet printing technology, and a *PF* of 1097 µW^−1^K^−2^ at 377 K was obtained, enabling corresponding FTEDs with a normalized power output (2 µWK^−2^cm^−2^).

**FIGURE 22:**
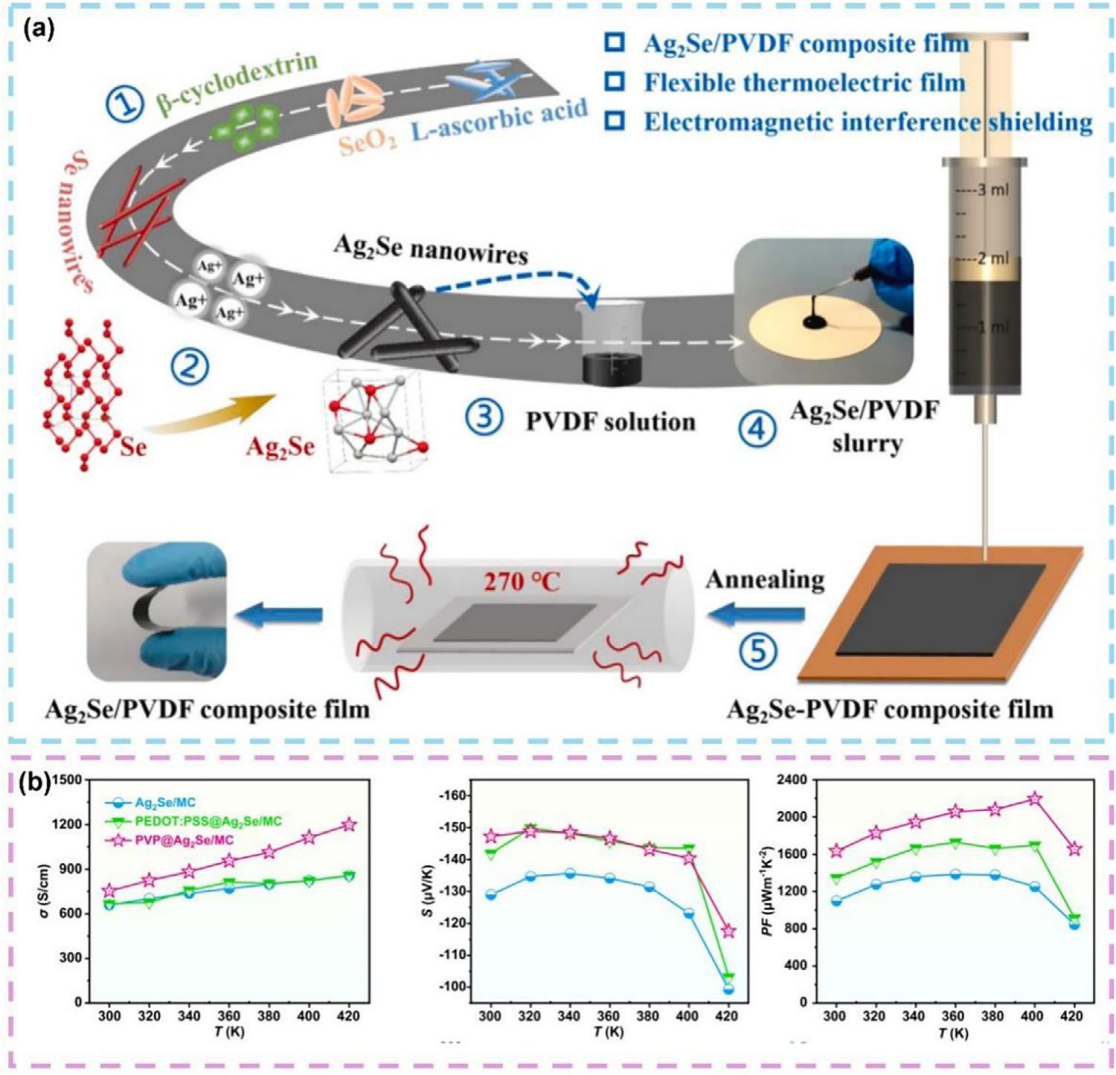
(a) Schematic illustration of the solution 3D printing technology and annealing process used to fabricate Ag_2_Se/PVDF composite films, reproduced with permission [153]. Copyright 2024, Elsevier. (b) Temperature‐dependent TE properties (300–420K) for the Ag_2_Se/MC, PEDOT:PSS@Ag_2_Se/MC, and PVP@Ag_2_Se/MC films, reproduced with permission [90]. Copyright 2025, Springer Nature.

Through solution 3D printing technology followed by an annealing process, Liu et al. [153] developed bi‐functional flexible Ag_2_Se/PVDF composite films. At an Ag_2_Se content of 85 wt.%, the film delivered a *PF* of 904.6 µWm^−1^K^−2^ at 360 K (Figure 22a), while simultaneously achieving an electromagnetic interference shielding effectiveness value of 56.1 dB at a thickness of 52 µm. Using the same solution‐based 3D printing, Qin et al. [90] prepared PVP@Ag_2_Se/MC composite film, and a high *PF* of 2191.5 µWm^−1^K^−2^ at 400 K was obtained (Figure 22b). Building upon this material‐level advancement, the direct printing of FTEDs using the solution 3D printing technology was further demonstrated. This approach effectively bypasses conventional fabrication steps (such as cutting, soldering, and packaging), thereby significantly improving device fabrication efficiency, as discussed in detail in the following section [90].

Electrodeposition is a rapid and cost‐effective route for fabricating Ag_2_Se films. Román‐Varela et al. [180] employed an electrodeposition strategy to fabricate Ag_2_Se films with controlled thickness directly on conductive substrates. An as‐deposited film with a thickness of 880 nm annealed at 210°C exhibited a *PF* of 1169 µWm^−1^K^−2^.

Each of these methods has its distinct characteristics; for example, vacuum‐assisted filtration and drop casting enable rapid fabrication of uniform films while remaining constrained by batch processing. Screen printing, inkjet printing, and solution‐based 3D printing using suitable inks offer low cost, high throughput, and high design freedom, making them more suitable for large‐area and roll‐to‐roll manufacturing. Appropriate post‐treatment (e.g., cold pressing combining annealing or hot pressing) commonly needs to be further applied to the corresponding Ag_2_Se‐based films fabricated by vacuum‐assisted filtration, drop casting, and printing process, etc., which leads to sintering the Ag_2_Se grains and results in increased density of the films. During such post‐treatment, different microstructures (e.g., heterointerfaces, dislocations, boundaries, and pores) might also be optimized, which are beneficial for enhancing *S* and *σ*, while reducing *κ* simultaneously, so as to increase the total TE performance. Additionally, magnetron sputtering and thermal evaporation typically promote dense microstructures and preferential crystallographic orientations along (00*l*) or (0*kl*) planes, which are also beneficial for enhancing TE properties. However, the expensive equipment need to be used.

## 6 Device Assembly and Applications

As is known, FTEDs offer significant potential in energy harvesting, localized cooling, and smart sensing [213, 214], and the combination of high TE performance and mechanical flexibility makes Ag_2_Se‐based materials highly attractive for these applications [65, 215, 216].

### 6.1 Flexible Thermoelectric Devices

**FIGURE 23:**
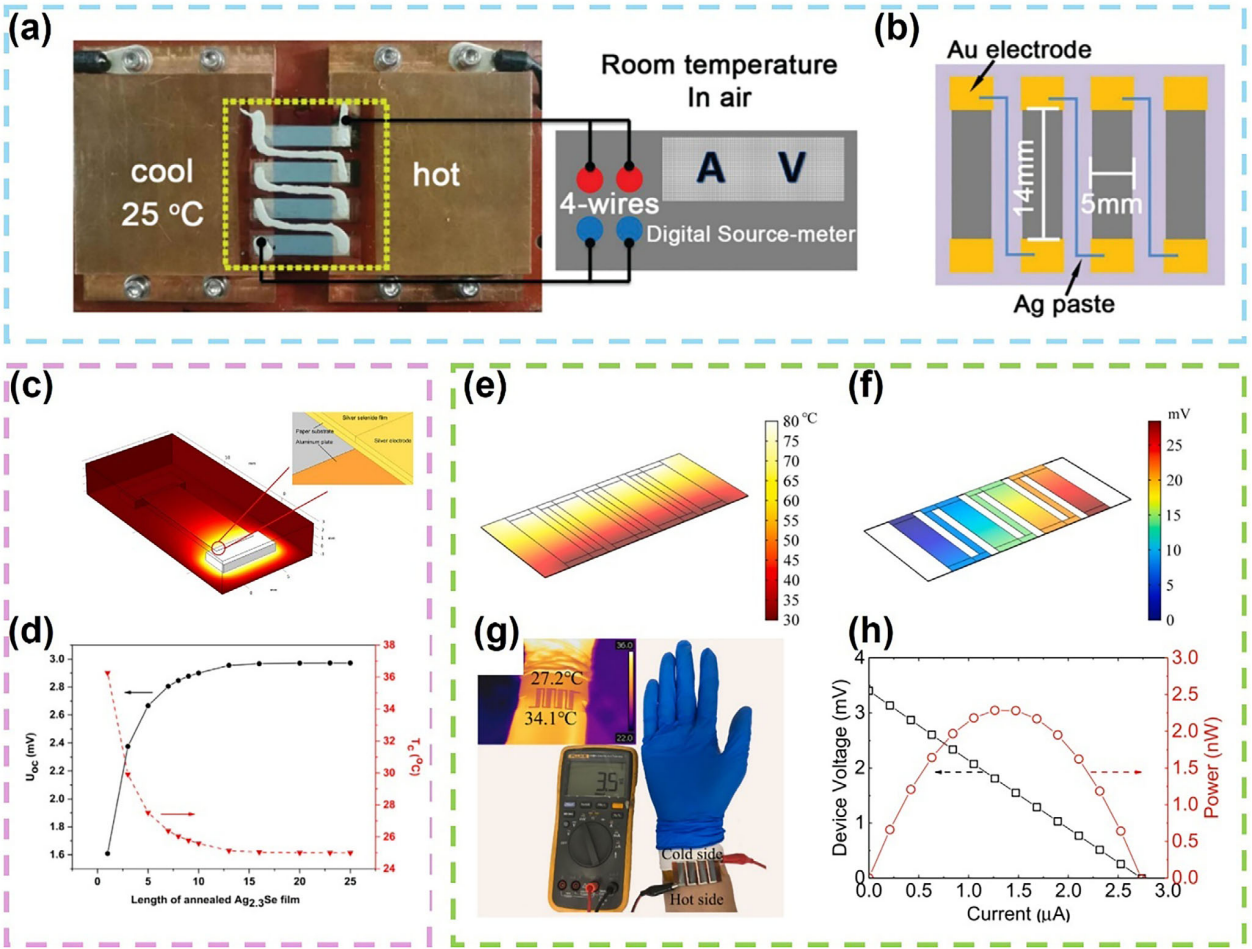
(a) Photograph of the home‐built measurement system for evaluating the output performance of FTED. (b) Schematic illustration of the FTED configuration comprising Au electrodes, Ag paste, and an Ag_2_Se film. (a, b) Reproduced with permission [73]. Copyright 2021, John Wiley & Sons. (c) Three‐dimensional model of a single Ag_2.3_Se film on paper constructed for simulation. (d) Simulated electrical and thermal performance of annealed Ag_2.3_Se films with varying lengths. (c, d) Reproduced with permission [177]. Copyright 2019, Elsevier. (e) Simulated temperature distribution and (f) measured *V*_oc_ of an Ag_1.8_Se/PI FTED under Δ*T* = 50 K. (g) Demonstration of the FTED attached to a human wrist, with inset infrared thermal image showing Δ*T*. (h) Output performance of the FTED when worn on the wrist. (e‐h) Reproduced with permission [77]. Copyright 2021, Elsevier.

In FTEDs, multiple TE legs, comprising p‐type and n‐type TE materials, are electrically connected in series and thermally connected in parallel, as shown in Figure 23a. When a temperature gradient (Δ*T*) is applied across the FTED, the generated open‐circuit voltage (*V*_oc_) can be expressed as [217, 218]:

(9) $$\begin{array}{c} V_{\mathrm{oc}}=N\left(|S_{p}|+\left|S_{n}\right|\right)\cdot \Delta T \end{array}$$

where *N* denotes the number of TE leg pairs, *S*_p_ and *S*_n_ represent the Seebeck coefficient of p‐type and n‐type TE materials, respectively.

The output power (*P*) was expressed as [217]:

(10) $$P={\left(\frac{V_{\mathrm{oc}}}{R_{\mathrm{ex}}+R_{\mathrm{in}}}\right)}^{2}R_{\mathrm{ex}}$$

where *R_ex_* and *R_in_* denote the external and inner resistances of the FTED, respectively.

A *P_max_* was achieved when *R_ex_* equals *R_in_* from Equation (10), and *P_max_* can be further given by [217, 218, 219]:

(11) $$P_{\max}=\frac{{V_{\mathrm{oc}}}^{2}}{4R_{\mathrm{in}}}$$

The maximum power density (*PD_max_*) was determined as follows:

(12) $$PD_{\max}=\;\frac{P_{\max}}{\mathrm{NA}}=\frac{S^{2}\sigma }{4l}\cdot \Delta T^{2}$$

where *A* is the cross‐sectional area of the TE leg perpendicular to its length and *l* is the effective length of the TE leg.

#### 6.1.1 Theoretical Simulation

Theoretical simulations are crucial for designing and optimizing FTEDs. Numerical methods, such as finite element analysis (FEA), allow for the prediction of the temperature distribution, output performance, and stress‐strain behavior of devices [73, 220]. These simulations provide essential guidance for optimizing the device geometry (e.g., the number and arrangement of TE legs), thereby maximizing the energy conversion efficiency [221, 222]. Figure 23 demonstrates the design and practical application of FTEDs based on Ag_2_Se films, guided by FEA simulations. Figure 23a,b show the typical output performance measurement setup, along with the photograph and schematic illustration of 4‐leg FTED, where Au electrodes were used to lower the contact resistance between TE legs and Ag paste [73].

Gao et al. [177] optimized the composition of paper‐supported Ag_2_Se films by adjusting reactant ratios of Ag/Se combined with annealing treatment, and achieved a *PF* of 2450.9 µWm^−1^K^−2^ at RT. To improve the *V*_oc_ and *P* of FTED, the ideal length of the TE leg was investigated using FEA simulations (Figure 23c,d), and the FTED assembled using the corresponding films reached a *PD*_max_ of 5.80 Wm^−2^ at a Δ*T* = 25 K [177].

Hou et al. [77] assembled a 4‐leg FTED using an Ag_1.8_Se film, yielding a *PD*_max_ of 46.8 Wm^−2^ at Δ*T* = 50 K, with < 15% resistivity change after 1000 bends with a bending radius of 5 mm. Simulated thermal maps (Figure 23e,f) illustrate a uniform temperature gradient across the device, and experimental testing confirms a stable *V*_oc_ ∼ 27.5 mV for the Ag_1.8_Se‐based FTED at Δ*T* = 50 K. Such a device is further attached to a human wrist (Figure 23g), achieving a *V*_oc_ of ∼ 3.5 mV and a *P*_max_ of ∼ 2.3 nW (Figure 23h), where a Δ*T* of ∼ 6.9 K was captured from the corresponding infrared image [77].

#### 6.1.2 In‐Plane TE Devices

**FIGURE 24:**
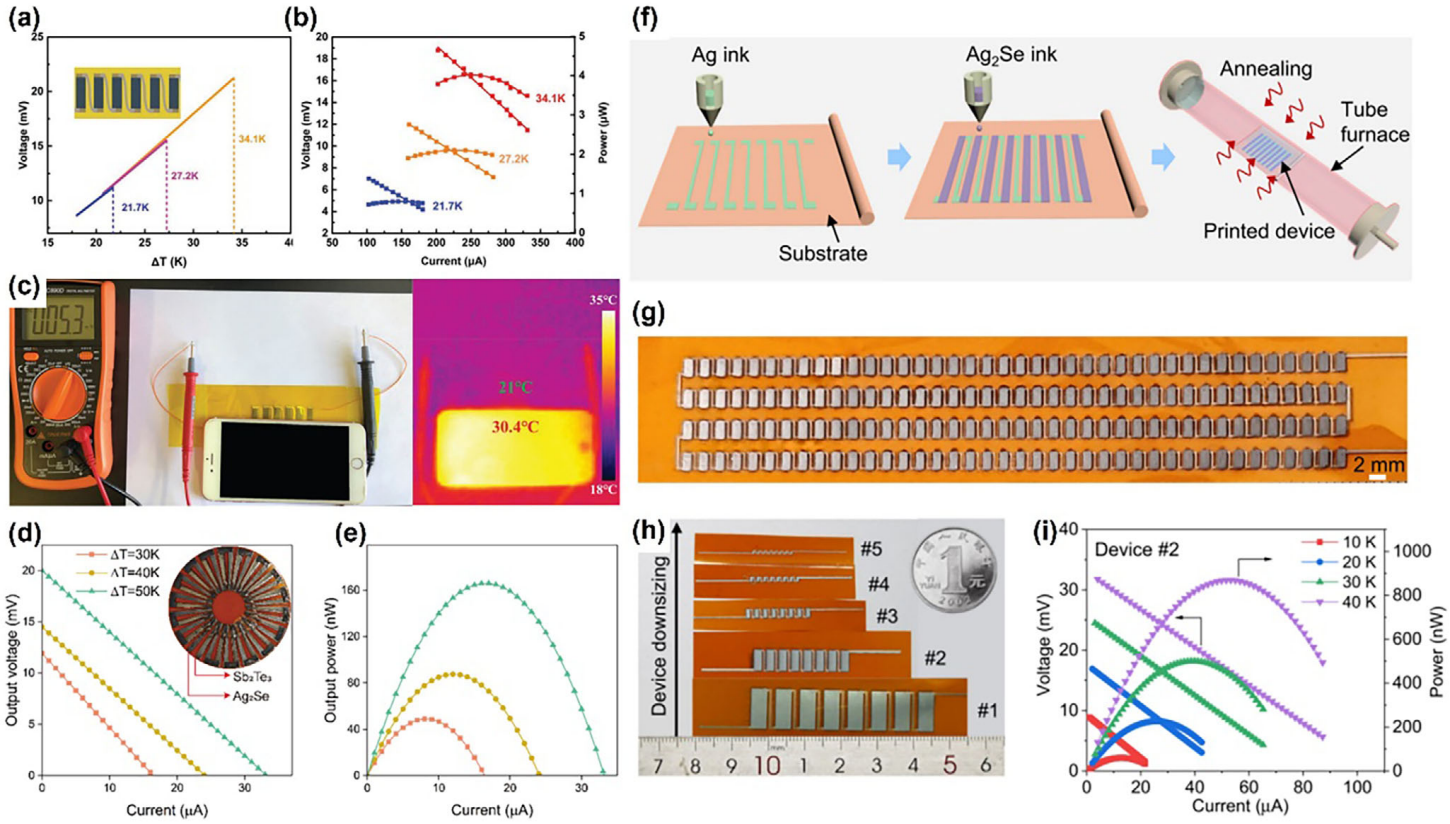
(a) Open‐circuit voltage of a 6‐leg FTED fabricated with optimized Ag_2_Se/Se/PPy film measured under different temperature gradients (inset showing diagrammatic sketch of the FTED). (b) Current‐dependent output voltage and power measured under different Δ*T* values of 21.7, 27.2, and 34.1 K. (c) Photograph of a 5.3 mV output generated from the Δ*T* between a mobile phone (after running a game) and the surrounding environment, accompanied by the corresponding infrared thermal image. (a–c) Reproduced with permission [53]. Copyright 2022, John Wiley & Sons. (d) Output voltage and (e) output power versus current of a round‐shaped Ag_2_Se/Sb_2_Te_3_ device at Δ*T* = 30, 40, and 50 K (inset showing the photograph of the round FTED with several p‐n pairs), reproduced with permission [82]. Copyright 2023, American Chemical Society. (f) Schematic fabrication workflow of a fully inkjet‐printed Ag_2_Se‐based FTED, including sequential Ag and Ag_2_Se ink deposition and post‐annealing. (g) Optical image of a fully printed device consisting of 160 Ag_2_Se TE legs with a scale bar of 2 mm. (h) Optical image of miniaturized printed devices (#1 – #5) for downscaling demonstration, with a coin for scale. (i) Output voltage and power versus current curves of Device #2 under Δ*T* = 10, 20, 30, and 40 K. (f–i) Reproduced under the terms of the Creative Commons CC‐BY Creative Commons Attribution 4.0 International license (https://creativecommons.org/licenses/by/4.0) [64]. Copyright 2024, The Authors, published by Springer Nature.

In‐plane FTEDs arrange TE elements in the same plane with an I‐type or π‐type structure [134, 141, 223, 224]. Many recent studies have focused on film performance optimization, structural design and integration density of Ag_2_Se‐based FTED [111, 137]. For example, a 6‐leg prototype FTED was fabricated using the optimized Ag_2_Se/Se/PPy films, and the *V*_oc_ scaled linearly with Δ*T* (Figure 24a). The *P*_max_ of 5.1 µW and *PD*_max_ of 6.6 Wm^−2^ were achieved at a Δ*T* of 28.8 K (Figure 24b). Additionally, the FTED was applied to harvest the dissipated thermal energy from a mobile phone after gaming, generating a *V*_oc_ ∼ 5.3 mV (Figure 24c) [53].

Besides, many FTEDs with similar structures were also assembled using such methods (cutting and connecting), which were comprised of different TE films, such as PVP/Ag_2_Se [110], PEI/Ag_2_Se [57], Ag/Ag_2_Se [137], and SWCNT/Ag_2_Se [111]. Some FTEDs with p‐type and/or n‐type legs were assembled, as summarized in Table 3 and Table 4. For example, employing p‐type Sb_2_Te_3_ films and n‐type Ag_2_Se films as the p‐n pairs, Yang et al. [61] assembled an FTED that exhibited a *V*_oc_ of 6 mV, a *P*_max_ of 65 nW, and a *PD*_max_ of 15 Wm^−2^ at a Δ*T* = 20 K.

**TABLE 3:** Output performance of FTEDs with n‐type Ag_2_Se‐based legs.

TE legs	Δ*T* (K)	*P*_max_ (µW)	*PD*_max_ (Wm^−2^)	*PD*_max_·*l*/Δ*T* ^2^ (µWm^−1^K^−2^)
Se‐doped Ag_2_S [131]	28.8	5.1	6.6	159
S‐doped Ag_2_Se [134]	31	3.98	11.06	
S‐doped Ag_2_Se [133]	28.6	5.29	10.5	
Cu‐doped Ag_2_Se [136]	32.2	6.74	28.08	541.65
Cu/S‐doped Ag_2_Se [62]	28.6	1.13	4.03	
PVP/Ag_2_Se [173]	40	233.3		
Ag_2_Se/Se/PPy [53]	34.1	4.04	37.6	
PVP/Ag_2_Se [71]	29.1	4.16	28.8	
PEDOT/Ag_2_Se/CuAgSe [145]	36	3.2	8.4	
Terpineol/Ag_2_Se [54]	34.1	7.42	16.23	
Ag/PVP/Ag_2_Se [110]	38.7	4.58	31.16	416.2
Ag_2_Se/Ag/PVP [146]	42.9	10.4	64.8	704
PEI/Ag_2_Se [57]	50	∼ 2.7	73.93	887.26
PVP/Ag_2_Se/MC [90]	36.1	2.84	22.1	
Ag/Ag_2_Se/graphene [144]	50	1.5	55.31	663.72
Ag_2_Se/carbon [143]	35.4	4.77	29.1	
Ag/Ag_2_Se [137]	29.6	6.08	∼ 13.56	
Cu_2_Se/Ag_2_Se [165]	10.3		0.0259	
Ag/Ag_2_Se [140]	42.1	8.97		
Ag/Ag_2_Se [79]	26	55.23		1089
Ag_2_Se [148]	30	3.2	22	408
Ag_2_Se [139]	40	2.801	7.78	208.8
Ag_2_Se [149]	40.6	∼1.524	∼0.47	8.472
Ag_2_Se [150]	31.4	3.42	13.5	273.7

**TABLE 4:** Output performance of FTEDs with n‐type Ag_2_Se‐based legs and p‐type TE legs.

TE legs	Δ*T* (K)	*P*_max_ (µW)	*PD*_max_ (Wm^−2^)
P: Bi_0.4_Sb_1.6_Te_3_	20	26.2	19
N: Ag_2_Se/Te [76]			
P: Sb_2_Te_3_	20	0.065	15
N: Te‐doped Ag_2_Se [61]			
P: SWCNT	35	6.1	
N: Ag_2_Se [183]			
P: Bi_0.5_Sb_1.5_Te_3_	80.7	370.88	10.72
N: Ag_2_Se [190]			
P: Cu_2_Se	60	14.75	2.34
N: Ag_2_Se [192]			
P: PEDOT	70	7	
N: Ag_2_Se [156]			

Apart from traditional rectangular FTED structures, the circular FTED structure has also been developed [82]. Zhang et al. [82] constructed a circular FTED using p‐type Sb_2_Te_3_ and n‐type Ag_2_Se film stripes (Figure 24d,e), and a *V*_oc_ of ∼ 20 mV and a *P*_max_ of 166 nW were delivered at Δ*T* = 50 K.

Increasing integration density is an effective strategy for increasing the output properties of FTEDs. Liu et al. [64] directly printed Ag_2_Se‐based FTEDs with different geometries via inkjet printing technology (Figure 24f,g). The integration densities of the FTEDs were also investigated (Figure 24h), and the *V*_oc_ of ∼ 30 mV, *P*_max_ of ∼ 0.8 µW, and an enhanced normalized *PD*_max_ of 2.0 µWcm^−2^K^−2^ for Device #2 (the normalized *PD*_max_ of 1.1 µWcm^−2^K^−2^ for Device #1) (Figure 24i) were obtained at Δ*T* of 40 K [64]. Furthermore, a 150‐leg FTED was directly printed, and a *V*_oc_ of 40 mV could be yielded by harvesting body heat. Collectively, scalable printing strategies and different device geometries expand the applicability of Ag_2_Se‐based FTEDs.

#### 6.1.3 Out‐of‐Plane Devices

Out‐of‐plane FTED arranges the TE elements along the thickness direction, utilizing a vertical Δ*T* to generate electricity. This structure usually results in enhanced output power density from a higher effective Δ*T*, although the fabrication process might be complex (e.g., cutting, soldering, and packaging) [225, 226]. Flexible substrates (e.g., PI and PDMS) or polymer encapsulation are often adopted to improve the overall device flexibility and enable reliable operation under practical bending conditions.

**FIGURE 25:**
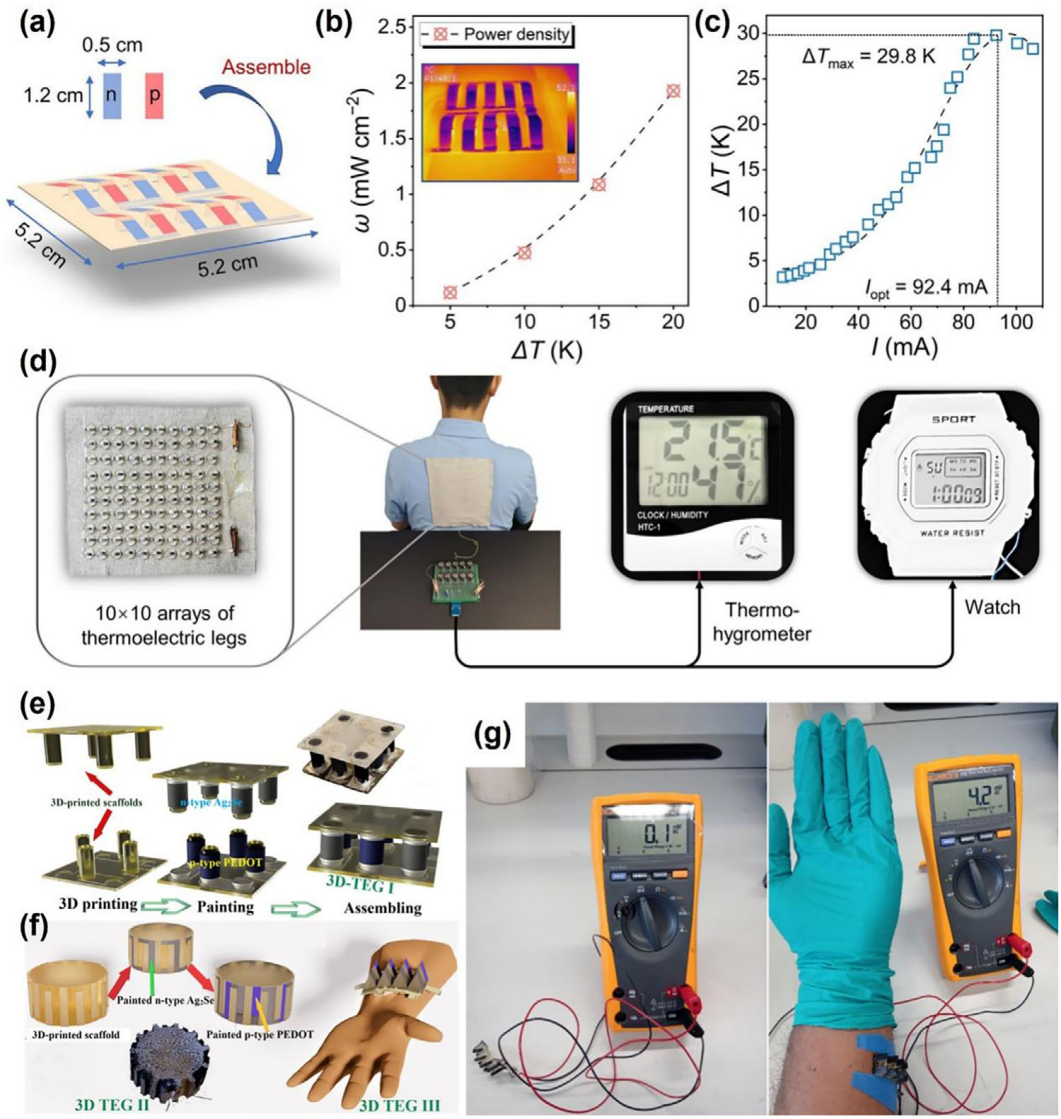
(a) Schematic illustration of the as‐prepared triangular p‐n Bi_0.4_Sb_1.6_Te_3_/Ag_2_Se FTED. (b) *PD*_max_ versus Δ*T*. The inset displays the infrared photograph showing the temperature distribution on the as‐prepared FTED after applying a Δ*T*. (c) Maximum cooling performance (Δ*T*_max_) of such FTED as a function of input current. (a‐c) Reproduced with permission [76]. Copyright 2025, Springer Nature. (d) Diagram illustrating the use of a 100‐leg out‐of‐plane FTED to charge supercapacitors and then power a thermo‐hygrometer and a watch, reproduced with permission [39]. Copyright 2025, Springer Nature. (e) Schematic diagrams for preparing 3D FTED, including sequential 3D printing, painting of TE inks, and sintering steps; (f) devices with different architectures (3D‐TED I, II, and III); and (g) Demonstration of the 3D‐TED III attached to the human body, exhibiting a *V*_oc_ of 4.2 mV at RT. (e‐g) Reproduced with permission [156]. Copyright 2020, American Chemical Society.

An out‐of‐plane FTED on PI substrate featuring a triangular p‐n junction (Bi_0.4_Sb_1.6_Te_3_/Ag_2_Se) architecture was designed by Chen et al. (Figure 25a) [76]. The device delivered a *PD*_max_ of 19 Wm^−2^ and a normalized *PD*_max_ of 4.8 µWcm^−2^K^−2^ at a Δ*T* = 20 K (Figure 25b). Such a device also exhibited cooling ability, and a Δ*T*_max_ of ∼ 29.8 K was achieved under an input current of 92.4 mA (Figure 25c). Zhang et al. [39] developed Ag_2_Se/reduced graphene oxide (rGO) composite films using vacuum‐assisted filtration and hot pressing process. The (013) preferential orientation and Ag_2_Se‐rGO interfacial effects simultaneously enhanced electrical transport and suppressed *κ*, respectively, enabling a room‐temperature *ZT* of 1.28. Based on such optimized films, a large‐scale out‐of‐plane FTED consisting of 100 TE legs was assembled (Figure 25d), delivering a normalized *PD*_max_ of ∼ 9.8 µWcm^−2^K^−2^. Under body heat conditions, the device was demonstrated to charge supercapacitors and power small wearable electronics, including a thermo‐hygrometer and a wristwatch [39].

Furthermore, Zhang et al. [192] developed a direct fabrication strategy for FTEDs, in which Ag_2_Se and Cu_2_Se films were prepared via screen printing on PI substrate combined with an in situ selenization method, significantly simplifying the conventional device fabrication process. A 3D FTED was constructed by vertically embedding the films into a silicone layer, delivering a maximum *V*_oc_ of 113 mV and a *PD_max_* of 234.3 µWcm^−2^ at a Δ*T* = 60 K. The device retained ∼ 81% of its initial *PD*_max_ after 1500 bending cycles [192]. When worn on the human arm, the *V*_oc_ of 3.6 mV and 7 mV were generated without and with forced convection, respectively.

Additive manufacturing technology was also explored to assemble 3D architectures [156]. As illustrated in Figure 25e,f, 3D‐printed scaffolds are coated with TE inks (n‐type Ag_2_Se and p‐type PEDOT) and assembled into different architecture devices (3D‐TED I, II, and III). The experimental results confirmed the 3D‐TED III prototype exhibited enhanced flexibility and output performance, where a *V*_oc_ of ∼ 4.2 mV at RT was obtained when attached directly to the human skin (Figure 25g) [156].

**FIGURE 26:**
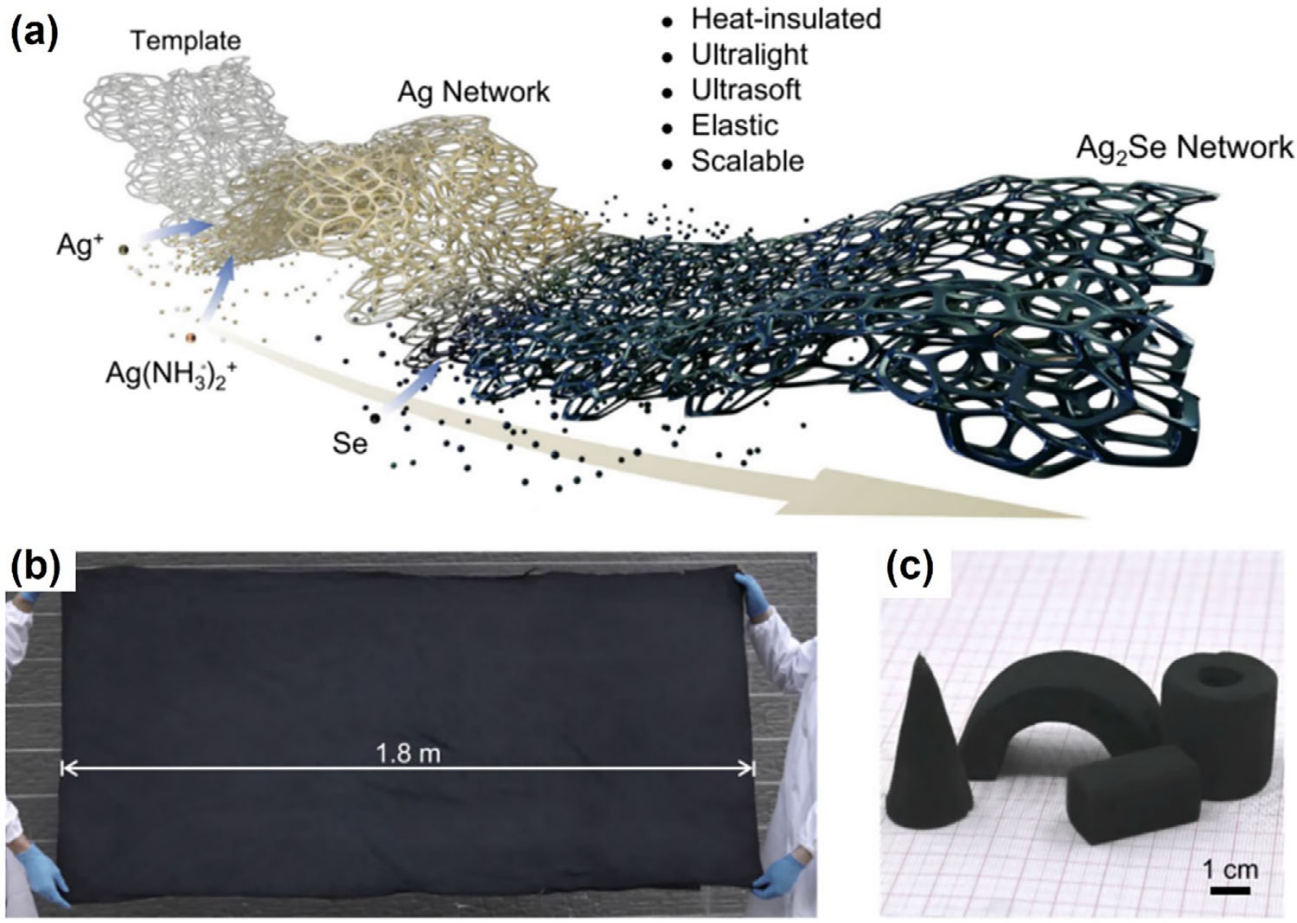
(a) Schematic illustration of the two‐step impregnation process for fabricating an Ag_2_Se network, involving in situ formation of a metallic Ag network by silvering followed by conversion to Ag_2_Se through selenization. (b) Photograph of a large‐area Ag_2_Se network sheet with dimensions of 1.8 × 0.9 m^2^, demonstrating the scalability of the process. (c) Photographs of Ag_2_Se networks shaped into various geometries, highlighting their structural flexibility and customizability. (a‐c) Reproduced under the terms of the Creative Commons CC‐BY Creative Commons Attribution 4.0 International license (https://creativecommons.org/licenses/by/4.0) [227]. Copyright 2024, The Authors, published by Springer Nature.

Figure 26 demonstrates a scalable fabrication route for an Ag_2_Se network sheet using fabric (1.8 × 0.9 m^2^), which has the advantages of high porosity, good flexibility, and soft characteristics (Figure 26a,b) [227]. The synthesis is achieved through a two‐step impregnation process, where an interconnected metallic Ag network is first formed via silvering and subsequently converted into Ag_2_Se by controlled selenization. Moreover, the networks can be easily processed into various macroscopic shapes using sponge, such as cones, arcs, and cylinders (Figure 26c). When an FTED assembled from the Ag_2_Se network was worn at ∼ 290 K, a *P*_max_ of ∼ 0.6 mW and ∼ 1 mW was generated when standing and walking at 1 m/s, respectively.

Despite the rapid progress in Ag_2_Se‐based FTEDs, the interfacial electrical/thermal contact condition remains a major challenge, as it can significantly reduce effective power output and mechanical flexibility under repeated bending and thermal cycling. In addition, mismatches between p‐type and n‐type TE legs (e.g., TE properties and mechanical flexibility) may lead to stress accumulation, interfacial delamination, and degraded device efficiency.

Addressing these issues requires interface engineering strategies, such as optimized contact materials and matched device architectures. In this context, theoretical modeling and multiphysics simulations, including electrical‐thermal‐mechanical coupling analyses, can provide valuable guidance for optimizing the contact condition, stress distribution, and heat flow pathways, thereby delivering stable and high‐efficiency FTEDs.

### 6.2 Flexible Thermoelectric Coolers

Compared with traditional rigid coolers, a flexible TE cooler (FTEC) better matches the human body or irregular surfaces, achieving efficient localized heat dissipation and temperature control utilizing the Peltier effect [228, 229]. Ag_2_Se materials, with their excellent flexibility and TE properties, provide stable cooling effects even after repeated bending of the device [230, 231].

**FIGURE 27:**
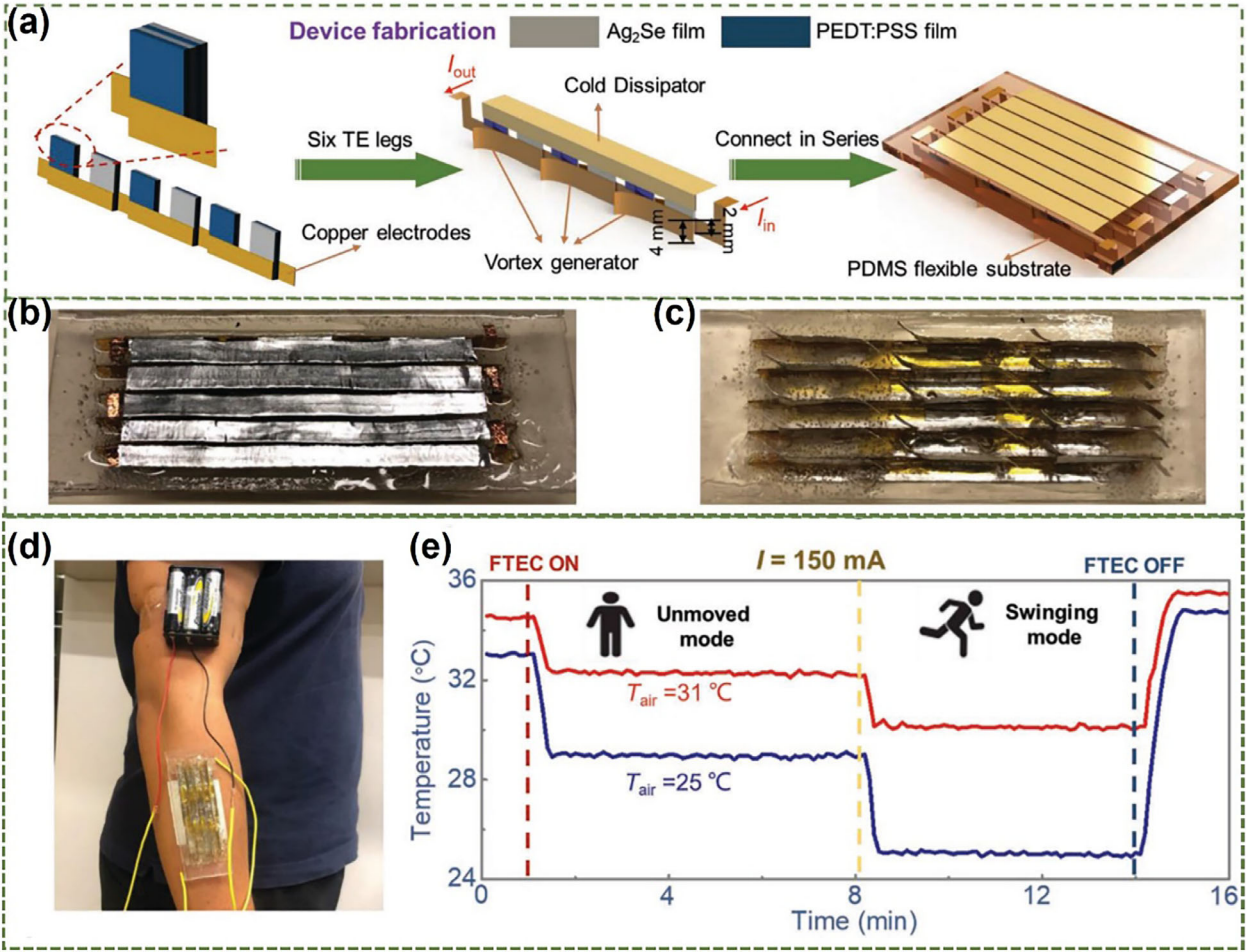
(a) Schematic flowchart of the assembly process for the FTEC, integrating Ag_2_Se films, PEDOT:PSS films, and copper electrodes on a PDMS substrate, with six TE legs connected in series. (b) Front and (c) back views of the as‐fabricated FTEC. (d) Photograph of the FTEC attached to a human arm for wearable testing. (e) Real‐time monitoring of skin temperature (*T*_skin_) during FTEC operation at an applied current of 150 mA, compared under two ambient temperatures (*T*_air_ = 25°C and 31°C) and different activity modes (unmoved and swinging). (a–e) Reproduced with permission [75]. Copyright 2022, John Wiley & Sons.

Figure 27 demonstrates the concept and performance of a wearable FTEC [75]. The device was fabricated by integrating Ag_2_Se film as n‐type element and PEDOT:PSS film as p‐type counterpart, with six TE legs assembled in series on a PDMS substrate (Figure 27a). The front and back views of the prototype (Figure 27b,c) show the uniform leg alignment and compact structural integration. The FTEC was also directly attached to a human arm for real‐time temperature monitoring (Figure 27d & e), revealing that the FTEC significantly reduces the skin temperature under both ambient conditions (*T*_air_ = 25°C and 31°C) at an applied current of 150 mA [75]. The cooling effect is maintained in both static (unmoved) and dynamic (swinging) modes, demonstrating stability during movement.

**FIGURE 28:**
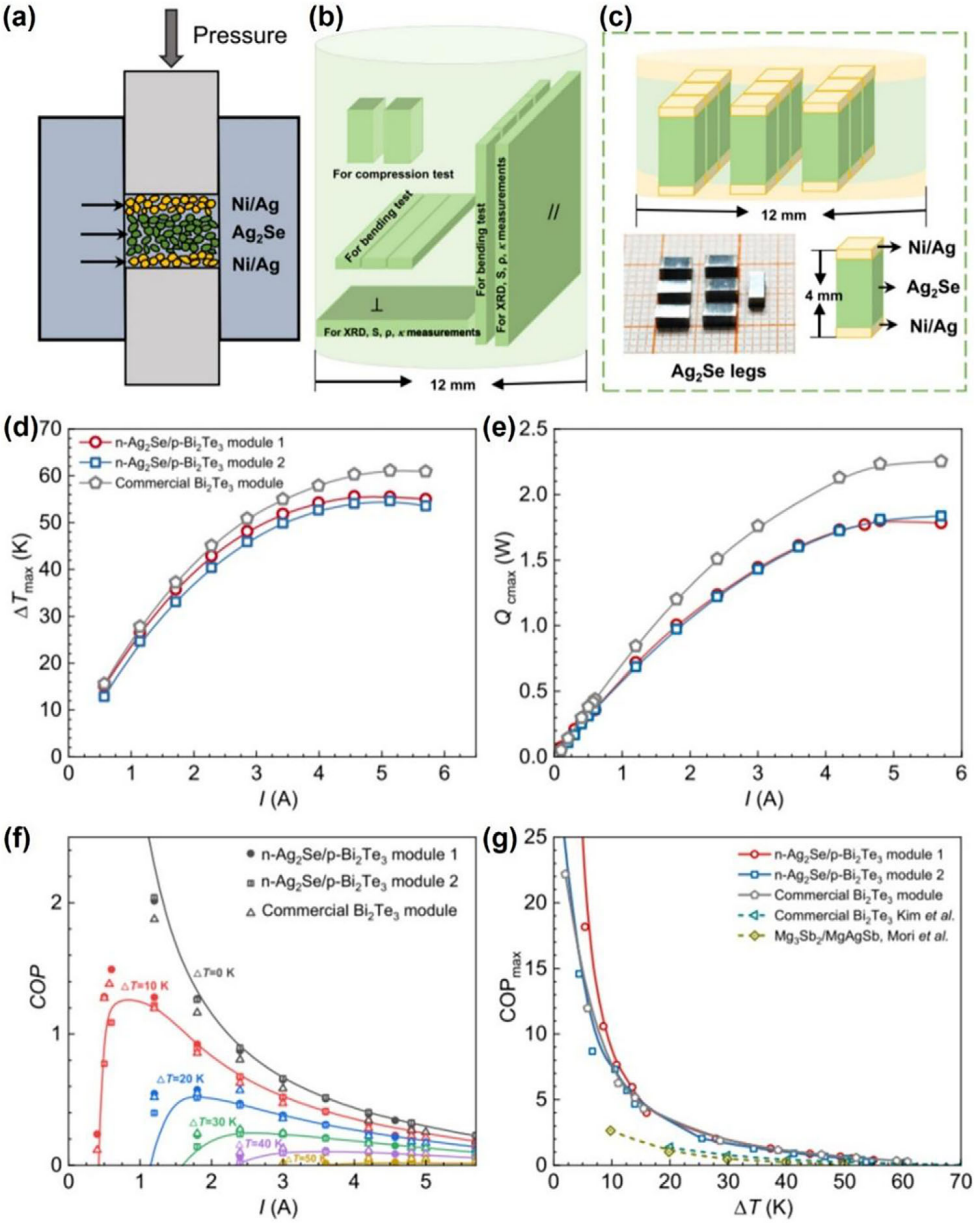
(a) Schematic of the one‐step hot‐pressing process used to prepare dense Ag_2_Se‐based legs. (b) Schematic representation of the sliced cylinder for property measurements. (c) Schematic and photograph of the Ag_2_Se legs with Ni/Ag electrode. Measured current‐dependent (d) maximum cooling temperature difference (Δ*T*_max_), (e) maximum cooling power (*Q*_cmax_), and (f) coefficient of performance (*COP*). (g) Maximum *COP* as a function of different Δ*T* for different devices. (a‐g) Reproduced under the terms of the Creative Commons CC‐BY Creative Commons Attribution 4.0 International license (https://creativecommons.org/licenses/by/4.0) [19]. Copyright 2024, The Authors, published by Springer Nature.

Liu et al. [19] prepared a TE cooler (TEC) with Ag_2_Se legs (with Ni/Ag electrodes) and commercial Bi_2_Te_3_ legs by a hot‐pressing, slicing, and assembly process (Figure 28a–c), and a maximum cooling temperature difference (Δ*T*_max_) exceeding 60 K (Figure 28d), with the maximum cooling power *Q*_cmax_ approaching 2.5 W (Figure 28e). Importantly, the coefficient of performance (COP) values (Figure 28f) indicate competitive efficiency under small Δ*T* conditions (Figure 28g) [19]. These results establish Ag_2_Se as a promising n‐type candidate for integration into practical cooling modules, promoting the advancement of high‐performance TEC.

### 6.3 Flexible Thermoelectric Sensors

Flexible TE sensors generate electrical signals based on Δ*T* to monitor environmental and human condition changes [232, 233]. The high sensitivity and flexibility of Ag_2_Se‐based TE materials render them particularly suitable for smart sensors (such as motion‐state monitoring, health‐status monitoring, and position tracking) [58, 117].

**FIGURE 29:**
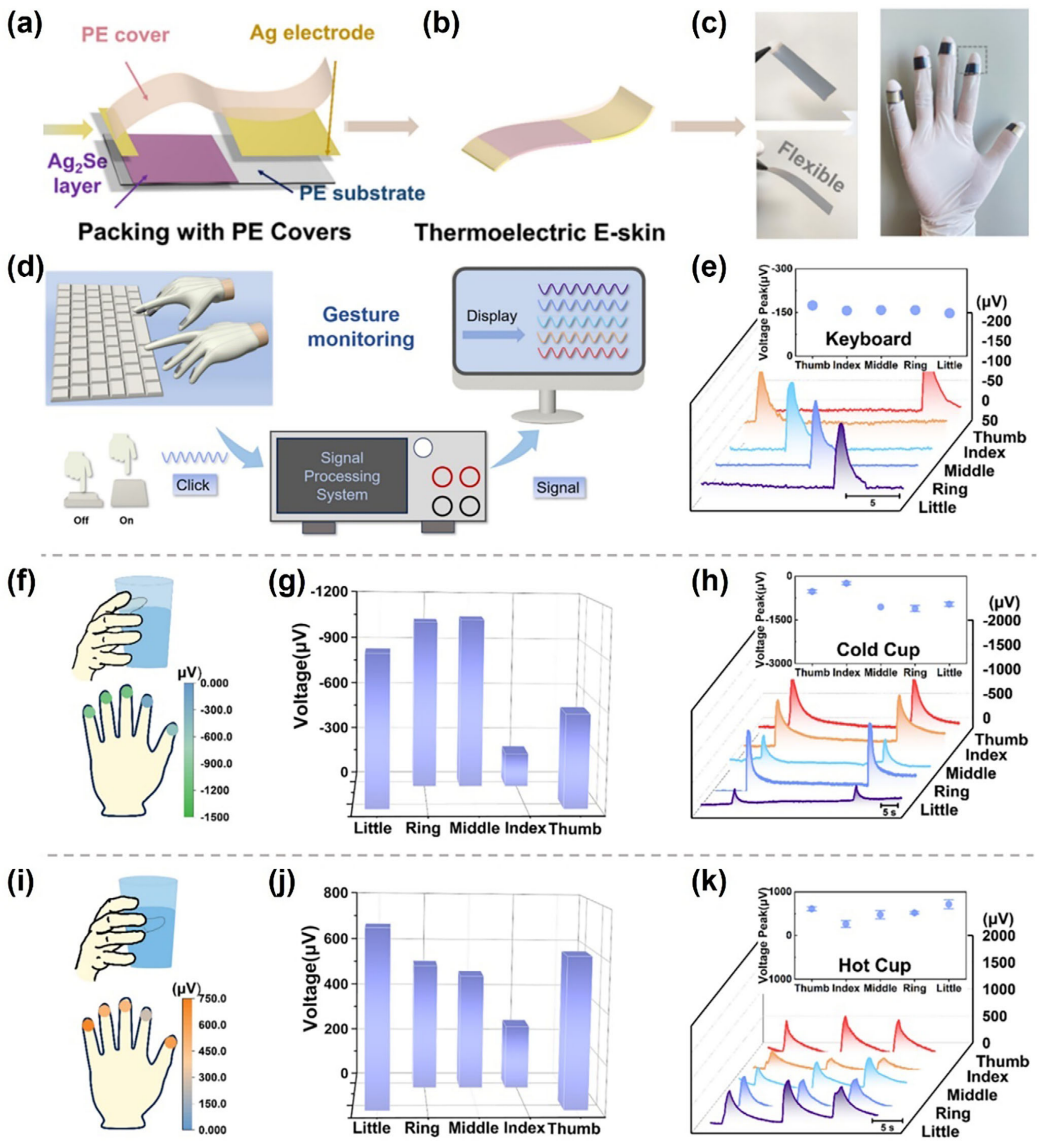
(a,b) Schematic depiction of the E‐skin fabrication process via sputtering followed by selenization. (c) Optical image of the flexible Ag_2_Se film and wearable device integrated with multiple finger sensor arrays. (d) Schematic representation of the smart glove with integrated E‐skin and signal acquisition circuit. (e) Measured *V*_oc_ response when the finger interacted with the keyboard. Gesture recognition demonstration of the smart glove when holding the (f–h) cold or (i–k) hot water cup, respectively. (a–k) Reproduced with permission [58]. Copyright 2024, American Chemical Society.

Ma et al. [58] developed a multifunctional E‐skin and smart glove based on flexible Ag_2_Se films (Figure 29a–c), and the smart glove enabled real‐time gesture recognition (Figure 29d,e), as evidenced by the distinct open‐circuit voltage signals generated during keyboard typing. The glove also exhibited strong responses to external thermal stimuli, producing characteristic signals upon contact with hot or cold objects (Figure 29f–k). These results demonstrate the capability of Ag_2_Se‐based smart glove for gesture recognition and temperature sensing, highlighting its potential for wearable electronics and human‐machine interface applications [58].

**FIGURE 30:**
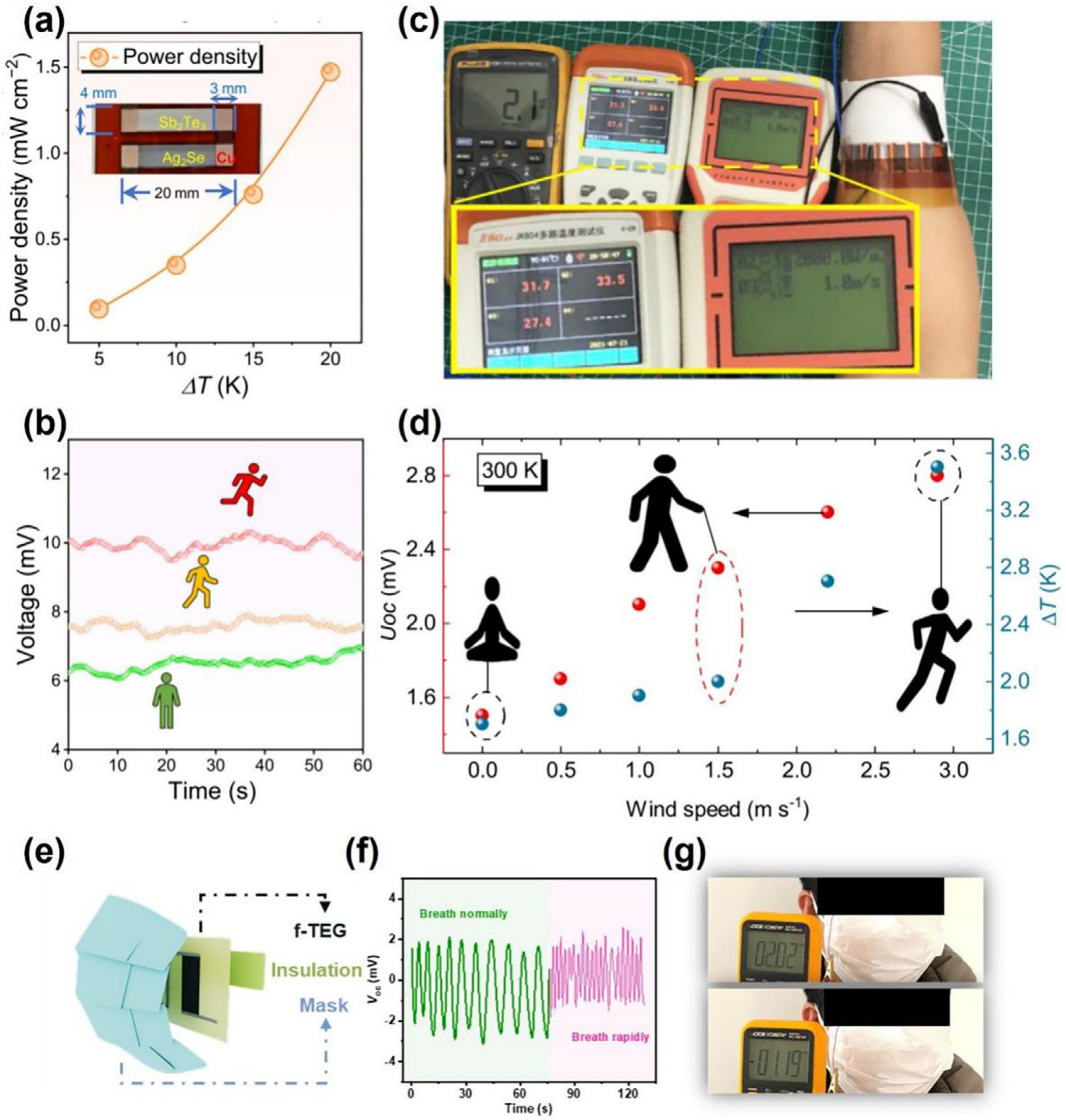
(a) Output power densities of an FTED at different Δ*T*, with an inset showing the flexible device composed of one n‐type leg (Ag_2_Se thin film with 3.2 at.% Te) and one p‐type leg (Sb_2_Te_3_ thin film). (b) Real‐time voltage output of the device when worn on the wrist during different motion states (sitting, walking, and running). (a,b) Reproduced under the terms of the Creative Commons CC‐BY Creative Commons Attribution 4.0 International license (https://creativecommons.org/licenses/by/4.0) [61]. Copyright 2024, The Authors, published by Springer Nature. (c) Photograph of a device attached to the wrist, with inset showing simultaneous measurement. (d) Dependence of voltage (left axis) and Δ*T* (right axis) on wind speed, simulating the different motion states. (c,d) Reproduced with permission [132]. Copyright 2022, Elsevier. (e) Structure of the printed FTED integrated into a face mask for respiratory monitoring; (f) *V*_oc_ versus time under normal and rapid breathing; (g) Photographs of *V*_oc_ signals generated during inhalation and exhalation. (e–g) Reproduced with permission [90]. Copyright 2025, Springer Nature.

Yang et al. [61] fabricated FTED with high output power densities at different Δ*T*. As shown in Figure 30a, the device achieves a *PD*_max_ above 1.5 mWcm^−2^ at a Δ*T* of ∼ 20 K. When worn on the wrist, the FTED produces stable voltage outputs during daily activities, with clear differences between sitting, walking, and running (Figure 30b). Hou et al. [132] presented a self‐healing FTED assembled using Cu‐doped Ag_2_Se films (Figure 30c). A systematic analysis under different airflow (Figure 30d) demonstrated that the output voltage increased with wind speed, arising from the enhanced Δ*T* from convective cooling.

Apart from the above‐mentioned assembly FTED, Qin et al. [90] demonstrated the direct printing of FTEDs via solution‐based 3D printing. When the printed FTED is attached to the skin, body heat can be harvested and converted into electrical energy. In addition, a smart mask consisting of this FTED was developed, in which respiration‐induced *V*_oc_ are detected in real time to enable monitoring of breathing conditions (Figure 30e–g), providing a potential strategy for the health management of respiratory diseases such as asthma. Moreover, distinct output signals are generated upon finger contact at different locations, enabling potential position detection [90].

### 6.4 Flexible Photo‐Thermoelectric Generators

Flexible photo‐thermoelectric generators (PTEG) can absorb heat energy generated by solar radiation and then convert it into electricity by TE effect, expanding the application scopes of traditional FTEDs [234, 235, 236]. The flexibility and high TE performance of Ag_2_Se‐based materials make them suitable for use in wearable electronics and remote sensors, which require efficient energy harvesting.

**FIGURE 31:**
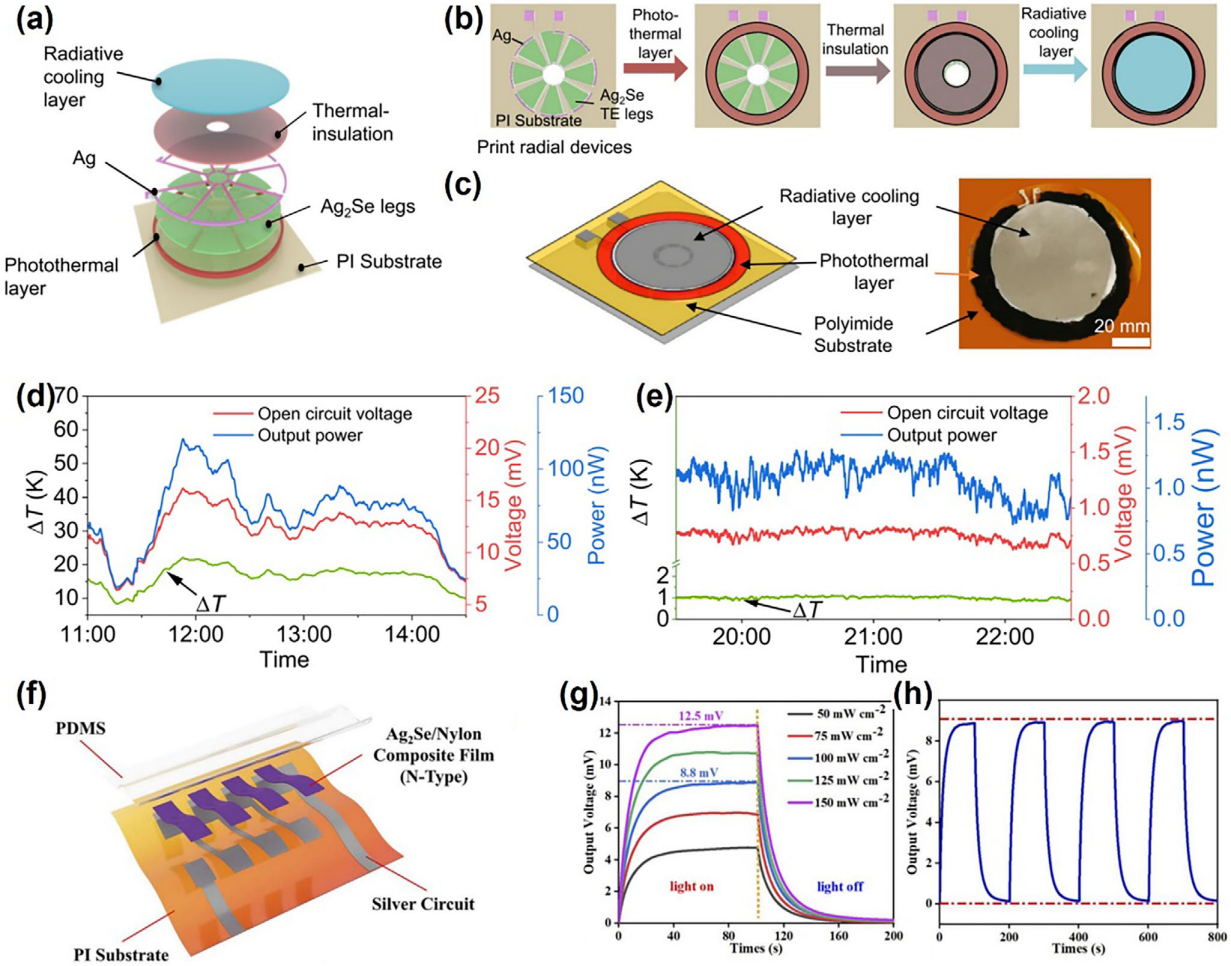
Schematic diagram of (a) the STR hybrid device, (b) the fabrication of the radial STR hybrid device on a PI substrate, and (c) the complete radial device along with its photograph. *V*_oc_ and power generated from such a STR hybrid device (d) during daytime and (e) at night. (a–e) Reproduced under the terms of the Creative Commons CC‐BY Creative Commons Attribution 4.0 International license (https://creativecommons.org/licenses/by/4.0) [64]. Copyright 2024, The Authors, published by Springer Nature. (f) Structural schematic of a PTEG. (g) The *V*_oc_ of the PTEG under 808 nm near‐infrared (NIR) irradiation with different power densities. (h) Cyclic voltage responses under repeated NIR on/off irradiation at 100 mWcm^−2^. (f‐h) Reproduced with permission [184]. Copyright 2022, Royal Society of Chemistry.

To achieve round‐the‐clock outdoor energy harvesting, Liu et al. [64] designed and constructed a solar thermal/TE/radiative cooling (STR) hybrid device integrating solar thermal, TE, and radiative cooling functionalities. The STR hybrid device (Figure 31a–c) employs a multilayer structure, where the photothermal layer translates solar energy into thermal energy, the Ag_2_Se TE layer converts heat into electricity, and the radiative cooling layer further increases the Δ*T* between the hot and cold sides. During the daytime, the STR device generates a *V*_oc_ up to ∼ 16.1 mV, while at night, a *V*_oc_ of ∼ 0.8 mV via radiative cooling was yielded (Figure 31d,e) [64].

Yang et al. [184] fabricated an Ag_2_Se‐based PTEG (Figure 31f–h) based on Ag_2_Se/nylon composite films to achieve photo‐responsive TE conversion. When irradiated with 808 nm NIR light at different intensities, the PTEG delivers voltages up to ∼ 12.5 mV (Figure 31g), and exhibits a stable, repeatable cyclic response under alternating on/off irradiation (Figure 31h). These results demonstrate that coupling TE effect with photothermal or radiative cooling is an effective strategy to fabricate an all‐day energy harvesting device, which paves a route for self‐sustained power generation systems [184].

## 7 Summary and Outlooks

Ag_2_Se has emerged as one of the most promising n‐type thermoelectric materials for flexible and wearable applications, owing to its intrinsically low *κ*, high *σ*, and ability to undergo defect and structural engineering. This review summarizes the fundamental crystallographic, electronic, and thermodynamic properties of Ag_2_Se, and the role of nanostructuring, stoichiometry tuning, doping, and introducing secondary phases in optimizing *PF* and suppressing *κ*. A wide variety of preparation techniques, such as vacuum‐assisted filtration, screen printing, magnetron sputtering, thermal evaporation, drop casting, and 3D printing have been demonstrated, providing Ag_2_Se‐based films with excellent uniformity, mechanical flexibility, and relatively high thermoelectric properties, with reported *PF* exceeding 4000 µWm^−1^K^−2^ and *ZT* values approaching ∼ 1.27. Beyond material‐level optimization, Ag_2_Se‐based films can be assembled into FTEDs with in‐plane and out‐of‐plane configurations. When combined with photothermal or radiative cooling materials, corresponding hybrid multi‐functional devices demonstrate greater potential in energy harvesting, active cooling, and sensing applications. Although significant progress was achieved in Ag_2_Se‐based flexible thermoelectric materials and devices, several challenges persist and require further attention.

1. Further optimization of materials’ thermoelectric performance is essential. Advanced defect, interface, texture engineering, etc., should be systematically combined to establish the relationships among defect chemistry, band structure evolution, carrier/phonon scattering mechanisms, and overall thermoelectric performance. Achieving this goal also requires the integration of advanced characterization techniques (e.g., in situ TEM and synchrotron‐based spectroscopy) with theoretical analysis, thereby providing rational guidance for materials’ thermoelectric performance optimization.
2. Long‐term operational stability remains a critical challenge. The chemical, structural, and thermoelectric stability of Ag_2_Se‐based films under mechanical deformation, thermal cycling, and ambient exposure must be systematically evaluated. Different strategies (e.g., composition optimization, surface coating, and encapsulation) should be developed to suppress oxidation, moisture ingress, as well as elemental migration and evaporation, thereby retaining thermoelectric and mechanical flexibility performance, and then improving durability under realistic operating conditions.
3. The development of new fabrication processes for materials and devices is highly desirable and should be further strengthened. For example, solution‐based 3D printing offers unique advantages (e.g., high design freedom, personalized customization, easy operation), which can even directly print devices, thereby simplifying fabrication steps and improving preparation efficiency.
4. Device‐level thermoelectric performance, particularly under realistic operating conditions, has enormous potential for advancements. Further improvements in power generation performance and cooling efficiency require optimized device architecture, reduced electrical and thermal contact resistance, and advanced thermal management strategies. Integrating devices with other energy conversion (e.g., photo‐thermoelectric, radiative cooling) can realize a higher Δ*T*, thereby further enhancing their thermoelectric performance.
5. Exploring new application scenarios represents an exciting direction. Future research can further expand the application of Ag_2_Se‐based thermoelectric devices into broader fields, such as fire warning, health monitoring, cell healing, etc. Even the development of integrated multifunctional devices can enable simultaneous energy harvesting, smart sensing, or thermal management.
6. AI + materials & AI + devices should be more deeply explored. Data‐driven and AI‐assisted learning methods, combined with multiscale simulations, can accelerate the optimization of compositions, defects, and microstructures at the material level, as well as architecture, thermal management, and system integration at the device level. Such AI‐enabled strategies, which provide intelligence‐driven research paradigms beyond traditional trial‐and‐error approaches, are expected to significantly shorten development cycles.
7. Standardized testing and assessment protocols are needed. Separated measurements (e.g., thermal conductivity, electrical conductivity, and Seebeck coefficient) using the corresponding individual equipment inevitably result in a higher error when compared to using the integrated equipment. Considering that the research targets in various scenarios are always different, establishing unified evaluation methods with scenario‐appropriate criteria will provide a more reliable comparison of material and device performance.

## Conflicts of Interest

The authors declare no conflicts of interest.

## Data Availability

The authors have nothing to report.
